# Metabolic impact of persistent organic pollutants on gut microbiota

**DOI:** 10.1080/19490976.2020.1848209

**Published:** 2020-12-09

**Authors:** Yuan Tian, Wei Gui, Bipin Rimal, Imhoi Koo, Philip B. Smith, Robert G. Nichols, Jingwei Cai, Qing Liu, Andrew D. Patterson

**Affiliations:** a Department of Veterinary and Biomedical Sciences, The Pennsylvania State University, University Park, PA, USA; b Huck Institutes of the Life Sciences, Huck Institutes of the Life Sciences, The Pennsylvania State University, University Park, PA, USA

**Keywords:** Persistent organic pollutants, gut microbiota, metabolomics, metatranscriptomics, NMR, mass spectrometry, flow cytometry, growth rate measurements

## Abstract

Emerging evidence supports that exposure to persistent organic pollutants (POPs) can impact the interaction between the gut microbiota and host. Recent efforts have characterized the relationship between gut microbiota and environment pollutants suggesting additional research is needed to understand potential new avenues for toxicity. Here, we systematically examined the direct effects of POPs including 2,3,7,8-tetrachlorodibenzofuran (TCDF), 2,3,7,8-tetrachlorodibenzo-*p*-dioxin (TCDD), and polychlorinated biphenyls (PCB-123 and PCB-156) on the microbiota using metatranscriptomics and NMR- and mass spectrometry-based metabolomics combined with flow cytometry and growth rate measurements (OD_600_). This study demonstrated that (1) POPs directly and rapidly affect isolated cecal bacterial global metabolism that is associated with significant decreases in microbial metabolic activity; (2) significant changes in cecal bacterial gene expression related to tricarboxylic acid (TCA) cycle as well as carbon metabolism, carbon fixation, pyruvate metabolism, and protein export were observed following most POP exposure; (3) six individual bacterial species show variation in lipid metabolism in response to POP exposure; and (4) PCB-153 (non-coplanar)has a greater impact on bacteria than PCB-126 (coplanar) at the metabolic and transcriptional levels. These data provide new insights into the direct role of POPs on gut microbiota and begins to establish possible microbial toxicity endpoints which may help to inform risk assessment.

## Introduction

Persistent organic pollutants (POPs) are a group of organic compounds and represent a global concern due to their environmental persistence (https://www.who.int/foodsafety/areas_work/chemical-risks/pops/en/). POPs have been implicated in human health problems including cancer,^1,2^ birth defects,^3,4^ immune system disorders,^5^ endocrine disruption,^6^ and reproductive disorder.^7^ Recently, laboratory studies on animals and human epidemiological studies have identified associations between POP exposure and the potential for increased risk of metabolic disorder, obesity, and/or diabetes.^8,9^

The gut microbiota are sensitive to diet, drugs, and environmental pollutants.^10^ Importantly, the toxicologic relevance of the bacteria–xenobiotic interaction for the host needs to be assessed.^11^ Several environmental chemicals have been reported to interfere with the composition of gut microbiota affecting overall gut microbiome homeostasis.^12,13^ Pollutants can affect the enzymatic capacities and metabolic activities of the gut microbiota without changing the community composition.^12^ However, it is still unclear how the gut microbiota and environmental chemicals interact and whether these interactions are relevant for human health.

**Figure 1.**
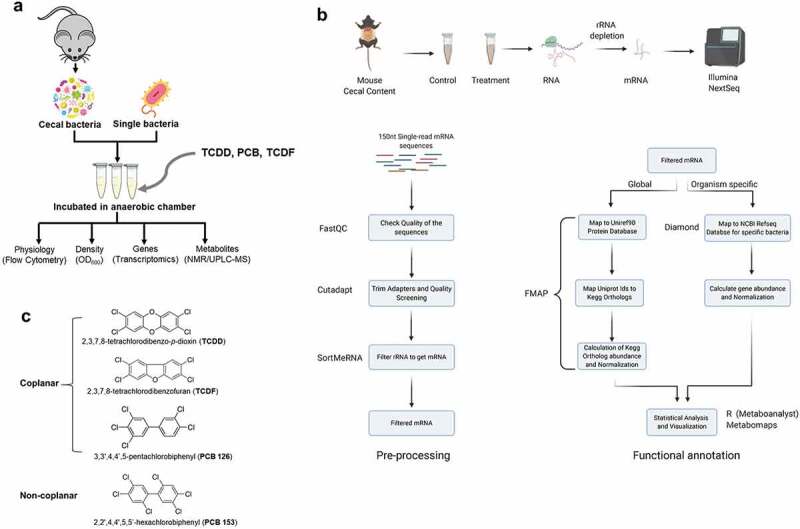
Experimental workflow for determining the direct impact of POPs on bacteria. (a) Scheme for determining the physiologic, metabolic, and transcriptional impact of four POPs on isolated cecal bacteria and individual species. (b) Metatranscriptomics workflow analysis from isolated cecal bacteria with POPs exposure (created with BioRender.com). (c) Chemical structures of four POPs used in this study

The gut microbiota exerts important effects on host homeostasis and immune functions.^14,15^ Alterations of the gut microbiota community and/or functions such as the ability to process and absorb dietary carbohydrates and complex lipids in the host are associated with various health disorders.^16,17^ A recent review suggested that gut microbiota might affect obesity and diabetes by altering human toxicodynamics including the activation, absorption, disposition, metabolism, and excretion of environmental chemicals.^18^ Our previous studies have shown that dietary POPs modified gut microbiota-host metabolic homeostasis in mice through modulation of aryl hydrocarbon receptor activity.^13,19^ Moreover, our lab investigated the functional and structural changes imparted by dietary TCDF to the gut microbiota and host using 16S rRNA gene sequencing, metabolomics, and bacterial metatranscriptomics.^20^ However, most studies to date have assessed host toxicity, while the direct effect of POPs on the gut microbiota have been explored by relatively few studies. Here, we combined flow cytometry, 16S rRNA gene sequencing, metatranscriptomics, and metabolomics to determine the responses of the gut microbiome to four POPs including TCDF, TCDD, and two PCBs (Figure 1).

Using these complementary techniques, we demonstrate that short-term exposure to POPs not only alters bacterial physiology, but also significantly alters the metabolism of the overall microbial community, in addition to gene expression. Individual bacteria species display marked variation in lipid metabolism in response to POP exposure. Exposure to a non-coplanar POP resulted in a more dramatic metabolic and transcriptional response to bacteria relative to a coplanar POP. Our results provide new insights into the direct impact of POPs on the gut microbiota.

## Results

### The physiologic and community structure effect of POPs on cecal bacterial mixtures in vitro

**Figure 2.**
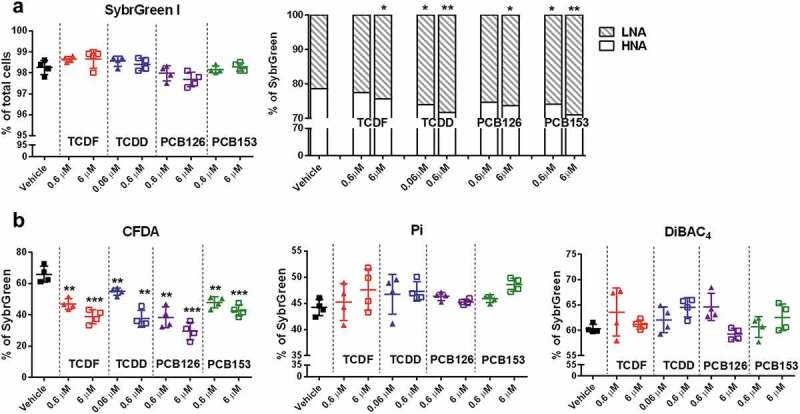
The physiological response of isolated cecal bacteria to POPs exposure in vitro. (a) Flow cytometric analyses of proportions of SybrGreen, low nuclei acid (LNA), and HNA-stained cells from isolated cecal bacteria with vehicle or two doses of POPs exposure for 4 h. (b) Flow cytometric analyses of proportions of CFDA, Pi, and DiBAC_4_-stained cells from isolated cecal bacteria with vehicle or two doses of POPs exposure for 4 h. Values are means ± S.D. (n = 4 per group). * *P* < .05, ** *P* < .01, *** *P* < .001 compare to vehicle

Cecal bacterial physiologic status after short-term exposure of POPs in vitro was assessed using a flow cytometry approach (Figure 2). A significant dose-dependent decrease in bacteria with high nucleic acid (HNA) content was observed following exposure to all four POPs (Figure 2(a)). Short-term incubation of POPs resulted in no significant changes in SybrGreen, propidium iodide (Pi), and bis-(1,3-dibutylbarbituric acid) trimethine oxonol (DiBAC_4_) stained cells but significantly decreased carboxyfluorescein diacetate (CFDA)-stained bacteria in a dose-dependent manner (Figure 2(a-b)), suggesting a rapid decrease in microbial metabolic activity without significant cell damage. Moreover, changes in the cecal community structure were observed with TCDD exposure via Generalized UniFrac analysis (Supplementary Figure 1a). Short-term TCDD exposure had significantly increased numbers of bacteria from the genus *Lactobacillus, Roseburia*, and *Oscillibacter* but decrease in the genus *Bacteroides* (Supplementary Figure 1b).

### The metabolic effect of POPs on cecal bacterial mixtures in vitro

**Figure 3.**
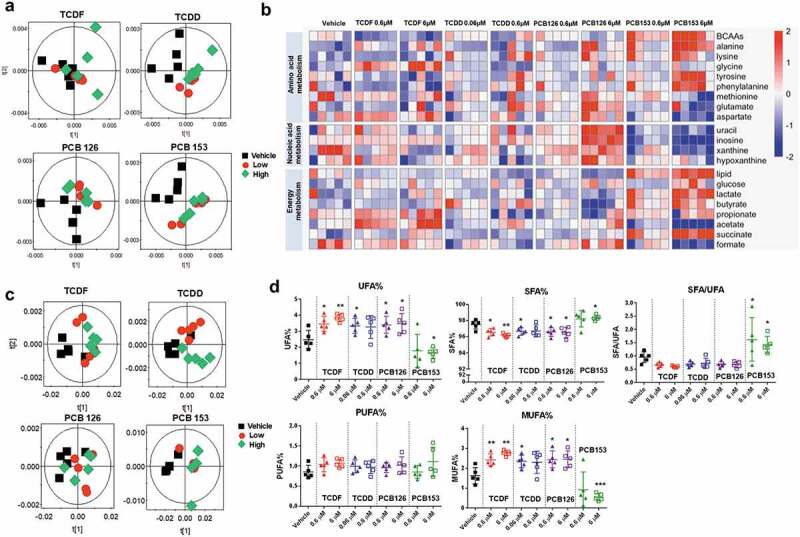
^1^H NMR-based metabolomics analysis of isolated cecal bacteria in response to POPs exposure in vitro. (a) PCA score plots obtained from ^1^H NMR data of hydrophilic metabolite profiling for the isolated cecal bacteria with vehicle or two doses of POPs exposure for 4 h. (b) Heat map representation of relative content of hydrophilic metabolites from isolated cecal bacteria with vehicle or two doses of POPs exposure for 4 h. Red shades represent metabolites that are increased, and blue shades represent metabolites that are decreased. (c) PCA score plots obtained from ^1^H NMR data of lipid profiling for the isolated cecal bacteria with vehicle or two doses of POPs exposure for 4 h. (d) The relative levels for representative fatty acids from isolated cecal bacteria with vehicle or two doses of POPs exposure for 4 h. (n = 5 per group). * *P* < .05, ** *P* < .01, *** *P* < .001 compare to vehicle

To further explore the influence of POPs on the cecal bacteria, hydrophilic metabolite and lipid profiling were performed using ^1^H NMR-based metabolomics (Figure 3). Principal component analysis (PCA) score plots showed distinct clustering of hydrophilic metabolite and lipid profiling obtained from cecal bacteria with two doses of POPs compared to vehicle (Figure 3(a) and 3(c)). PCB 153 exposure (6 µM) resulted in significantly lower levels of nucleic acids, acetate, formate, methionine, and aspartate, but significantly higher levels of glucose, lipids, lactate, butyrate, succinate, alanine, lysine, tyrosine, phenylalanine, and branched-chain amino acids (BCAAs) in cecal bacteria (Figure 3(b)). In contrast, TCDF, TCDD, and PCB 126 did not exhibit a pronounced effect on microbial hydrophilic metabolites (Figure 3(b)). Significant changes in fatty acid metabolism were observed with PCB 153 exposure (6 µM) compared to other POPs (Figure 3(d)). PCB 153 exposure (6 µM) resulted in significantly lower unsaturated fatty acids (UFA) and monounsaturated fatty acids (MUFA) but higher saturated fatty acids (SFA) and the ratio of SFA to UFA; whereas exposure to other POPs resulted in significantly lower SFA but higher UFA and MUFA (Figure 3(d)).

**Figure 4.**
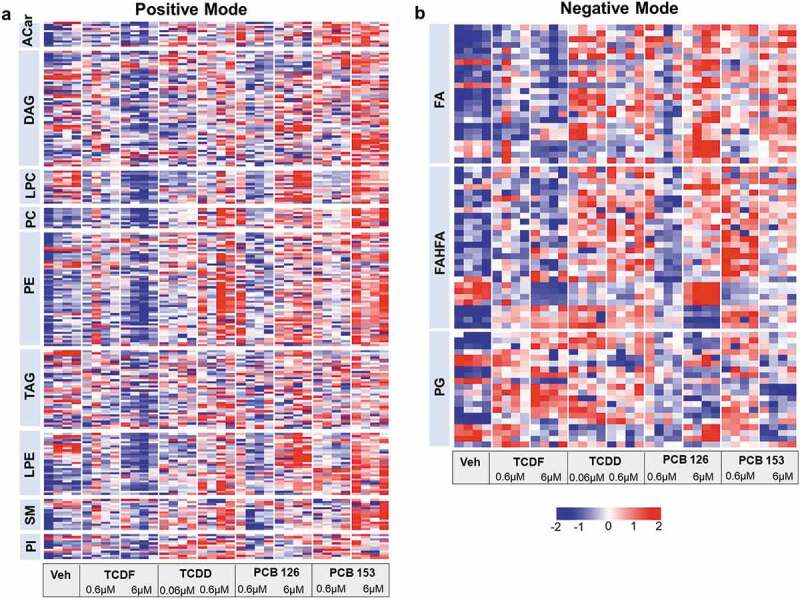
UPLC-MS/MS-based metabolomics analysis of lipid profiling from isolated cecal bacteria in response to POPs exposure in vitro. Heat map representation of the abundance of lipids from isolated cecal bacteria with vehicle or two doses of POPs exposure for 4 h from positive (a) and negative (b) modes. Red shades represent metabolites that are increased, and blue shades represent metabolites that are decreased. (n = 4 per group)

UPLC-MS analysis was performed to identify altered microbial lipids following POP exposure (Figure 4 and Supplementary Table 1–2). Significant increases in bacterial membrane lipids including fatty acid, fatty acid esters of hydroxy fatty acid (FAHFA), phosphatidylcholine (PC), phosphatidylethanolamine (PE), phosphatidylinositol (PI), phosphatidylglycerol (PG), sphingomyelin (SM), diacylg lycerol (DAG), triacylglycerol (TAG), lysophosphati dylcholine (LPC), and lysophosphatidylethanolamine (LPE) were observed following two doses (0.6 µM and 6 µM) of PCB 153 and a higher dose of TCDD (0.6 µM) and PCB 126 (6 µM) exposure (Figure 4(a-b) and Supplementary Table 1–2). Together, these results indicate that short-term incubation of POPs directly and rapidly affect cecal bacterial global metabolism that is associated with significant decreases in microbial metabolic activity.

### The transcriptional effect of POPs on cecal bacterial mixtures in vitro

**Figure 5.**
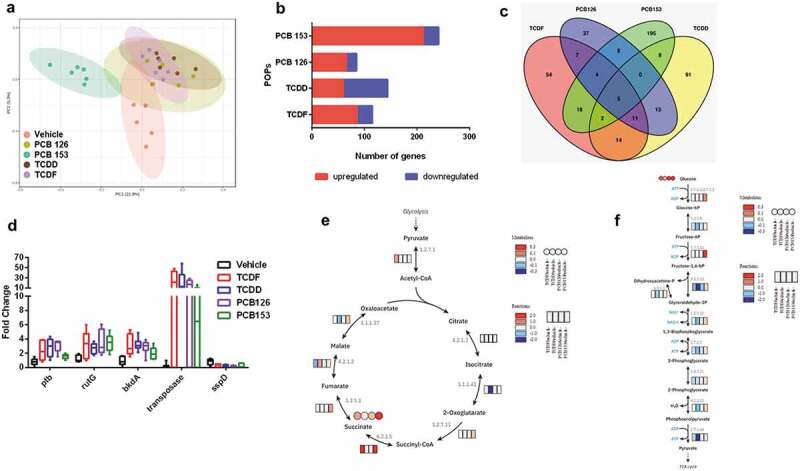
The transcriptional response of isolated cecal bacteria to POPs exposure in vitro. (a) PCA plot of gene expression of cecal bacteria with vehicle or POPs exposure for 4 h (*p*-value = 0.002 from each POP compared to vehicle). (b) The Number of differentially expressed gene orthologs for the isolated cecal bacteria with vehicle or POPs exposure for 4 h. (c) Number of pollutant-specific gene orthologs shared between each POP. (d) The gene orthologs significantly regulated in response to all four of POPs. (e) Integrated and visualized tricarboxylic acid (TCA) cycle using metatranscriptomics data combined with metabolomics data. (f) Integrated and visualized glycolysis pathway using metatranscriptomics data combined with metabolomics data. (n = 6 per group)

We investigated the bacterial functional response to POPs by analyzing the metatranscriptome from cecal bacteria with TCDF (6 µM), TCDD (0.6 µM), and PCBs (6 µM) exposure compared to vehicle using RNA-seq. PCA showed distinct separations between cecal bacteria with vehicle and POP exposure with the greatest differences occurring between PCB 153 and other POP (*p*-value = 0.002 of PCA models from each POP compared to vehicle) (Figure 5(a)). The number of differentially expressed gene orthologs differed among samples exposed to different POPs (Figure 5(b)). The number of upregulated gene orthologs varied from 60 to 212 for TCDD (0.6 µM) and PCB 153 exposure (6 µM), while the number of downregulated gene orthologs varied from 19 to 84 for PCB 126 (6 µM) and TCDD exposure (0.6 µM) (Figure 5(b)). Among the differentially expressed gene orthologs, most were found to be specifically changed only by one POP (Figure 5(c)). We identified five gene orthologs that were significantly regulated in response to all four POPs corresponding to phosphate butyryltransferase (ptb), small acid-soluble spore protein D (sspD), putative pyrimidine permease RutG (rutG), 2-oxoisovalerate dehydrogenase E1 component (bkdA), and transposase IS5 family (Figure 5(d)), which are associated with butyrate metabolism,^21^ BCAAs degradation,^22^ and pyrimidine degradation^23^ as well as DNA cleavage and modification,^24^ respectively.

Analysis of the KEGG module and pathway enrichment confirmed and extended these trends (Table 1). Pathways including tricarboxylic acid (TCA) cycle, glycolysis, amino acid biosynthesis, nucleotide metabolism, and fermentation pathways were integrated and visualized using metatranscriptomics data combined with metabolomics data (Figure 5(e-f) and Supplementary Fig. 2–4). Although each POP induced specific transcriptomic responses, some general trends were shared, such as transcript levels related to the TCA cycle, carbon metabolism, carbon fixation pathways, and protein export (Table 1). For example, the TCA cycle and glycolysis pathways were significant enriched by exposure to most POPs, especially PCB 153 (6 µM), supported by significantly higher levels of gene orthologs and intermediates in pathways (Figure 5(e-f)). Of particular note, PCB 153 exposure (6 µM) resulted in distinct changes in bacterial transcript levels compared to other POPs, such as fatty acid biosynthesis, lipopolysaccharide (LPS) biosynthesis, vitamin B6 metabolism, mismatch repair, and base excision repair (Table 1).

**Table 1.** Pathway enrichment analysis of metatranscriptomic data

Pathways	TCDF	TCDD	PCB 126	PCB 153
Tricarboxylic cycle (TCA cycle)	**		***	*
Carbon metabolism	***	**	**	*
Propanoate metabolism	***	***		
Carbon fixation pathways	**		**	*
Glutathione metabolism	*		*	
Amino sugar and nucleotide sugar metabolism	*	*		
Pyruvate metabolism	*	**		*
Biosynthesis of amino acids	*			
Pentose phosphate pathway	*	**		
Biosynthesis of secondary metabolites	*			
Protein export	*		*	**
Phosphotransferase system (PTS)		****	*	
Fructose and mannose metabolism		**	**	
Styrene degradation		**		
Butanoate metabolism		**		*
Glycolysis/Gluconeogenesis		**		
Purine metabolism		*		
Flagellar assembly		*		*
Glycosaminoglycan degradation		*		
Central carbon metabolism in cancer		*		
ABC transporters		*		
Lipoic acid metabolism		*		**
Bacterial chemotaxis			*	*
Homologous recombination				***
Vitamin B6 metabolism				***
Mismatch repair				**
Lipopolysaccharide biosynthesis				**
Starch and sucrose metabolism				**
DNA replication				*
Fatty acid biosynthesis				*
Pyrimidine metabolism				*
Glycine, serine and threonine metabolism				*
Base excision repair				*

Pathway enrichment analysis using the metatranscriptomic results comparing vehicle with POPs exposure. *P* value was calculated by a student’s t test. * *p* < 0.05, ** *p* < 0.01, *** *p* < 0.001, **** *p* < 0.0001

To further explore the transcriptomic responses of specific bacteria following POP exposure, five representative bacteria including *Bacteroides, Clostridium, Lactobacillus, Bifidobacterium*, and *Fusobacterium* were mapped to their reference sequences (Supplementary Table 3). The differentially expressed gene orthologs induced by four POPs compared to a vehicle from these five genera are listed in Supplementary Tables 4–8. In total, we identified 125 gene orthologs differentially expressed after TCDF exposure (6 µM), 96 gene orthologs differentially expressed after TCDD exposure (0.6 µM), 98 gene orthologs differentially expressed after PCB 126 exposure (6 µM), and 234 gene orthologs differentially expressed after TCDF exposure (6 µM) in *Bacteroides* (Supplementary Table 4). *Bifidobacterium* and *Fusobacterium* have less differentially expressed gene orthologs by POPs exposure, which could be due to the reduced mRNA that mapped to their reference sequences (Supplementary Tables 3 and 7–8). Consistent with isolated cecal bacteria results, PCB 153 exposure resulted in a greater impact on these five individual bacteria than other POPs at the transcriptional levels (Supplementary Tables 3–8).

### The physiologic and metabolic effect of POPs on individual bacteria species in vitro

Having defined the transcriptomic responses of specific bacteria with POPs exposure, we sought to explore the physiologic and metabolic effect of POPs on individual bacteria. Gram-negative and Gram-positive bacteria are have different cell wall structures that play a variety of protective and adaptive roles.^25,26^ In order to explore the specific effect of POPs on Gram-negative and Gram-positive bacteria, we combined growth rate measurements (OD_600_) and flow cytometry with UPLC-MS-based metabolomics to investigate the responses of POPs on six individual bacteria species. Six bacterial species were used including three Gram-positive bacteria (*Lactobacillus paracasei, Bifidobacterium longum*, and *Clostridium ramosum*) and three Gram-negative bacteria (*Fusobacteria nucleatum, Bacteriodes fragilis* (638 R), and *Bacteriodes fragilis* (ATCC®25282^TM^)), which are found in the gut of healthy individuals and play a key role in host metabolism.^27,28^

**Figure 6.**
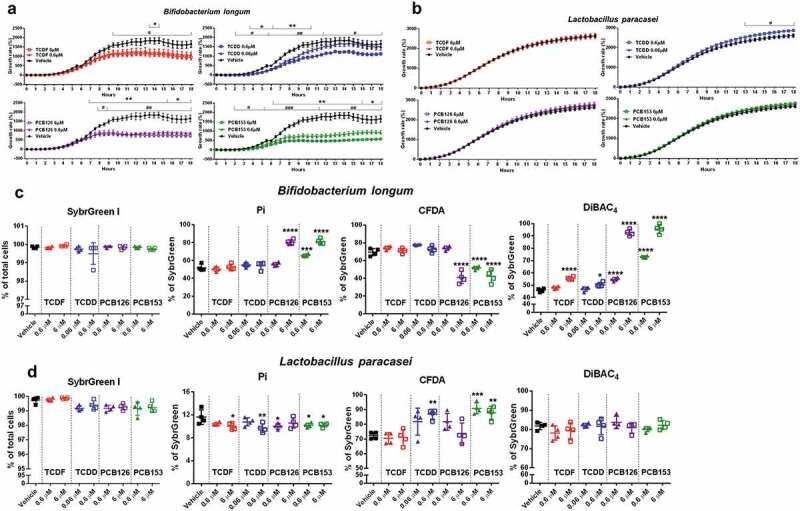
The growth rate and physiological response of individual species to POPs exposure in vitro. (a-b) Growth rate of *Bifidobacterium longum* (a) and *Lactobacillus paracasei* (b) with vehicle or two doses of POPs exposure as measured by absorbance (OD_600_) over 18 hours.Values are means ± S.D. (n = 5 per group), *P < .05, ** *P* < .01, *** *P* < .001, lower dose compare to vehicle; # *P* < .05, ## *P* < .01, ### *P* < .001 higher dose compare to vehicle.(c-d) Flow cytometric analyses of proportions of SybrGreen, Pi, CFDA, and DiBAC_4_-stained cells from *Bifidobacterium longum* (c) and *Lactobacillus paracasei* (d) with vehicle or two doses of POPs exposure for 18 h. (n = 4). * *P* < .05, ** *P* < .01, *** *P* < .001, **** *P* < .0001 compare to vehicle

We combined flow cytometry with optical density (OD_600_) over 18 h to monitor the impact of POPs on the growth and physiologic status of six bacteria species (Figure 6 and Supplementary Figure 5–6). No significant effects were observed in the growth rate and physiologic status in most bacteria species including *Bacteriodes fragilis* (ATCC®25282^TM^), *Bacteriodes fragilis* (638 R), *Fusobacteria nucleatum*, and *Clostridium ramosum* following exposure to two doses of four POPs for 18 h (Supplementary Figure 5–6), indicating no significant cell damage following POP exposure to those bacteria species. The significant decreases in the growth of *Bifidobacterium longum* were observed with a higher dose of TCDF (6 µM) and TCDD (0.6 µM) and two doses of PCB 126 and PCB 153 exposure, while subtle decreases were observed with a lower dose of TCDF (0.6 µM) and TCDD (0.06 µM) exposure (Figure 6(a)). Consistent with the OD_600_ results, two doses of PCB 153 (0.6 µM and 6 µM) and a higher dose of PCB 126 (6 µM) exposure significantly increased the proportions of damaged cells (Pi and DiBAC_4_-stained cells) and decreased CFDA-stained bacteria (Figure 6(c)), suggesting dramatic changes to the physiological status of *Bifidobacterium longum* after 18 h POPs exposure. POPs exposure did not result in dramatic changes in the growth of *Lactobacillus paracasei* but a subtle increase in the growth of *Lactobacillus paracasei*, which is indicated by the increased growth rate and a significant decrease in Pi-stained bacteria with POPs exposure (Figure 6(b) and 6(d)).

**Figure 7.**
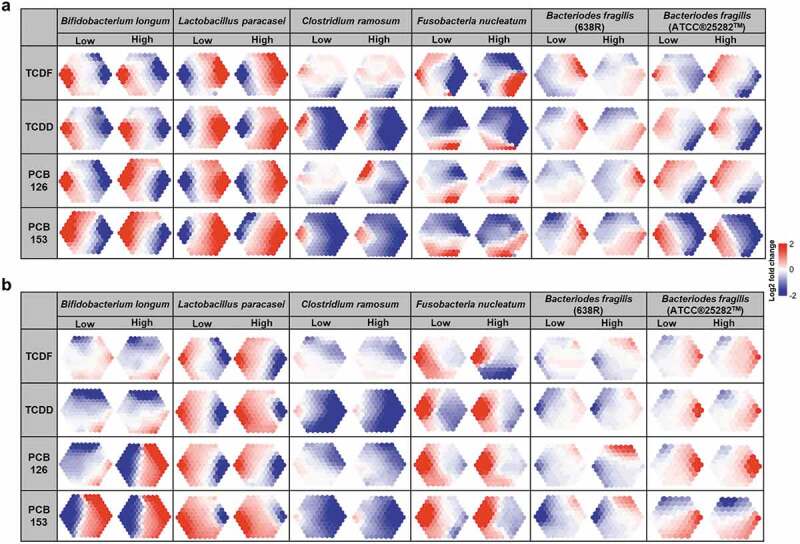
UPLC-MS/MS-based metabolomics analysis of lipid profiling from individual species to POPs exposure in vitro. Lipids were automatically arranged within an optimal supra-hexagon using the Suprahex package for R/Bioconductor. The map preserves the input information and provides the underlying cluster details such as the dimensionality, distribution, distance, clusters, and identity of metabolites (Supplementary Figure 7–18). The map also functions to place the most significantly changed metabolites on the outer portion of the map. MetaPrints based on the lipid profiling derived from UPLC-MS/MS positive (a) and negative (b) modes of *Bifidobacterium longum, Lactobacillus paracasei, Clostridium ramosum, Fusobacteria nucleatum, Bacteriodes fragilis* (ATCC®25282^TM^), and *Bacteriodes fragilis* (638 R), denoted as the log_2_ fold change relative to vehicle, following POPs exposure for 18 h. (n = 4 per group)

Having determined the significant changes in lipid profiling in cecal bacteria with POP exposure, we sought to investigate the influence of POPs on the lipid metabolism of these six bacteria species (Figure 7 and Supplementary Figure 7–18). To visualize the patterns of lipid metabolism of six bacteria species following POP exposure, the log_2_ fold change values of lipid profiles obtained from UPLC-MS were assembled using self-organizing maps (SOMs) and projected onto suprahexagonal landscapes (Figure 7 and Supplementary Figure 7–18). These maps display the “metabolic fingerprint” for six bacteria species following POP exposure and provide a means to visually interpret complex lipid changes. The six bacteria species showed extreme variation in lipid composition in response to TCDD, TCDF, or PCBs exposure in both positive and negative modes, with increases of most lipids in *Lactobacillus paracasei* with POPs exposure and decreases of most lipids in *Clostridium ramosum* with POPs exposure (Figure 7, Supplementary Figs. 8–9, and Supplementary Figs. 14–15). Notably, two strains of *Bacteriodes fragilis* also showed different responses to POP exposure, with *Bacteriodes fragilis* (ATCC®25282^TM^) being more sensitive (Figure 7, Supplementary Figs. 11–12, and Supplementary Figs. 17–18). Moreover, most individual bacteria species, such as *Bifidobacterium longum, Clostridium ramosum, Fusobacteria nucleatum*, and *Bacteriodes fragilis* (ATCC®25282^TM^) are more sensitive to (non-coplanar) PCB-153 than (coplanar) PCB-126 (Figure 7). It is interesting to note that limited significant effects in hydrophilic metabolites were observed following exposure to two doses of POPs in *Lactobacillus paracasei* and *Bifidobacterium longum* (Supplementary Figs. 19–20 and Supplementary Tables 9–10).

## Discussion

Concerns regarding the effects of POPs on the gut microbiota have increased in recent years.^10,12,18^ Mounting laboratory animal studies report that POP exposure might alter the microbial community structure and/or the metabolic activities, leading to host disorders.^13,20,29^ In this study, we investigated the direct impact of four POPs on the gut microbiota at the physiological, metabolic, and transcriptional levels using in vitro systems. The four POPs used in this work: (i) TCDF, considerable shorter half-life in rodents;^13^ (ii) TCDD, the most potent aryl hydrocarbon receptor agonist;^30^ and (iii) two PCBs (coplanar PCB 126 and noncoplanar PCB 153), are known to have a different degree of toxicity.^31^ We report a systematic study that probes the direct interactions between POPs and gut microbiota and provide a new perspective of POP toxicity.

The direct metabolic and physiologic responses of cecal bacteria to short-term POP exposure were observed in vitro. Short-term POPs exposure led to a dose-dependent decrease in metabolic activity in cecal bacteria that is associated with increased low nucleic acid (LNA) bacteria and decreased high nucleic acid (HNA) bacteria. LNA populations have a decreased rate of metabolic activity compared to HNA.^32^ Similar observations have been reported with antibiotics and anti-inflammatory drug studies that showed the effective antibacterial activity in vitro.^32,33^ However, no significant cell damage was observed with POP exposure in cecal bacteria, supported by no changes in SybrGreen, Pi, and DiBAC_4_ stained cells. It is interesting to note that the analysis of microbial metabolism after short-term POPs exposure revealed disturbances in multiple metabolic pathways, which were not fully consistent with what we observed in vivo with TCDF oral exposure.^13^ These variations between in vitro and mouse studies could be attributed to the host-microbiota axis that involves crosstalk between the host and the microbiome. This notion is supported by the observation that TCDF-mediated changes are aryl hydrocarbon receptor (AHR)-dependent as no significant changes in the fecal or cecal content of *Ahr-*null mice following TCDF exposure were observed.^13^

One of the most prominent findings in this study was the profound changes in bacterial lipid profiles following POPs exposure. POPs are mainly lipid-soluble chemicals that accumulate in the membrane bilayer between the acyl chains of fatty acids and increase membrane fluidity.^34^ An increase in lipid saturation is the most common mechanism used to modulate membrane fluidity, which helps cells survive under long-term adverse conditions.^34,35^ Consistently, our data showed that PCB 153 exposure resulted in an increased ratio of bacterial saturated to unsaturated fatty acids and significantly changed fatty acid biosynthesis, suggesting a rapid change in bacterial membrane fluidity in response to PCB 153. It is interesting to note that six individual bacteria species including three Gram-positive bacteria (*Lactobacillus paracasei, Bifidobacterium longum*, and *Clostridium ramosum*) and three Gram-negative bacteria (*Fusobacteria nucleatum* and two *Bacteriodes fragilis*) show extreme variation in lipid metabolism following exposure to the four POPs used in this study. The reason for this variation might be partially attributed to adaptation mechanisms for bacteria to survive under adverse conditions. Bacterial strains surviving in the environment must cope with the toxic compounds and counteract damage to the cytoplasmic membrane and cell wall that represents the initial contact between the cell and toxic compounds.^34^ Surprisingly, we did not observe a distinct pattern of lipid metabolism in Gram-positive and Gram-negative bacteria by POPs exposure, despite the difference in the cell wall structure. This observation suggests more studies are warranted to determine the factor(s) important for the heterogeneous response of bacteria to POPs.

Another prominent finding in this study was the integrated and detailed characterization of the altered metabolic pathways using metatranscriptomics data combined with metabolomics data. Oral POP treatment modulated the microbial community,^13,20^ which was not fully consistent with what we observed in the in vitro study. The differences could be due to intervention duration and/or the lack of host-mediated processes in in vitro systems. We identified general transcriptional responses to most POPs related to energy metabolism pathways including the TCA cycle, butyrate metabolism, and pyruvate metabolism, supported by our metabolomics data. Similar observations have been reported in a previous mouse study^20^ and an in vitro study that reported a dose–dependent inhibitory effect and a disruption in bacterial fermentation processes by PCB 126 exposure using a mouse fecal culture.^36^ The direct in vitro effects may translate into microbiome shifts in vivo, which contribute to the disruption of glucose tolerance in host with POPs exposure.^36^ The disturbances of bacterial energy metabolism pathways might affect a range of host processes including energy utilization, host-microbe signaling, and anti-inflammatory effects.^13,37^ Protein export is an essential function for a variety of bacteria to eliminate toxic byproducts, produce, and excrete essential growth factors, and acquire nutrient.^38^ Disruption in protein export was also observed with POP exposure, indicating the disruption in the bacterial secretion system by POPs exposure might lead to the disruption in the host innate immune system.^39^ Protein transport systems in Gram-positive and Gram-positive bacteria are varied, due to the differences in their cell envelope structure.^40^ It will be interesting to see whether POPs cause varied responses in protein transport to Gram-positive and Gram-positive bacteria in the future work. Notably, our results also emphasized many distinct transcriptional responses of PCB 153 on the cecal bacteria, including the induction of bacterial membrane biogenesis,^41^ stress response pathways,^20^ and defense of DNA damage.^42^

Exposure to (non-coplanar) PCB-153 resulted in a more dramatic metabolic and transcriptional response to bacteria relative to (coplanar) PCB-126. Coplanar PCB-126 has similar biological properties to TCDD, showing toxic effects primarily through activation of AHR.^43,44^ The non-coplanar PCBs, on the other hand, exhibit different biological activities and more complex routes of toxicity.^45^ Interestingly, our data showed that PCB-153 had the strongest effects on bacteria, whether assessing metabolic response or gene expression. This could be partially explained by higher membrane fluidity caused by PCB-153, resulting in a greater impact on bacterial membrane properties and membrane destruction.^46^ This observation was consistent with the experiments performed on liposomes,^46^ cell lines,^47^ and model bacterial membranes,^48^ indicating the physiological effects of non-coplanar POPs may be greater compared to coplanar POPs. These in vitro findings need to be tested rigorously in vivo (in animal models) to better appreciate the importance of the host response.

Many environmental pollutants influence the gut microbiota yet quantifiable or biologically meaningful endpoints that reflect this toxicity have not been evaluated. As those used for animal or human studies, such as alanine aminotransferase (ALT) and aspartate transaminase (AST) for liver damage or C-reactive protein (CRP) for muscle damage, the toxicity endpoints for understanding the response of the microbiome to the environmental pollutants need to be assessed. The results described here identify possible new avenues for probing microbial toxicity by environment pollutant exposure.

## Materials and methods

### Chemicals

POPs including TCDD, TCDF, PCB-126, and PCB-153 (Figure 1(c)) were purchased from Cambridge Isotope Laboratories, Inc. (Tewksbury, MA) and suspended in dimethyl sulfoxide (Sigma-Aldrich, St. Louis, MO). The fluorescent dyes including SybrGreen, propidium iodide (Pi), carboxyfluorescein diacetate (CFDA), and bis-(1,3-dibutylbarbituric acid) trimethine oxonol (DiBAC_4_) were ordered from Sigma-Aldrich (St. Louis, MO) and Invitrogen (Carlsbad, CA).

### In vitro bacterial culture and incubation

Cecal microbiota incubation studies were modified from previously described protocols.^33^ Briefly, cecal contents were isolated from 7-week-old C57BL/6 J male wild type mice and diluted with brain heart infusion broth (1 g in 10 ml). The cecal suspensions were incubated with two doses of TCDD (high: 0.6 µM and low: 0.06 µM), TCDF (high: 6 µM and low: 0.6 µM), and PCBs (high: 6 µM and low: 0.6 µM) at 37°C for 4 h. The high dose (6 µM) of TCDF and PCBs is equivalent to the dose of TCDF that we have previously published in vivo studies.^13,20^ After incubation, bacterial samples were washed and stained for flow cytometry analysis or kept at −80°C for other analyses.

Six individual bacteria species including *Bifidobacterium longum* (ATCC®15707^TM^), *Lactobacillus paracasei* (ATCC®25303^TM^), *Fusobacteria nucleatum subsp. nucleatum* (ATCC®25586^TM^), *Clostridium ramosum* (ATCC®25582^TM^), *Bacteriodes fragilis* (ATCC®25282^TM^), and *Bacteriodes fragilis* (638 R) were cultured and treated with two doses of TCDD (high: 0.6 µM and low: 0.06 µM), TCDF (high: 6 µM and low: 0.6 µM), and PCBs (high: 6 µM and low: 0.6 µM) at 37°C for 18 h (for more detailed methods about bacteria culture, see Supplemental Material). The effects of POPs on the growth rate of different bacteria in vitro were measured by optical density (OD_600_) using a Multiskan Sky Microplate spectrophotometer (Thermo Fisher Scientific, Waltham, MA). Flow cytometry and metabolite analysis were performed after 18 h of POPs incubation. All experiments were performed in a monitored anaerobic chamber (Coy Laboratory Products, 95% N_2_, 5% H_2_) and repeated at least three times.

### Flow cytometry analysis

After incubation, the bacterial mixtures and individual bacteria species were washed twice and resuspended with 1X reduced PBS (1 mg/ml L-cysteine). Bacterial physiology was assessed by four fluorescent dyes: SybrGreen that stains all nucleic acids regardless of cellular damage, Pi that stains only dead or damaged cells, CFDA that stains for enzymatic/metabolic activity, and DiBAC_4_ that stains damaged bacteria.^33,49^ All cytometric analyses were performed using a BD Accuri™ C6 plus flow cytometer (BD Biosciences, San Jose, CA) and data were analyzed with FlowJo V10 software (FlowJo, LLC). The percentages of Pi, CFDA, DiBAC_4_, low nucleic acid (LNA), and high nucleic acid (HNA) stained cells were calculated relative to the total bacterial counts obtained by SybrGreen staining.

### ^1^H NMR-based metabolomics experiments

The hydrophilic metabolites and lipids from 1 ml of bacteria were extracted twice with 1 ml pre-cooled methanol/H_2_O (2:1, v/v) or chloroform/methanol (2:1, v/v), followed by three freeze-thaw cycles. After evaporation, the extracts were reconstituted in 500 µl of 0.1 M PBS containing 100% D_2_O and 0.005% (v/v) 3-(trimethysilyl) [2,2,3,3–^2^H_4_] propionate (TSP) (hydrophilic metabolites) or deuterated chloroform containing 0.03% (v/v) tetramethylsilane (TMS) (lipids) and analyzed with a Bruker Avance NEO 600 MHz spectrometer equipped with an inverse cryogenic probe (Bruker Biospin, Germany) at 298 K. A typical 1D NMR spectrum named NOESYPR1D was acquired for both hydrophilic and lipid extracts. Metabolite assignments were carried out on the basis of a set of 2D NMR spectra and published results.^19,49^

All ^1^H NMR spectra were phase- and baseline-corrected and referenced to TSP (*δ* = 0.0) for hydrophilic metabolites or TMS (*δ* = 0.0) for lipids using TopSpin 3.6 (Bruker Biospin). The spectra were integrated into 0.004 ppm-width buckets using the AMIX 3.8 (Bruker Biospin). Principal component analysis (PCA) was performed using the SIMCA 13.0.3 (Umetrics, Umea, Sweden). Heatmaps were plotted using RStudio (pheatmap). The relative levels for representative fatty acids including the molar percentages of unsaturated fatty acids (UFA%), saturated fatty acids (SFA%), polyunsaturated fatty acids (PUFA%), monounsaturated fatty acids (MUFA%), and SFA-to-UFA ratio (SFA/UFA) were also calculated as previously reported.^50^ These calculations were based on the spectral integral areas (Supplementary Fig. 21) for – C***H***= C***H*** – (from UFA, *δ* 5.38), – C***H_3_*** (from all fatty acids, *δ* 0.84), = CH–C***H_2_***–CH = (from PUFA, *δ* 2.76) taking into consideration of proton numbers; the signal area for SFA and MUFA was calculated by subtracting that for UFA from 1 and PUFA from UFA.

### LC-MS based metabolomics experiments

Bacteria species samples (0.6 ml) were extracted with 1 ml of pre-cool chloroform/methanol (2:1, v/v). After homogenization, the samples were added 250 µl of HPLC water and vortexed, followed by centrifugation (22,000 rpm, 4°C for 10 min). The top phase was collected for hydrophilic metabolites and the upper phase was collected for lipid analysis. Two phases were dried in a vacuum and reconstituted in 60 μl of 3% methanol containing 1 µM chlorpropamide (hydrophilic metabolites) or 100 μl of isopropanol/acetonitrile/H_2_O (50:25:25, v/v/v) containing 1 µM 15:0–18:1-d7-PC (lipids). After centrifugation, supernatants were transferred to autosampler vials for LC-MS analysis.

Hydrophilic metabolite analysis was performed with a Dionex Ultimate 3000 quaternary HPLC system connected to Exactive^TM^ Plus Orbitrap mass spectrometer (Thermo Fisher Scientific, Waltham, MA) with a Hydro-RP C18 column (2.1 × 100 mm × 2.5 µm particle size; Phenomenex, Torrance, CA). Samples for lipid profiling were separated by reverse-phase HPLC using a Vanquish UHPLC system (Thermo Fisher Scientific, Waltham, MA) with a Waters (Milford, MA) CSH C18 column (1.0 × 150 mm × 1.7 um particle size). The eluate was delivered into an Orbitrap Fusion Lumos Tribrid™ mass spectrometer using a H-ESI™ ion source (all Thermo Fisher Scientific) (for more detailed methods about LC-MS, see Supplemental Material). LC-MS data were analyzed by the open-source software MS-DIAL.^51^ MetaMapp network analysis for hydrophilic metabolite was performed to visualize and integrate biochemical and chemical similarities as previously described.^52^ The colors of each node such as red and blue represent up- or down-regulation compared to the vehicle. The size and shape of nodes represent fold-change and class of metabolites. The log2 fold changes of lipids from each POPs exposure compared to the vehicle group were trained a self-organizing map (SOM) and projects onto a two-dimensional suprahexagon using the Suprahex package for R/Bioconductor.^53^ These suprahexagons arrange related metabolites within nodes or small hexagons, that are further arranged based on vector weight. The map preserves the input information and provides the underlying cluster details such as the dimensionality, distribution, distance, clusters, and identity of metabolites.

### Metatranscriptomic analysis

The cecal bacterial metatranscriptomic analysis was done as previously described^20^ (Figure 1(b)). Briefly, total RNA was isolated from 1 ml of cecal bacterial mixtures by 1 ml of Trizol and purified with RNeasy® Mini Kit twice (QIAGEN, Hilden, Germany). The total RNA was measured and checked by an Agilent Bioanalyzer. The 16S and 23S rRNA fractions were removed from total RNA using the RiboMinus^TM^ Bacteria Transcriptome Isolation kit (Invitrogen, Carlsbad, CA) and purified with RNeasy® MinElute® Cleanup Kit (QIAGEN, Hilden, Germany). The deletion of 16S and 23S rRNA was checked again with the Agilent Bioanalyzer. The rRNA depleted bacterial RNA samples were submitted to the Pennsylvania State University Genomic Sequencing core for sequencing. TruSeq Stranded mRNA kit was used to make a uniquely indexed library from each sample and then sequenced on Illumina NextSeq High-Output 150 nt single read sequencing run.

The obtained sequences were subjected to quality check using FastQC with a minimum Phred score of 30. Low-quality sequences and adapters were removed using Cutadapt^54^ with a quality cutoff of Phred score 30 and adapter sequences recommended for TruSeq Stranded mRNA kits. The quality-controlled trimmed reads were then subjected to ribosomal RNA (rRNA) filtering using SortMeRNA.^55^ Using the Silva database,^56^ all the sequences identified as rRNA were removed to obtain a pool of non-ribosomal rRNA. The non-ribosomal rRNA were then subjected to functional annotation using FMAP.^57^ Briefly, the non-ribosomal rRNA was mapped to the Uniref90 protein database using Diamond.^58^ The uniport ids were mapped to KEGG Orthologs and the abundances of KEGG Orthologs were calculated using FMAP with default parameters. The counts were normalized to the total number of reads in each sample. Exploratory analysis was done using MicrobiomeAnalyst.^59^ PerMANOVA (adonis function in vegan R package) was used to evaluate the effect of treatment on Bray-Curtis distance matrices with 999 random permutations. Each treatment group (TCDD, TCDF, PCB-126, and PCB-153) was individually compared to the vehicle group. Differential abundance analysis was tested using Mann-Whitney-Wilcoxon tests. Multiple test correction was performed by calculating False Discovery Rate-adjusted *p*-value using the Benjamini-Hochberg method with a threshold of p-value <0.05 and p.adjust <0.5. The significantly different KEGG Orthologs were selected for visualizations and further analysis.

Investigations into the effect of POPs on specific bacteria in the gut microbiome was carried out using a custom pipeline. Sequences pertaining to five representative genera of gut microbiota: *Bacteroides, Clostridium, Lactobacillus, Bifidobacterium*, and *Fusobacterium* were downloaded from NCBI Reference sequence databases. Filtered mRNA were mapped to these reference sequences using Diamond^58^ using default parameters. The abundance of each gene was calculated by counting the number of hits in the reference database. The counts were normalized to the total number of reads in each sample. For each bacterium, differentially expressed gene orthologs for each POP were calculated using the student *t*-test. Multiple test corrections were performed by calculating False Discovery Rate-adjusted *p*-value using the Benjamini-Hochberg method with a threshold of p-value <0.05 and p.adjust <0.05.

### Metatranscriptomic and metabolomics data integration and visualization

Metabolomics data and metatranscriptomics data were visualized using MetaboMaps.^60^ Pathways were condensed into a custom figure. The enzymes were mapped to gene orthologs. Log2Fold changes in metabolite and gene orthologs were plotted on the graph as circle and box heatmaps.

### Sanger sequencing and 16S rRNA gene sequencing analysis

Bacteria DNA from bacterial mixtures and individual bacteria species was extracted using the E.Z.N.A. stool DNA kit (Omega Bio-Tek Inc., Norcross, GA). Bacterial species used throughout the study were confirmed by Sanger sequencing as previously described^61^ and the result was analyzed with BLAST (Supplementary Table 11). The DNA from cecal bacterial mixtures was analyzed using 16S rRNA gene sequencing as described.^62^ Briefly, the extracted bacterial DNA was amplified using the V4V4 primer set (515 F and 806 R). The product quality was assessed using 1% agarose gel electrophoresis and a DNA 7500 LabChip on the Agilent 2100 Bioanalyzer (Agilent Technologies, Santa Clare, CA). The sequencing was performed on the Illumina MiSeq platform at Penn State Genomics Core Facility (University Park, PA) and analyzed using the mothur platform.^63^ The Generalized Unifrac analysis was performed using a phylogenic tree and operational taxonomic unit (OTU) table by R studio.^63^

### Statistics

All data values are expressed as mean ± standard deviation (SD). Graphical illustrations and statistical analyses were performed using GraphPad Prism 6.0 (GraphPad, San Diego, CA). One-way analysis of variance (ANOVA) followed by Dunnett’s multiple comparisons was used.

## Supplementary Material

Supplemental Material
