# Supplementary material for: Metabolic impact of persistent organic pollutants on gut microbiota
Source: Gut Microbes. 2020 Dec 9;12(1):1848209. doi: 10.1080/19490976.2020.1848209 (PMC7734116; doi:10.1080/19490976.2020.1848209)
Supplement: Supplemental Material [file KGMI_A_1848209_SM2623.docx]

**Metabolic Impact of Persistent Organic Pollutants on Gut Microbiota**

Yuan Tian^a#^, Wei Gui^a#^, Bipin Rimal^a^, Imhoi Koo^a^, Philip B. Smith^b^, Robert G. Nichols^a^, Jingwei Cai^a^, Qing Liu^a^, Andrew D. Patterson^a*^

*^a^Department of Veterinary and Biomedical Sciences, The Pennsylvania State University, University Park, Pennsylvania 16802, United States*

*^b^Huck Institutes of the Life Sciences, The Pennsylvania State University, University Park, Pennsylvania 16802, United States*

^#^These authors contributed equally to this work

^*^To whom correspondence should be addressed. Email: [adp117@psu.edu](mailto:adp117@psu.edu). Address: 322 Life Science Bldg, University Park, PA 16802, Phone: 814-867-4565

**Materials and Methods**

*Bacteria media preparation*

1. Media preparation for *Bifidobacterium longum* and *Clostridium ramosum*

Recipe for 100 ml: 1 g meat extract, 0.3 g yeast extract, 1 g peptone, 0.5 g glucose, 0.1 g soluble starch, 0.5 g sodium chloride, 0.3 g sodium acetate, and 50 mg L-cysteine in 100 ml water, adjust pH (6.8 ± 0.2) and autoclave sterilize.

2. Media preparation for *Lactobacillus paracasei*

Recipe for 100 ml: 5.5 g *Lactobacillus* MRS broth in 100 ml water and autoclave sterilize.

3. Media preparation for *Fusobacteria nucleatum subsp. Nucleatum* and *Bacteriodes fragilis* (ATCC®25282^TM^)

Recipe for 100 ml: 3.7 g brain heart infusion broth in 100 ml water and autoclave sterilize.

4. Media preparation for *Bacteriodes fragilis* (638R)

Recipe for 100 ml: 3.7 g brain heart infusion broth, 0.5 mg hemin, and 100 mg L-cysteine in 100 ml water and autoclave sterilize; after autoclave, add 2 ml 2% NaHCO_3_, 100 µl rifampicin (200 µg/ml), 200 µl gentamicin (100 µg/ml), and 20 µl erythromycin (10 µg/ml).

*UPLC-MS experiments for lipid extracts*

Samples (5 µl) were separated by reverse phase HPLC using a Vanquish UHPLC system (Thermo Fisher Scientific, Waltham, MA) with a Waters (Milford, MA) CSH C18 column (150 mm x 1 mm 1.7 µm particle size) maintained at 65 ℃ and a 15 minute gradient, at a flow rate of 110 µl/min. Solvent A was 40% HPLC grade water and 60% HPLC grade acetonitrile with 0.1% formic acid and 10 mM ammonium formate, Solvent B was 90% HPLC grade isopropanol and 10% HPLC grade acetonitrile with 0.1% formic acid and 10 mM ammonium formate. The initial condition were 85% A and 15 % B, increasing to 30% B at 2 min, 48% B at 2.5 min., 82% B at 11 min, and 99% B at 11.01 min where it was held at 99% B until 12.95 min before returning to the initial conditions at 13.00 min. The eluate was delivered into an Orbitrap Fusion Lumos Tribrid™ mass spectrometer using a H-ESI™ ion source (all Thermo Fisher Scientific). The mass spectrometer was scanned from 200-1000 m/z at a resolution of 120,000 and operated in polarity switching mode for the first 12 min, and in positive mode only for the last three minutes. The capillary voltage was set at 4 kV in positive ion mode, and 2.5 kV in negative ion mode with an RF lens value of 60%, and an AGC target of 4 x 105 with a maximum injection time of 50 ms. Product ion MS/MS spectra were acquired in acquireX mode, with one exclusion blank, an inclusion sample injection and then three subsequent sample injections. The parent ion was isolated using the quadrupole with an isolation window of 1.5 m/z, a stepped HCD (10,25,40 V) was used for activation, the mass spectrometer was operated at a resolution of 7500, and 3 microscans were acquired for each MS2 spectra.

*UPLC-MS experiments for hydrophilic extracts*

Metabolomics profiling was performed with a Dionex Ultimate 3000 quaternary high performance liquid chromatography (HPLC) pump, column compartment, and autosampler-coupled Exactive Plus Orbitrap mass spectrometer controlled by Xcalibur 2.2 software (Thermo Fisher Scientific, Waltham, MA). LC-MS was run with a modified ion pairing reversed-phase (RP) negative-ion electrospray ionization method. A total volume of 10 µl of sample is injected and separated on a Hydro-RP C18 column (100 by 2.1 mm, 2.5 µm particle size; Phenomenex, Torrance, CA) using a water/methanol gradient with tributylamine and acetic acid added to the aqueous mobile phase to enhance separation. The HPLC column is maintained at flow rate of 200 µl/min with the temperature of 30°C. The solvents and gradient are as follows: solvent A is 3% aqueous methanol with 10 mM tributylamine and 15 mM acetic acid, and solvent B is 100% methanol. The gradient is 0 min, 0% B; 5 min, 20% B; 7.5 min, 20% B; 13 min, 55% B; 15.5 min, 95% B; 18.5 min, 95% B; 19 min, 0% B; and 25 min, 0% B. The Exactive Plus is operated in negative-ion mode at maximum resolving power (140,000) and scans from m/z 72 to 1,000 for the first 90 s and then from m/z 85 to 1,000 for the remainder of the chromatographic run for the detection of small-molecule metabolites. **Table S1**. Log2 fold changes for lipids (positive mode) from isolated cecal bacteria with POPs exposure for 4 h relative to vehicle.

| **Lipids** | **TCDF**  **0.6 µM** | **TCDF**  **6 µM** | **TCDD**  **0.06 µM** | **TCDF**  **0.6 µM** | **PCB 126**  **0.6 µM** | **PCB 126**  **6 µM** | **PCB 153**  **0.6 µM** | **PCB 153**  **6 µM** |
| --- | --- | --- | --- | --- | --- | --- | --- | --- |
| ACar 18:1; [M]+ | -0.60 | -1.15 | -0.74 | -0.58 | -1.02 | -0.20 | -0.30 | -0.46 |
| w/o MS2:ACar 15:3; [M]+ | 0.03 | -0.13 | 0.56 | 0.49 | 0.35 | 0.28 | 0.60 | 0.50 |
| w/o MS2:ACar 21:1; [M]+ | 0.57 | 0.19 | 0.72 | 0.28 | 0.59 | 0.50 | 0.88 | 0.88 |
| w/o MS2:ACar 21:3; [M]+ | 0.67 | 0.92 | 0.84 | 0.96 | 1.11 | 0.93 | 1.23 | 1.16 |
| w/o MS2:ACar 21:5; [M]+ | 0.16 | -0.75 | -0.41 | 0.06 | -0.60 | -0.22 | -0.26 | 0.26 |
| w/o MS2:ACar 22:1; [M]+ | 0.22 | 0.02 | 0.24 | 0.32 | 0.51 | 0.30 | 0.55 | 0.51 |
| w/o MS2:ACar 22:5; [M]+ | -0.15 | -0.12 | 0.08 | 0.29 | 0.39 | 0.75 | 0.48 | 0.65 |
| w/o MS2:ACar 23:1; [M]+ | 0.25 | 0.05 | 0.61 | 0.37 | 0.68 | 0.17 | 1.04 | 0.72 |
| w/o MS2:ACar 25:1; [M]+ | 0.40 | 0.12 | 0.67 | 0.17 | 0.71 | 0.47 | 0.88 | 0.66 |
| w/o MS2:ACar 6:1; [M]+ | 0.31 | 0.17 | 0.74 | 0.62 | 0.74 | 0.56 | 0.82 | 0.95 |
| w/o MS2:ACar 8:2; [M]+ | 0.12 | -0.13 | 0.43 | 0.41 | 0.21 | 0.05 | 0.47 | 0.40 |
| w/o MS2:ACar 26:0; [M]+ | 0.42 | 0.61 | 0.46 | 0.45 | -0.09 | 0.02 | 0.38 | 0.58 |
| DAG 34:1; DAG 16:0-18:1; [M+NH4]+ | -0.07 | -1.22 | -0.81 | 0.29 | -0.89 | -0.49 | -0.45 | -0.53 |
| w/o MS2:DAG 21:5e; DAG 18:5e/3:0; [M+NH4]+ | 0.02 | -0.21 | 0.40 | 0.45 | 0.21 | 0.24 | 0.53 | 0.40 |
| w/o MS2:DAG 25:1e; DAG 8:0e/17:1; [M+NH4]+ | 0.27 | 0.68 | 0.87 | 0.68 | 0.39 | 0.27 | 0.75 | 0.96 |
| w/o MS2:DAG 25:3; DAG 9:0-16:3; [M+NH4]+ | 0.14 | 0.01 | 0.47 | 0.20 | 0.30 | 0.37 | 0.50 | 0.42 |
| w/o MS2:DAG 27:1e; DAG 8:0e/19:1; [M+NH4]+ | 0.25 | 0.43 | 0.93 | 0.54 | 0.37 | 0.49 | 0.67 | 0.90 |
| w/o MS2:DAG 29:1e; DAG 8:0e/21:1; [M+NH4]+ | 0.30 | 0.68 | 0.44 | 0.38 | 0.24 | 0.11 | 0.72 | 0.87 |
| w/o MS2:DAG 30:1e; DAG 28:1e/2:0; [M+NH4]+ | 0.32 | 0.32 | 0.26 | 0.36 | -0.21 | -0.28 | 0.48 | 0.52 |
| w/o MS2:DAG 30:2; DAG 8:0-22:2; [M+NH4]+ | 0.40 | 0.67 | 0.51 | 0.41 | 0.34 | 0.17 | 0.70 | 0.76 |
| w/o MS2:DAG 31:1e; DAG 9:0e/22:1; [M+NH4]+ | 0.11 | 0.33 | 0.16 | 0.16 | -0.27 | -0.25 | 0.28 | 0.23 |
| w/o MS2:DAG 31:4e; DAG 9:0e/22:4; [M+NH4]+ | 0.02 | 0.10 | 0.10 | 0.19 | -0.42 | -0.16 | -0.19 | 0.18 |
| w/o MS2:DAG 32:1e; DAG 8:0e/24:1; [M+NH4]+ | -0.36 | -1.26 | -0.99 | -0.54 | -0.97 | -0.76 | -0.78 | -0.69 |
| w/o MS2:DAG 32:2e; DAG 8:0e/24:2; [M+NH4]+ | 0.10 | 0.12 | 0.41 | 0.47 | 0.14 | -0.40 | 0.48 | 0.61 |
| w/o MS2:DAG 33:3e; DAG 9:0e/24:3; [M+NH4]+ | -1.30 | -5.24 | -2.27 | -1.86 | -2.09 | 0.47 | -2.48 | -0.76 |
| w/o MS2:DAG 34:1e; DAG 8:0e/26:1; [M+NH4]+ | -0.36 | -0.74 | -0.75 | -0.31 | -0.51 | -0.44 | -0.47 | -0.69 |
| w/o MS2:DAG 34:2e; DAG 18:2e/16:0; [M+NH4]+ | -0.56 | -3.06 | -1.46 | -0.59 | -1.67 | -0.93 | -1.12 | -1.15 |
| w/o MS2:DAG 35:0; DAG 8:0-27:0; [M+NH4]+ | -0.16 | -0.58 | -0.33 | -0.06 | -0.40 | 0.20 | -0.36 | 0.40 |
| w/o MS2:DAG 35:1; DAG 9:0-26:1; [M+NH4]+ | 0.30 | 0.42 | 0.15 | 0.56 | 0.29 | 0.59 | 0.49 | 0.84 |
| w/o MS2:DAG 35:3e; DAG 9:0e/26:3; [M+NH4]+ | 0.04 | -0.14 | 0.19 | 0.33 | -0.12 | 0.13 | 0.23 | 0.49 |
| w/o MS2:DAG 35:7; DAG 9:0-26:7; [M+NH4]+ | 0.19 | -0.09 | 0.42 | 0.36 | -0.01 | 0.03 | 0.40 | 0.31 |
| w/o MS2:DAG 36:0; DAG 8:0-28:0; [M+NH4]+ | -0.26 | -1.47 | -1.18 | -0.67 | -0.87 | -0.33 | -0.55 | -0.98 |
| w/o MS2:DAG 36:2; DAG 8:0-28:2; [M+NH4]+ | 0.76 | 0.90 | 1.05 | 0.79 | 0.74 | 0.44 | 0.96 | 0.97 |
| w/o MS2:DAG 36:3; DAG 8:0-28:3; [M+NH4]+ | 0.47 | 0.27 | 0.35 | 0.89 | 0.47 | 0.39 | 0.74 | 0.70 |
| w/o MS2:DAG 36:5e; DAG 18:0e/18:5; [M+NH4]+ | -0.82 | -2.31 | -1.98 | -1.10 | -1.35 | -0.60 | -1.38 | -1.59 |
| w/o MS2:DAG 36:6e; DAG 8:0e/28:6; [M+NH4]+ | -0.42 | -2.16 | -2.03 | -0.76 | -1.04 | -0.07 | -1.08 | -1.26 |
| w/o MS2:DAG 36:8; DAG 18:3-18:5; [M+NH4]+ | 0.19 | 0.04 | 0.06 | 0.50 | 0.16 | -0.19 | 0.66 | 0.58 |
| w/o MS2:DAG 37:0; DAG 8:0-29:0; [M+NH4]+ | 0.12 | -0.12 | 0.13 | 0.36 | 0.30 | 0.42 | 0.07 | 0.57 |
| w/o MS2:DAG 37:1; DAG 9:0-28:1; [M+NH4]+ | 0.32 | 0.70 | 0.84 | 0.30 | 0.59 | 0.47 | 0.91 | 0.70 |
| w/o MS2:DAG 37:1e; DAG 9:0e/28:1; [M+NH4]+ | -0.42 | -0.37 | -0.49 | -0.39 | -0.23 | -0.16 | -0.45 | -0.06 |
| w/o MS2:DAG 37:2e; DAG 9:0e/28:2; [M+NH4]+ | -0.03 | -0.51 | -0.09 | 0.25 | -0.42 | 0.39 | 0.18 | 0.51 |
| w/o MS2:DAG 37:3e; DAG 9:0e/28:3; [M+NH4]+ | 0.24 | -0.46 | 0.33 | 0.57 | 0.36 | 0.20 | 0.28 | 0.60 |
| w/o MS2:DAG 37:7e; DAG 9:0e/28:7; [M+NH4]+ | -0.87 | -3.03 | -1.87 | -1.90 | -1.50 | 0.13 | -2.27 | -1.75 |
| w/o MS2:DAG 38:2e; DAG 10:0e/28:2; [M+NH4]+ | -0.17 | -0.71 | -0.19 | -0.23 | -0.68 | 0.11 | -0.24 | -0.08 |
| w/o MS2:DAG 38:4; DAG 8:0-30:4; [M+NH4]+ | 0.10 | -0.28 | 0.51 | 0.37 | 0.12 | 0.20 | 0.54 | 0.79 |
| w/o MS2:DAG 38:7e; DAG 10:0e/28:7; [M+NH4]+ | -1.20 | -2.99 | -2.44 | -1.57 | -1.88 | -0.88 | -1.87 | -2.08 |
| w/o MS2:DAG 38:8; DAG 8:0-30:8; [M+NH4]+ | 0.19 | 0.19 | 0.49 | 0.48 | 0.38 | 0.09 | 0.63 | 0.62 |
| w/o MS2:DAG 39:1e; DAG 11:0e/28:1; [M+NH4]+ | 0.49 | 0.20 | 0.05 | 0.38 | 0.68 | 0.75 | 0.23 | 0.71 |
| w/o MS2:DAG 39:2; DAG 9:0-30:2; [M+NH4]+ | -0.07 | -0.20 | -0.42 | -0.06 | 0.11 | 0.03 | -0.07 | 0.37 |
| w/o MS2:DAG 39:4; DAG 9:0-30:4; [M+NH4]+ | -0.45 | -0.93 | -0.87 | -0.48 | -0.63 | -0.05 | -0.41 | -0.62 |
| w/o MS2:DAG 39:5; DAG 21:0-18:5; [M+NH4]+ | -0.19 | -0.97 | -0.69 | -0.49 | -0.77 | 0.22 | -0.54 | -0.48 |
| w/o MS2:DAG 39:6; DAG 9:0-30:6; [M+NH4]+ | -0.60 | -0.77 | -0.58 | -0.39 | -0.59 | 0.24 | -0.43 | -0.38 |
| w/o MS2:DAG 39:7; DAG 9:0-30:7; [M+NH4]+ | 0.14 | 0.22 | 0.08 | 0.57 | 0.50 | 0.14 | 0.66 | 0.61 |
| w/o MS2:DAG 40:0; DAG 8:0-32:0; [M+NH4]+ | 0.38 | 0.15 | -0.27 | -0.07 | 0.43 | 0.33 | 0.14 | 0.72 |
| w/o MS2:DAG 41:2; DAG 9:0-32:2; [M+NH4]+ | 0.93 | 0.94 | 0.78 | 0.97 | 0.92 | 0.92 | 0.62 | 1.17 |
| w/o MS2:DAG 41:3e; DAG 13:0e/28:3; [M+NH4]+ | 0.28 | 0.16 | 0.13 | 0.03 | 0.67 | 0.19 | -0.03 | 0.61 |
| w/o MS2:DAG 43:1; DAG 9:0-34:1; [M+NH4]+ | 0.18 | 0.19 | 0.35 | 0.63 | 0.05 | 0.43 | 0.23 | 1.45 |
| w/o MS2:DAG 45:1; DAG 9:0-36:1; [M+NH4]+ | -0.01 | -0.39 | -0.24 | 0.06 | -0.13 | -0.03 | 0.08 | 0.83 |
| w/o MS2:DAG 45:9; DAG 9:0-36:9; [M+NH4]+ | -0.22 | -0.78 | -0.06 | 0.09 | -0.01 | 0.30 | -0.13 | 0.34 |
| w/o MS2:DAG 46:2; DAG 14:1-32:1; [M+NH4]+ | 1.93 | 0.02 | 0.68 | 1.16 | -0.13 | -0.08 | 0.62 | 2.66 |
| w/o MS2:DAG 46:4e; DAG 18:0e/28:4; [M+NH4]+ | 0.17 | -0.11 | -0.23 | 0.14 | -0.06 | -0.17 | 0.15 | 0.38 |
| w/o MS2:DAG 49:3e; DAG 21:0e/28:3; [M+NH4]+ | 0.21 | 0.87 | 1.02 | 0.57 | 0.65 | 0.59 | 0.82 | 1.06 |
| w/o MS2:DAG 49:3e; DAG 21:1e/28:2; [M+NH4]+ | 0.51 | 1.14 | 1.26 | 0.72 | 0.41 | 0.13 | 1.03 | 0.76 |
| w/o MS2:DAG 50:12; DAG 8:0-42:12; [M+NH4]+ | 0.20 | 0.03 | 0.37 | 0.41 | 0.63 | 0.55 | 0.67 | 1.00 |
| w/o MS2:DAG 50:4e; DAG 22:1e/28:3; [M+NH4]+ | 0.19 | 0.80 | 0.65 | 0.05 | 0.02 | -0.14 | 0.26 | 0.33 |
| w/o MS2:DAG 54:14e; DAG 26:7e/28:7; [M+NH4]+ | -0.01 | -0.75 | -0.61 | -0.35 | 0.22 | 0.36 | -0.75 | 0.42 |
| w/o MS2:DAG 20:2e; DAG 16:2e/4:0; [M+NH4]+ | -0.47 | -1.44 | -0.96 | -0.69 | -1.34 | -0.03 | -0.24 | -0.31 |
| w/o MS2:DAG 29:1e; DAG 8:0e/21:1; [M+NH4]+ | 0.30 | 0.49 | 0.13 | 0.22 | -0.05 | -0.25 | 0.55 | 0.39 |
| w/o MS2:DAG 35:3e; DAG 9:0e/26:3; [M+NH4]+ | -0.91 | -2.51 | -0.80 | -0.53 | -0.67 | 0.21 | -1.05 | -0.34 |
| w/o MS2:DAG 36:0; DAG 8:0-28:0; [M+NH4]+ | -0.03 | -0.72 | -0.10 | 0.02 | -0.53 | 0.27 | -0.02 | 0.26 |
| w/o MS2:DAG 48:1; DAG 8:0-40:1; [M+NH4]+ | -0.49 | -1.03 | -1.20 | -0.54 | -1.00 | -0.62 | -0.67 | -0.08 |
| LPC 16:0e; [M+H]+ | -0.61 | -1.72 | -1.22 | -1.07 | -0.87 | 0.09 | -0.83 | 0.01 |
| LPC 16:0-SN1; [M+H]+ | -0.69 | -1.63 | -1.26 | 0.43 | -0.44 | 0.29 | -0.73 | 0.24 |
| LPC 18:0; [M+H]+ | -0.40 | -1.07 | -0.31 | 0.00 | -0.49 | 0.37 | -0.50 | 0.27 |
| LPC 18:0e; [M+H]+ | -0.34 | -1.38 | -0.89 | -0.61 | -0.73 | -0.19 | -0.74 | -0.09 |
| LPC 18:1; [M+H]+ | -0.90 | -2.33 | -1.23 | -0.45 | -0.59 | 0.56 | -0.63 | 0.03 |
| LPC 18:1-SN1; [M+H]+ | -0.76 | -1.64 | -1.11 | 0.39 | -0.67 | -0.01 | -1.01 | -0.19 |
| LPC 20:0-SN1; [M+H]+ | -0.38 | -1.03 | -0.51 | -0.31 | -0.65 | 0.20 | -0.40 | 0.18 |
| LPC 20:1-SN1; [M+H]+ | -1.01 | -2.10 | -2.30 | -1.43 | -1.24 | -0.36 | -1.57 | -0.70 |
| LPC 24:0-SN1; [M+H]+ | 0.13 | -0.19 | 0.32 | 0.13 | -0.18 | 0.29 | 0.31 | 0.66 |
| LPC 26:1-SN1; [M+H]+ | 0.31 | 0.58 | 0.97 | 1.12 | 0.58 | 0.90 | 0.88 | 1.17 |
| w/o MS2:LPC 15:0; [M+Na]+ | -0.65 | -1.95 | -1.46 | -0.93 | -1.13 | -0.49 | -1.34 | -0.47 |
| w/o MS2:LPC 18:1e; [M+H]+ | -0.37 | -1.50 | -0.53 | -0.10 | -0.64 | 0.57 | -0.37 | 0.20 |
| w/o MS2:LPC 18:4; [M+Na]+ | 0.16 | 0.06 | 0.46 | 0.47 | 0.07 | 0.45 | 0.55 | 0.40 |
| w/o MS2:LPC 22:2e; [M+H]+ | -0.48 | -1.04 | -0.48 | -0.58 | -0.67 | 0.25 | -0.59 | -0.66 |
| w/o MS2:LPC 24:2e; [M+H]+ | -0.60 | -0.61 | 0.01 | -0.11 | -0.15 | 0.79 | -0.12 | 0.52 |
| w/o MS2:LPC 34:3; [M+Na]+ | 0.20 | -0.11 | 0.16 | 0.92 | 0.66 | -0.10 | -0.04 | 0.95 |
| PC 33:2; [M+H]+ | 0.27 | -0.04 | 0.39 | 0.97 | 0.43 | 0.64 | 0.52 | 0.81 |
| PC 34:1; [M+H]+ | 0.47 | -0.58 | 0.67 | 1.04 | -0.02 | 0.71 | 0.79 | 1.11 |
| PC 34:2; [M+H]+ | 0.29 | 0.00 | 0.38 | 0.77 | 0.35 | 0.04 | 0.32 | 0.77 |
| PC 36:3e; [M+H]+ | 0.50 | -0.16 | 0.75 | 1.34 | 0.76 | 1.27 | 0.88 | 1.34 |
| PC 38:1e; [M+H]+ | 0.07 | -0.29 | 0.20 | 0.43 | 0.67 | 0.50 | 0.30 | 0.79 |
| PC 38:4; [M+H]+ | 0.17 | -0.72 | -0.25 | 0.69 | 0.53 | 0.66 | 0.58 | 0.29 |
| PC 38:6; [M+H]+ | 0.39 | 0.15 | 0.27 | 0.83 | 0.18 | 0.57 | 0.53 | 0.85 |
| w/o MS2:PC 36:9; PC 18:4-18:5; [M+H]+ | 0.71 | 0.09 | 0.92 | 0.91 | 1.15 | 0.53 | 1.00 | 1.28 |
| PE 28:0; [M+H]+ | 0.30 | 0.50 | 0.66 | -0.23 | 0.07 | 0.41 | 0.62 | 0.51 |
| PE 31:0; [M+H]+ | 0.27 | -0.39 | -0.05 | 0.32 | 0.03 | 0.37 | 0.42 | 0.85 |
| w/o MS2:PE 23:1e; PE 21:1e/2:0; [M+H]+ | 0.22 | -0.28 | 0.42 | 0.36 | -0.01 | 0.85 | 0.33 | 0.69 |
| w/o MS2:PE 27:0; [M+Na]+ | 0.35 | 0.63 | 0.40 | 0.04 | -0.13 | -0.28 | 0.33 | 0.03 |
| w/o MS2:PE 30:0; PE 8:0-22:0; [M+H]+ | 0.43 | 0.99 | 1.03 | 1.04 | 0.66 | 0.82 | 1.08 | 0.60 |
| w/o MS2:PE 30:1; PE 8:0-22:1; [M+H]+ | 0.33 | 0.06 | 0.40 | 0.55 | -0.39 | -0.19 | 0.56 | 0.45 |
| w/o MS2:PE 33:0; PE 8:0-25:0; [M+H]+ | 0.54 | 0.00 | -0.42 | 0.57 | 0.62 | 0.51 | 0.45 | 0.96 |
| w/o MS2:PE 33:1; [M+Na]+ | -0.22 | -0.19 | -0.13 | -0.23 | -0.43 | -0.17 | -0.58 | -0.31 |
| w/o MS2:PE 33:2; [M+Na]+ | -0.38 | -0.44 | -0.33 | -0.68 | -0.47 | -0.17 | -0.50 | -0.44 |
| w/o MS2:PE 33:2e; PE 9:0e/24:2; [M+H]+ | 0.80 | 0.69 | 1.02 | 0.98 | 0.81 | 1.00 | 1.29 | 0.89 |
| w/o MS2:PE 34:1; PE 8:0-26:1; [M+H]+ | 0.22 | -0.41 | 0.45 | 0.98 | 0.15 | 1.00 | 0.20 | 1.05 |
| w/o MS2:PE 35:1; PE 9:0-26:1; [M+H]+ | 0.55 | -0.23 | 0.47 | 1.00 | 0.28 | 0.72 | 0.56 | 1.06 |
| w/o MS2:PE 35:2; PE 9:0-26:2; [M+H]+ | 0.74 | -0.12 | 0.70 | 1.24 | 0.33 | 0.71 | 0.55 | 1.15 |
| w/o MS2:PE 35:4; [M+Na]+ | -0.51 | -0.62 | -0.47 | -1.04 | -0.92 | -0.56 | -0.87 | -1.05 |
| w/o MS2:PE 35:4; PE 9:0-26:4; [M+H]+ | -0.21 | -0.27 | -0.30 | -0.90 | -0.54 | -0.73 | -0.56 | -0.47 |
| w/o MS2:PE 35:5; [M+Na]+ | -0.27 | -0.50 | -0.08 | -0.48 | -0.61 | -0.39 | -0.49 | -0.68 |
| w/o MS2:PE 36:1; [M+Na]+ | 0.00 | -0.65 | -0.18 | 0.41 | -0.07 | -0.58 | -0.07 | 0.38 |
| w/o MS2:PE 36:1; PE 8:0-28:1; [M+H]+ | 0.52 | -0.03 | 0.53 | 1.12 | 0.32 | 0.80 | 0.66 | 0.90 |
| w/o MS2:PE 36:6; [M+Na]+ | 0.45 | 0.04 | 0.72 | 0.86 | 0.63 | 0.35 | 0.34 | 0.46 |
| w/o MS2:PE 37:1; PE 9:0-28:1; [M+H]+ | 2.69 | 2.36 | 0.67 | 3.23 | 1.51 | 1.48 | 1.83 | 2.16 |
| w/o MS2:PE 37:3; PE 9:0-28:3; [M+H]+ | 0.69 | 0.28 | 0.40 | 0.94 | 0.66 | 0.72 | 0.74 | 1.04 |
| w/o MS2:PE 37:4; PE 9:0-28:4; [M+H]+ | 0.43 | 0.38 | 0.33 | 0.86 | 0.33 | 0.32 | 0.51 | 0.68 |
| w/o MS2:PE 38:0; PE 8:0-30:0; [M+H]+ | 0.59 | 0.41 | 0.83 | 0.76 | -0.10 | 0.00 | 0.91 | 0.72 |
| w/o MS2:PE 38:1; PE 8:0-30:1; [M+H]+ | 0.54 | -0.24 | 0.37 | 1.16 | 0.26 | 0.86 | 0.60 | 1.04 |
| w/o MS2:PE 38:2; PE 8:0-30:2; [M+H]+ | 0.46 | -0.24 | 0.20 | 0.92 | -0.29 | 0.53 | 0.53 | 0.88 |
| w/o MS2:PE 38:3; PE 8:0-30:3; [M+H]+ | 0.21 | 0.01 | 0.06 | 0.04 | 0.27 | 0.14 | 0.23 | 0.53 |
| w/o MS2:PE 38:6; PE 8:0-30:6; [M+H]+ | 0.46 | 0.28 | 0.64 | 0.88 | 0.28 | 0.66 | 0.55 | 0.83 |
| w/o MS2:PE 39:1; PE 9:0-30:1; [M+H]+ | 0.45 | -0.46 | 0.38 | 0.89 | 0.01 | 0.59 | 0.51 | 0.90 |
| w/o MS2:PE 39:1e; PE 11:0e/28:1; [M+H]+ | -0.18 | -0.17 | 0.36 | 0.36 | 0.37 | 0.49 | 0.38 | 0.73 |
| w/o MS2:PE 39:2; PE 9:0-30:2; [M+H]+ | 0.37 | -0.03 | -0.10 | 0.61 | 0.37 | 0.07 | 0.31 | 0.74 |
| w/o MS2:PE 39:2e; PE 11:0e/28:2; [M+H]+ | 0.47 | -0.25 | 0.29 | 0.78 | 0.49 | 0.92 | 0.62 | 0.84 |
| w/o MS2:PE 39:3; PE 9:0-30:3; [M+H]+ | 0.23 | -0.21 | 0.13 | 0.71 | 0.36 | 0.32 | -0.06 | 0.52 |
| w/o MS2:PE 39:3e; PE 11:0e/28:3; [M+H]+ | -0.68 | -0.43 | 0.29 | 0.89 | 0.04 | 0.45 | 0.17 | 0.87 |
| w/o MS2:PE 39:4; [M+Na]+ | 0.23 | -0.19 | 0.16 | 0.70 | 0.59 | 0.63 | 0.16 | 0.65 |
| w/o MS2:PE 39:4; PE 9:0-30:4; [M+H]+ | 0.36 | -0.06 | -0.14 | 0.80 | 0.46 | 0.49 | 0.51 | 0.68 |
| w/o MS2:PE 39:5; PE 21:0-18:5; [M+H]+ | 0.35 | -0.54 | 0.08 | 0.84 | 0.11 | 0.32 | 0.24 | 0.70 |
| w/o MS2:PE 39:6; PE 9:0-30:6; [M+H]+ | 0.93 | -0.30 | 0.79 | 1.36 | 0.71 | 0.99 | 0.87 | 1.52 |
| w/o MS2:PE 39:6e; PE 11:0e/28:6; [M+H]+ | 0.39 | -0.24 | 0.41 | 0.95 | 0.37 | 0.90 | 0.57 | 0.86 |
| w/o MS2:PE 40:6; PE 8:0-32:6; [M+H]+ | -0.25 | -0.64 | -0.26 | -0.20 | -0.13 | -0.33 | -0.16 | 0.04 |
| w/o MS2:PE 41:1; PE 9:0-32:1; [M+H]+ | 0.14 | -0.44 | -0.11 | 0.51 | 0.25 | 0.38 | -0.01 | 0.69 |
| w/o MS2:PE 41:3; PE 9:0-32:3; [M+H]+ | 0.41 | -0.05 | 0.04 | 0.46 | 0.55 | 0.80 | 0.39 | 0.81 |
| w/o MS2:PE 41:3e; PE 25:0e/16:3; [M+H]+ | 0.46 | -0.28 | 0.49 | 0.21 | 0.70 | 1.20 | 0.82 | 1.04 |
| w/o MS2:PE 41:4; [M+Na]+ | -0.62 | -0.54 | -0.04 | 0.53 | 0.26 | 0.26 | -0.09 | 0.32 |
| w/o MS2:PE 41:4e; PE 13:0e/28:4; [M+H]+ | 0.26 | -0.50 | 0.23 | 0.55 | 0.05 | 0.74 | 0.41 | 0.73 |
| w/o MS2:PE 41:6; [M+Na]+ | 0.56 | 0.38 | 0.43 | 0.56 | 0.18 | 0.32 | 0.33 | 0.50 |
| w/o MS2:PE 41:6e; PE 13:0e/28:6; [M+H]+ | 0.06 | -1.18 | 0.65 | 0.93 | 0.69 | 0.87 | 0.43 | 0.60 |
| w/o MS2:PE 43:1; PE 9:0-34:1; [M+H]+ | -0.11 | 0.02 | 0.34 | 0.58 | 0.37 | 0.19 | 0.39 | 0.80 |
| w/o MS2:PE 43:2; PE 9:0-34:2; [M+H]+ | 0.74 | 0.54 | 0.80 | 1.31 | 0.83 | 1.13 | 0.77 | 0.73 |
| w/o MS2:PE 43:7e; PE 15:0e/28:7; [M+H]+ | 0.18 | -0.42 | -0.14 | 0.23 | 0.28 | 0.67 | -0.21 | 0.32 |
| w/o MS2:PE 45:1; PE 9:0-36:1; [M+H]+ | 0.44 | -0.30 | 0.31 | 0.73 | 0.48 | 0.75 | 0.65 | 0.97 |
| w/o MS2:PE 45:4e; PE 17:0e/28:4; [M+H]+ | -0.26 | -0.66 | -0.51 | -0.08 | -0.04 | 0.26 | -0.10 | 0.34 |
| w/o MS2:PE 47:4; PE 9:0-38:4; [M+H]+ | 0.30 | 0.03 | 0.23 | 0.46 | 0.62 | 0.66 | 0.35 | 0.81 |
| w/o MS2:PE 47:5e; PE 19:0e/28:5; [M+H]+ | 0.61 | 0.00 | 0.16 | 0.18 | 0.91 | 0.86 | 0.05 | 0.97 |
| w/o MS2:PE 48:9e; PE 20:2e/28:7; [M+H]+ | 0.56 | 0.99 | 1.11 | 1.06 | 0.45 | 0.27 | 0.78 | 0.96 |
| TAG 44:0; TAG 14:0-14:0-16:0; [M+NH4]+ | -0.83 | -0.63 | -0.82 | -0.75 | -0.84 | -0.25 | -0.64 | -0.80 |
| TAG 49:0; TAG 15:0-17:0-17:0; [M+NH4]+ | -0.80 | -2.46 | -1.61 | -1.16 | -1.43 | -0.43 | -1.63 | -1.17 |
| w/o MS2:TAG 37:3; TAG 8:0-9:0-20:3; [M+NH4]+ | -1.40 | -2.08 | -1.35 | -1.50 | -1.54 | -0.09 | -1.51 | -1.40 |
| w/o MS2:TAG 38:6; TAG 8:0-8:0-22:6; [M+Na]+ | 0.23 | 0.77 | 0.67 | 0.23 | 0.00 | 0.43 | 0.92 | 0.50 |
| w/o MS2:TAG 39:0; TAG 8:0-8:0-23:0; [M+NH4]+ | 0.58 | -0.03 | 0.89 | 1.02 | 0.57 | 0.77 | 0.90 | 1.04 |
| w/o MS2:TAG 39:3; TAG 13:1-13:1-13:1; [M+Na]+ | -0.38 | -0.57 | -0.14 | -0.33 | -0.23 | -0.15 | -0.29 | -0.28 |
| w/o MS2:TAG 39:5; TAG 8:0-13:0-18:5; [M+NH4]+ | -0.27 | -1.18 | -1.17 | -0.63 | -1.03 | -0.35 | -0.76 | -0.53 |
| w/o MS2:TAG 39:6; TAG 8:0-9:0-22:6; [M+Na]+ | 0.31 | 0.03 | 0.60 | 0.51 | 0.04 | 0.32 | 0.69 | 0.51 |
| w/o MS2:TAG 40:0; TAG 8:0-8:0-24:0; [M+NH4]+ | 0.06 | -0.70 | 0.16 | -0.12 | 0.18 | -0.13 | -0.07 | 0.13 |
| w/o MS2:TAG 40:5; TAG 8:0-14:0-18:5; [M+Na]+ | -0.12 | 0.14 | -0.13 | -0.18 | 0.03 | 0.07 | 0.24 | 0.06 |
| w/o MS2:TAG 40:6; TAG 8:0-8:0-24:6; [M+Na]+ | -0.67 | -0.37 | -0.22 | -0.52 | -0.78 | 0.18 | 0.01 | -1.01 |
| w/o MS2:TAG 41:1; TAG 8:0-9:0-24:1; [M+NH4]+ | 0.93 | 0.93 | 1.23 | 0.72 | 1.91 | 1.70 | 1.30 | 1.58 |
| w/o MS2:TAG 41:5; TAG 8:0-13:1-20:4; [M+Na]+ | 0.32 | -0.31 | 0.53 | 0.33 | 0.05 | 0.26 | 0.55 | 0.64 |
| w/o MS2:TAG 42:4; TAG 8:0-8:0-26:4; [M+Na]+ | 0.23 | 0.40 | 0.19 | 0.15 | 0.20 | -0.20 | 0.22 | -0.20 |
| w/o MS2:TAG 42:6; TAG 8:0-8:0-26:6; [M+Na]+ | 0.20 | 0.53 | 0.57 | 0.44 | 0.29 | 0.64 | 0.42 | 0.50 |
| w/o MS2:TAG 43:0; TAG 8:0-8:0-27:0; [M+NH4]+ | 0.11 | -0.28 | 0.05 | -0.16 | 0.16 | 0.11 | -0.09 | 0.27 |
| w/o MS2:TAG 44:10; TAG 8:0-18:5-18:5; [M+NH4]+ | 0.33 | -0.03 | 0.25 | -0.07 | -0.29 | 0.04 | 0.00 | 0.41 |
| w/o MS2:TAG 44:7; TAG 8:0-8:0-28:7; [M+Na]+ | -0.22 | 0.01 | 0.38 | 0.30 | -0.35 | 0.04 | 0.14 | 0.59 |
| w/o MS2:TAG 48:0; TAG 8:0-8:0-32:0; [M+Na]+ | 0.00 | -0.78 | -0.84 | 0.23 | -0.97 | -0.68 | -1.08 | -0.82 |
| w/o MS2:TAG 49:1; TAG 8:0-9:0-32:1; [M+NH4]+ | -0.47 | -2.13 | -0.81 | -0.75 | -0.71 | 0.28 | -1.05 | -0.46 |
| w/o MS2:TAG 50:0; TAG 8:0-8:0-34:0; [M+Na]+ | 0.61 | 1.11 | 0.64 | 2.05 | 1.20 | 1.51 | 0.55 | 0.87 |
| w/o MS2:TAG 50:3; TAG 8:0-8:0-34:3; [M+NH4]+ | -0.45 | -1.24 | -0.71 | -0.52 | -0.41 | -0.64 | -0.84 | -0.84 |
| w/o MS2:TAG 51:11; TAG 9:0-18:5-24:6; [M+NH4]+ | 0.32 | -0.16 | 0.74 | 1.51 | 0.62 | 1.04 | 1.49 | 1.27 |
| w/o MS2:TAG 51:2; TAG 8:0-9:0-34:2; [M+NH4]+ | -0.83 | -1.59 | -0.21 | -0.87 | -0.51 | 0.85 | -0.57 | -0.15 |
| w/o MS2:TAG 51:3; TAG 8:0-9:0-34:3; [M+NH4]+ | -1.17 | -1.85 | -1.44 | -0.87 | -0.86 | -1.25 | -1.10 | -0.95 |
| w/o MS2:TAG 52:3; TAG 8:0-8:0-36:3; [M+NH4]+ | -0.39 | -1.37 | -0.82 | 0.08 | -0.38 | -0.92 | -0.33 | -0.06 |
| w/o MS2:TAG 53:1; TAG 8:0-9:0-36:1; [M+NH4]+ | 0.33 | -0.15 | 0.12 | 0.94 | -0.19 | 0.37 | 0.33 | 0.48 |
| w/o MS2:TAG 53:11; TAG 9:0-18:5-26:6; [M+NH4]+ | -0.43 | -0.60 | 0.36 | 1.00 | 0.42 | 0.74 | 0.84 | 0.94 |
| w/o MS2:TAG 53:2; TAG 8:0-9:0-36:2; [M+NH4]+ | -1.39 | -3.00 | -1.10 | -0.21 | -0.89 | 1.20 | -1.18 | -0.36 |
| w/o MS2:TAG 53:9; TAG 8:0-9:0-36:9; [M+Na]+ | -0.58 | -1.33 | -0.55 | -0.39 | -0.01 | -0.51 | -0.81 | -0.47 |
| w/o MS2:TAG 54:1; TAG 16:0-16:0-22:1; [M+NH4]+ | -0.43 | -0.29 | -0.18 | -1.18 | -0.14 | -0.45 | -0.28 | -0.17 |
| w/o MS2:TAG 55:0; TAG 8:0-9:0-38:0; [M+NH4]+ | 0.20 | 0.53 | 0.51 | 0.94 | -0.10 | 0.33 | 0.90 | 0.58 |
| w/o MS2:TAG 56:1; TAG 8:0-10:0-38:1; [M+NH4]+ | 0.60 | 0.10 | 0.32 | 0.75 | 0.03 | 0.24 | 0.42 | 0.38 |
| w/o MS2:TAG 56:11; TAG 14:1-16:3-26:7; [M+Na]+ | 0.32 | 0.21 | 0.79 | 1.17 | 0.35 | 0.67 | 1.05 | 1.29 |
| w/o MS2:TAG 56:12; TAG 8:0-18:5-30:7; [M+NH4]+ | -0.11 | 0.82 | 1.32 | 1.87 | 1.21 | 1.42 | 1.72 | 1.46 |
| w/o MS2:TAG 56:2; TAG 8:0-10:0-38:2; [M+NH4]+ | -1.09 | -0.31 | -0.50 | -2.27 | -0.55 | -0.87 | -0.61 | -1.30 |
| w/o MS2:TAG 59:0; TAG 8:0-13:0-38:0; [M+NH4]+ | 0.56 | 0.37 | 0.73 | 0.73 | 0.73 | -0.05 | 0.51 | 0.56 |
| w/o MS2:TAG 60:9; TAG 8:0-14:0-38:9; [M+Na]+ | 0.59 | 0.97 | 0.13 | 0.51 | 0.97 | 0.67 | 0.46 | 0.56 |
| w/o MS2:LPE 15:0e; [M+H]+ | 0.24 | -0.53 | -0.03 | 0.36 | -0.12 | 0.26 | -0.11 | 0.36 |
| w/o MS2:LPE 16:0e; [M+H]+ | -0.81 | -2.03 | -1.58 | -0.86 | -1.32 | -0.32 | -1.18 | -0.35 |
| w/o MS2:LPE 16:1e; [M+H]+ | -1.01 | -1.87 | -1.37 | -0.28 | -1.00 | 0.19 | -1.06 | -0.48 |
| w/o MS2:LPE 17:0e; [M+H]+ | 0.56 | 0.30 | 1.14 | 0.77 | 0.33 | 0.94 | 1.06 | 0.90 |
| w/o MS2:LPE 17:1e; [M+H]+ | 0.09 | 0.05 | 0.06 | 0.60 | 0.38 | 0.58 | 0.57 | 0.62 |
| w/o MS2:LPE 18:0e; [M+H]+ | -0.71 | -1.68 | -1.47 | -1.27 | -1.83 | -0.52 | -1.42 | -0.77 |
| w/o MS2:LPE 18:2e; [M+H]+ | -0.88 | -1.87 | -1.46 | -0.19 | -1.36 | -0.09 | -1.09 | -0.26 |
| w/o MS2:LPE 18:3e; [M+H]+ | -0.63 | -1.67 | -0.87 | 0.01 | -0.53 | 0.18 | -1.01 | -0.14 |
| w/o MS2:LPE 20:0e; [M+H]+ | -0.58 | -1.04 | -0.82 | -0.75 | -0.79 | -0.47 | -0.77 | -0.42 |
| w/o MS2:LPE 20:2e; [M+H]+ | -0.10 | -1.13 | -0.91 | -0.27 | -0.90 | 0.15 | -0.52 | -0.36 |
| w/o MS2:LPE 21:2e; [M+H]+ | -0.57 | -1.25 | -1.05 | -0.52 | -0.48 | 0.65 | -0.71 | 0.06 |
| w/o MS2:LPE 22:1; [M+H]+ | 0.13 | -0.49 | -0.22 | 0.19 | -0.19 | 0.05 | -0.18 | 0.46 |
| w/o MS2:LPE 22:2e; [M+H]+ | 0.40 | -0.23 | 0.27 | 0.43 | 0.32 | 1.14 | 0.44 | 0.94 |
| w/o MS2:LPE 22:6; [M+H]+ | -0.24 | -0.94 | -0.01 | 0.24 | -0.47 | 0.22 | -0.31 | 0.13 |
| w/o MS2:LPE 24:1e; [M+H]+ | 0.39 | -0.23 | 0.39 | 0.50 | 0.60 | 1.21 | 0.39 | 0.96 |
| w/o MS2:LPE 27:0; [M+H]+ | 0.05 | -0.37 | 0.03 | 0.24 | -0.13 | 0.42 | 0.01 | 0.62 |
| w/o MS2:LPE 32:1; [M+H]+ | 0.36 | 0.42 | 0.70 | 0.52 | 0.14 | 0.49 | 0.92 | 0.71 |
| w/o MS2:LPE 32:2; [M+H]+ | 0.29 | 0.12 | 0.27 | 0.36 | 0.32 | 0.20 | 0.75 | 0.58 |
| w/o MS2:LPE 33:0; [M+H]+ | -0.16 | -0.01 | 0.18 | 0.47 | 0.27 | 0.24 | 0.08 | 0.39 |
| w/o MS2:LPE 34:3; [M+H]+ | 0.49 | 0.34 | 0.47 | 0.71 | 0.47 | 0.33 | 0.88 | 0.91 |
| w/o MS2:LPE 35:0; [M+H]+ | 0.33 | 0.47 | 0.37 | 0.55 | 0.13 | -0.15 | 0.61 | 0.54 |
| w/o MS2:LPE 36:0; [M+H]+ | -0.18 | 0.07 | 0.18 | 0.16 | 0.22 | 0.32 | 0.47 | 0.41 |
| w/o MS2:LPE 36:2; [M+H]+ | 1.07 | 0.82 | 1.42 | 1.58 | 1.21 | 1.50 | 1.53 | 1.27 |
| w/o MS2:LPE 36:5; [M+H]+ | 1.39 | 1.01 | 1.53 | 2.08 | 1.67 | 1.91 | 1.72 | 1.65 |
| w/o MS2:LPE 36:6; [M+H]+ | 0.65 | -0.10 | 0.69 | 1.35 | 0.57 | 1.13 | 0.84 | 1.31 |
| w/o MS2:LPE 38:7; [M+H]+ | 0.59 | -0.04 | 0.42 | 1.11 | 0.43 | 0.71 | 0.69 | 0.96 |
| w/o MS2:LPE 40:5; [M+H]+ | 0.58 | 0.00 | 0.54 | 1.09 | 0.64 | 0.89 | 0.82 | 1.01 |
| w/o MS2:LPE 42:0; [M+H]+ | -0.08 | -0.21 | -0.50 | -0.14 | -0.42 | -0.57 | -0.29 | 0.15 |
| w/o MS2:LPE 42:6; [M+H]+ | 0.12 | -0.13 | 0.53 | 0.95 | 0.46 | 0.21 | 0.76 | 0.81 |
| w/o MS2:SM d19:0; [M+Na]+ | 0.21 | 0.64 | 0.05 | -0.19 | 0.18 | -0.05 | -0.02 | 0.24 |
| w/o MS2:SM d34:0; [M+Na]+ | -0.59 | -0.56 | -0.33 | -0.49 | -0.03 | -0.20 | -1.02 | -0.11 |
| w/o MS2:SM d34:1; [M+Na]+ | -0.25 | -0.55 | -0.38 | -0.33 | -0.08 | 0.14 | -0.35 | -0.46 |
| w/o MS2:SM d34:1; SM d32:1/2:0; [M+H]+ | -0.50 | -1.08 | -0.55 | -0.27 | -0.47 | -0.53 | -0.47 | 0.14 |
| w/o MS2:SM d36:1; SM d34:1/2:0; [M+H]+ | 0.79 | 0.23 | 0.64 | 0.98 | 0.69 | 0.88 | -0.05 | 0.44 |
| w/o MS2:SM d40:2; SM d8:1/32:1; [M+H]+ | 0.23 | -0.01 | 0.00 | 0.23 | -0.52 | -0.07 | -0.12 | 0.44 |
| w/o MS2:SM d41:1; [M+Na]+ | -0.37 | -0.13 | 0.68 | 0.74 | 0.11 | 0.25 | 0.54 | 0.87 |
| w/o MS2:SM d41:3; SM d9:1/32:2; [M+H]+ | 0.32 | 0.32 | 0.43 | 0.63 | 0.17 | 0.38 | 0.32 | 0.81 |
| w/o MS2:SM d42:3; SM d8:1/34:2; [M+H]+ | 0.19 | 0.09 | 0.25 | 0.19 | 0.10 | 0.16 | 0.24 | 0.62 |
| w/o MS2:SM d42:4; SM d8:1/34:3; [M+H]+ | 0.67 | 0.54 | 0.86 | 0.64 | 0.41 | 0.82 | 0.52 | 1.12 |
| w/o MS2:SM d43:3; SM d9:1/34:2; [M+H]+ | 0.47 | 0.61 | 0.71 | 0.82 | 0.08 | 0.44 | 0.18 | 0.77 |
| w/o MS2:SM d44:4; SM d8:1/36:3; [M+H]+ | 0.02 | 0.35 | 0.60 | 0.28 | 0.16 | 0.15 | 0.37 | 0.84 |
| w/o MS2:SM d46:12; [M+Na]+ | 0.02 | 0.19 | 0.52 | 0.56 | 0.02 | 0.56 | 0.59 | 0.81 |
| w/o MS2:SM t32:0; [M+H]+ | 0.18 | 0.21 | 0.48 | 0.44 | -0.15 | 0.36 | 0.42 | 0.70 |
| w/o MS2:SM t37:3; [M+H]+ | 0.23 | 0.30 | 0.41 | 0.57 | 0.03 | 0.33 | 0.43 | 0.67 |
| w/o MS2:PG 31:1; PG 5:0-26:1; [M+NH4]+ | 0.09 | -0.13 | 0.43 | 0.47 | 0.44 | 0.29 | 0.69 | 0.74 |
| w/o MS2:PG 33:4; PG 7:0-26:4; [M+NH4]+ | -0.71 | -0.79 | -1.28 | -1.42 | -1.65 | -0.25 | -1.29 | -0.71 |
| w/o MS2:PG 34:0; PG 7:0-27:0; [M+NH4]+ | 0.00 | -0.62 | -0.60 | -0.24 | -0.54 | -0.13 | 0.09 | -0.46 |
| w/o MS2:PG 36:3; PG 14:0-22:3; [M+NH4]+ | 0.54 | 0.15 | 1.29 | 1.91 | 1.24 | 0.96 | 1.30 | 1.57 |
| w/o MS2:PG 41:3; PG 25:0-16:3; [M+NH4]+ | 0.27 | -0.51 | 1.40 | 1.86 | 1.13 | 1.19 | 1.73 | 1.55 |
| w/o MS2:Phytosphingosine 18:0; [M+H]+ | -1.02 | -1.04 | -0.88 | -0.87 | -0.48 | -0.48 | -0.83 | -0.54 |
| w/o MS2:Phytosphingosine 19:0; [M+H]+ | 0.09 | -0.70 | -0.63 | 0.36 | -0.39 | -0.23 | 0.05 | -0.11 |
| w/o MS2:Sphinganine 15:0; [M+H]+ | 0.09 | 0.52 | 0.94 | 0.79 | 0.61 | 0.23 | 1.03 | 0.58 |
| w/o MS2:Sphingosine 14:1; [M+H]+ | 0.65 | 0.83 | 0.67 | 0.79 | 1.08 | 0.77 | 0.69 | 0.41 |
| w/o MS2:Sphingosine 20:1; [M+H]+ | 0.26 | 0.20 | 0.50 | 0.41 | -0.06 | 0.26 | 0.33 | 0.23 |
| w/o MS2:Sphingosine 22:1; [M+H]+ | 0.36 | 0.32 | 0.74 | 0.60 | 0.18 | 0.54 | 0.87 | 0.80 |
| w/o MS2:Sphingosine 23:1; [M+H]+ | 0.37 | 0.44 | 0.74 | 0.71 | 0.32 | 0.56 | 0.72 | 0.61 |
| w/o MS2:Sphingosine 24:1; [M+H]+ | 0.39 | 0.48 | 0.74 | 0.62 | 0.40 | 0.50 | 0.89 | 0.84 |
| w/o MS2:Sphingosine 25:1; [M+H]+ | 0.51 | 0.53 | 0.70 | 0.75 | 0.45 | 0.36 | 0.72 | 0.85 |
| w/o MS2:Sphingosine 26:1; [M+H]+ | 0.36 | 0.41 | 0.65 | 0.79 | 0.23 | 0.51 | 0.61 | 0.75 |
| w/o MS2:Sphingosine 28:1; [M+H]+ | 0.07 | 0.08 | 0.17 | 0.47 | 0.09 | 0.19 | 0.46 | 0.40 |
| w/o MS2:PI 31:1; [M+NH4]+ | 0.84 | 0.49 | 0.84 | 0.70 | 0.21 | 0.31 | 0.65 | 0.76 |
| w/o MS2:PI 32:2; [M+NH4]+ | 0.23 | -0.23 | 0.78 | 0.71 | 0.23 | 0.15 | 0.45 | 0.42 |
| w/o MS2:PI 37:2; [M+NH4]+ | -0.33 | -0.97 | -0.55 | -0.44 | -0.77 | -0.43 | 0.48 | -0.55 |
| w/o MS2:PI 40:6; [M+Na]+ | -0.76 | -0.09 | -0.26 | -0.48 | -0.20 | -0.17 | -0.46 | -0.88 |
| w/o MS2:PS 33:0; PS 6:0-27:0; [M+H]+ | 0.13 | -0.08 | 1.07 | 0.84 | 0.69 | 0.33 | 1.02 | 0.81 |
| w/o MS2:PS 38:9; [M+Na]+ | 0.17 | 0.01 | 0.49 | 0.61 | 0.63 | 0.13 | 0.31 | 0.67 |
| w/o MS2:PS 42:5; [M+Na]+ | 0.21 | 0.14 | 0.79 | 0.92 | 0.30 | 0.53 | 0.66 | 0.82 |

**Table S2**. Log2 fold changes for lipids (negative mode) from isolated cecal bacteria with POPs exposure for 4 h relative to vehicle.

| **Lipids** | **TCDF**  **0.6 µM** | **TCDF**  **6 µM** | **TCDD**  **0.06 µM** | **TCDF**  **0.6 µM** | **PCB 126**  **0.6 µM** | **PCB 126**  **6 µM** | **PCB 153**  **0.6 µM** | **PCB 153**  **6 µM** |
| --- | --- | --- | --- | --- | --- | --- | --- | --- |
| FA 17:0; [M-H]- | 0.15 | 0.13 | 0.26 | 0.18 | 0.22 | 0.36 | 0.39 | 0.28 |
| FA 18:1; [M-H]- | 0.27 | 0.18 | 0.50 | 0.44 | 0.31 | 0.55 | 0.53 | 0.60 |
| FA 20:2; [M-H]- | 0.38 | 0.09 | 0.77 | 0.49 | 0.73 | 0.53 | 0.39 | 0.52 |
| FA 20:3; [M-H]- | 0.12 | 0.15 | 0.46 | 0.20 | 0.49 | 0.59 | 0.49 | 0.68 |
| FA 22:1 | 0.31 | 0.11 | 0.39 | 0.36 | 0.24 | 0.38 | 0.34 | 0.32 |
| FA 22:5; [M-H]- | 0.08 | 0.16 | 0.69 | 0.36 | 0.51 | 0.27 | 0.24 | 0.41 |
| FA 22:6; [M-H]- | -0.31 | -0.89 | -0.76 | -0.68 | -0.46 | 0.02 | -0.71 | -0.33 |
| w/o MS2:FA 16:1; [M-H]- | 0.34 | 0.18 | 0.45 | 0.45 | 0.46 | 0.84 | 0.44 | 0.68 |
| w/o MS2:FA 20:0; [M-H]- | 0.28 | 0.19 | 0.47 | 0.33 | 0.22 | 0.33 | 0.42 | 0.39 |
| w/o MS2:FA 20:1; [M-H]- | 0.30 | 0.15 | 0.35 | 0.25 | 0.14 | 0.36 | 0.36 | 0.36 |
| w/o MS2:FA 20:5; [M-H]- | -0.29 | -0.95 | -0.74 | -0.63 | -0.34 | 0.20 | -0.55 | -0.24 |
| w/o MS2:FA 22:0; [M-H]- | 0.37 | 0.51 | 0.84 | 0.64 | 0.53 | 0.70 | 0.72 | 0.82 |
| w/o MS2:FA 22:4 | 0.36 | 0.14 | 0.69 | 0.42 | 0.61 | 0.47 | 0.30 | 0.33 |
| w/o MS2:FA 24:0; [M-H]- | 0.23 | 0.45 | 0.87 | 0.55 | 0.44 | 0.64 | 0.57 | 0.84 |
| w/o MS2:FA 24:1 | 0.21 | 0.20 | 0.40 | 0.49 | 0.22 | 0.31 | 0.32 | 0.45 |
| w/o MS2:FA 24:2; [M-H]- | 0.13 | 0.09 | 0.16 | 0.23 | 0.05 | 0.22 | 0.21 | 0.11 |
| w/o MS2:FA 24:6; [M-H]- | 0.07 | -0.23 | 0.02 | 0.11 | 0.04 | 0.29 | 0.08 | 0.27 |
| w/o MS2:FA 30:1; [M-H]- | 0.27 | 0.82 | 1.27 | 0.76 | 0.83 | 0.98 | 0.96 | 1.20 |
| w/o MS2:FA 32:1; [M-H]- | 0.22 | 0.68 | 0.99 | 0.70 | 0.48 | 0.89 | 0.46 | 1.00 |
| w/o MS2:FA 34:1; [M-H]- | -0.04 | 0.71 | 0.91 | 0.45 | 0.63 | 0.86 | 0.60 | 0.74 |
| FA 18:2; [M-H]- | -0.10 | -1.31 | -0.96 | -0.85 | -0.45 | 0.78 | -0.66 | -0.07 |
| FA 20:4; [M-H]- | -0.07 | -0.56 | -0.36 | -0.33 | -0.13 | 0.28 | -0.26 | 0.06 |
| w/o MS2:FA 18:3; [M-H]- | -0.23 | -0.80 | -0.49 | -0.42 | -0.34 | 0.50 | -0.41 | -0.11 |
| FAHFA 24:0; FAHFA 2:0/22:0; [M-H]- | 0.29 | 0.12 | 0.28 | 0.36 | 0.21 | 0.33 | 0.26 | 0.28 |
| FAHFA 26:0; FAHFA 2:0/24:0; [M-H]- | 0.06 | 0.06 | 0.25 | 0.39 | 0.05 | 0.25 | 0.25 | 0.27 |
| w/o MS2:FAHFA 18:0; FAHFA 15:0/3:0; [M-H]- | 0.30 | 0.16 | 0.38 | 0.44 | 0.35 | 0.35 | 0.30 | 0.19 |
| w/o MS2:FAHFA 18:0; FAHFA 3:0/15:0; [M-H]- | 0.06 | -0.07 | 0.27 | 0.32 | 0.11 | 0.36 | 0.41 | 0.52 |
| w/o MS2:FAHFA 2:0/22:3 | -0.41 | -0.96 | -0.41 | -0.40 | -0.34 | 0.60 | -0.28 | -0.52 |
| w/o MS2:FAHFA 21:3; FAHFA 5:0/16:3; [M-H]- | 0.28 | 0.20 | 0.66 | 0.47 | 0.60 | 0.63 | 0.69 | 0.63 |
| w/o MS2:FAHFA 22:0; FAHFA 19:0/3:0; [M-H]- | 0.28 | 0.08 | 0.24 | 0.23 | 0.06 | 0.19 | 0.20 | 0.19 |
| w/o MS2:FAHFA 22:5; FAHFA 20:5/2:0; [M-H]- | 0.27 | 0.11 | 0.61 | 0.59 | 0.20 | 0.54 | 0.58 | 0.48 |
| w/o MS2:FAHFA 22:6/7:0 | -0.18 | -0.34 | -0.14 | -0.01 | -0.43 | 0.05 | 0.01 | 0.01 |
| w/o MS2:FAHFA 23:3; FAHFA 7:0/16:3; [M-H]- | 0.24 | 0.24 | 0.69 | 0.35 | 0.58 | 0.55 | 0.61 | 0.54 |
| w/o MS2:FAHFA 25:0; FAHFA 22:0/3:0; [M-H]- | 0.16 | 0.00 | 0.34 | 0.43 | 0.09 | 0.33 | 0.45 | 0.34 |
| w/o MS2:FAHFA 26:1; FAHFA 22:1/4:0; [M-H]- | 0.18 | 0.06 | 0.22 | 0.23 | 0.01 | 0.21 | 0.12 | 0.04 |
| w/o MS2:FAHFA 27:0; FAHFA 24:0/3:0; [M-H]- | 0.15 | 0.37 | 0.38 | 0.61 | 0.16 | 0.26 | 0.38 | 0.37 |
| w/o MS2:FAHFA 27:1; FAHFA 24:1/3:0; [M-H]- | 0.09 | 0.07 | 0.29 | 0.39 | -0.02 | 0.34 | 0.56 | 0.28 |
| w/o MS2:FAHFA 27:3; FAHFA 22:3/5:0; [M-H]- | 0.41 | 0.18 | 0.65 | 0.48 | 0.53 | 0.49 | 0.65 | 0.50 |
| w/o MS2:FAHFA 28:0; FAHFA 25:0/3:0; [M-H]- | 0.15 | 0.60 | 0.03 | 0.68 | 0.20 | 0.25 | 0.77 | 0.45 |
| w/o MS2:FAHFA 28:1; FAHFA 24:1/4:0; [M-H]- | -0.12 | 0.38 | 0.12 | 0.29 | 0.03 | 0.22 | 0.71 | 0.12 |
| w/o MS2:FAHFA 28:3; FAHFA 16:3/12:0; [M-H]- | 0.12 | -0.03 | 0.22 | 0.26 | 0.10 | 0.17 | 0.23 | 0.10 |
| w/o MS2:FAHFA 28:4; FAHFA 24:4/4:0; [M-H]- | 0.16 | -0.05 | 0.15 | 0.34 | 0.11 | 0.15 | 0.41 | 0.17 |
| w/o MS2:FAHFA 30:0; FAHFA 27:0/3:0; [M-H]- | 0.18 | 0.74 | 0.44 | 0.60 | 0.18 | 0.28 | 0.84 | 0.50 |
| w/o MS2:FAHFA 30:6; FAHFA 22:6/8:0; [M-H]- | -0.21 | -0.01 | 0.04 | -0.06 | -0.35 | -0.13 | -0.06 | -0.24 |
| w/o MS2:FAHFA 34:0; FAHFA 25:0/9:0; [M-H]- | -0.49 | -1.34 | -0.69 | -0.61 | -0.69 | 0.23 | -0.80 | -0.51 |
| w/o MS2:FAHFA 36:0; FAHFA 27:0/9:0; [M-H]- | -0.74 | -1.47 | -0.75 | -0.86 | -0.72 | 0.03 | -1.04 | -0.73 |
| w/o MS2:FAHFA 36:1; FAHFA 15:0/21:1; [M-H]- | -0.82 | -1.43 | -0.54 | -0.65 | -0.52 | 0.38 | -0.84 | -0.84 |
| w/o MS2:FAHFA 36:2; FAHFA 15:0/21:2; [M-H]- | -0.41 | -1.49 | -0.83 | -0.66 | -0.68 | 0.54 | -0.88 | -0.55 |
| LPE 15:0; [M-H]- | -0.37 | -1.01 | -0.80 | -0.23 | -0.66 | -0.11 | -0.88 | -0.17 |
| LPE 18:0 | -0.80 | -1.81 | -1.76 | -1.06 | -1.15 | -0.48 | -1.71 | -0.83 |
| LPE 18:1e; [M-H]- | 0.15 | -0.73 | -0.38 | -0.13 | -0.29 | 0.45 | -0.40 | 0.16 |
| LPE 18:2e; [M-H]- | -0.64 | -1.58 | -1.51 | -0.67 | -1.05 | -0.06 | -1.22 | -0.51 |
| w/o MS2:LPE 18:0e; [M-H]- | -0.94 | -1.16 | -1.14 | -1.09 | -0.80 | -0.35 | -1.36 | -0.81 |
| w/o MS2:LPE 18:1; [M-H]- | -0.26 | -0.94 | -0.56 | 0.00 | -0.54 | -0.02 | -0.42 | 0.01 |
| w/o MS2:LPE 19:1e; [M-H]- | 0.04 | -0.77 | -0.40 | -0.16 | -0.32 | 0.30 | -0.33 | -0.02 |
| w/o MS2:LPE 20:0e; [M-H]- | -0.58 | -1.23 | -0.99 | -1.21 | -0.71 | -0.35 | -0.98 | -1.01 |
| w/o MS2:LPE 22:1e; [M-H]- | 0.08 | -0.46 | -0.11 | 0.08 | 0.17 | 0.45 | -0.12 | 0.12 |
| w/o MS2:LPE 22:2e; [M-H]- | 0.08 | -0.46 | -0.19 | 0.03 | -0.02 | 0.41 | -0.14 | 0.15 |
| w/o MS2:LPE 36:1; [M-H]- | -0.25 | -0.55 | -0.84 | -0.56 | 0.00 | -0.36 | -0.67 | -0.18 |
| w/o MS2:LPE 36:2; [M-H]- | 0.33 | -0.16 | 0.15 | 0.43 | 0.08 | 0.26 | 0.34 | 0.52 |
| w/o MS2:LPE 36:5; [M-H]- | 0.35 | -0.23 | 0.14 | 0.85 | 0.31 | 0.62 | 0.34 | 0.59 |
| w/o MS2:LPE 38:1; [M-H]- | -0.19 | -0.54 | -0.76 | -0.63 | 0.08 | -0.31 | -0.74 | -0.27 |
| w/o MS2:LPE 40:7; [M-H]- | 0.33 | 0.02 | 0.03 | 0.39 | 0.20 | 0.30 | 0.31 | 0.46 |
| w/o MS2:LPE 44:7; [M-H]- | 0.43 | 0.34 | 0.44 | 0.38 | 0.26 | 0.19 | 0.63 | 0.38 |
| w/o MS2:LPE O-22:1 | -0.33 | -0.73 | -0.65 | -0.57 | -0.25 | -0.05 | -0.63 | -0.18 |
| PE 32:1; PE 16:0-16:1; [M-H]- | 0.06 | -0.13 | -0.15 | -0.02 | -0.31 | -0.31 | 0.02 | -0.17 |
| PE 33:2; PE 15:0-18:2; [M-H]- | -0.27 | -0.26 | -0.62 | -0.62 | -0.56 | -0.39 | -0.40 | -0.49 |
| PE 34:1; PE 16:0-18:1; [M-H]- | -0.01 | -0.28 | 0.04 | 0.00 | -0.26 | -0.28 | 0.00 | -0.21 |
| PE 34:2; PE 16:0-18:2; [M-H]- | -0.19 | -0.23 | -0.43 | -0.35 | -0.54 | -0.16 | -0.07 | -0.50 |
| PE 36:1\|PE 18:0_18:1 | 0.28 | -0.36 | 0.25 | 0.65 | 0.26 | 0.49 | 0.35 | 0.64 |
| w/o MS2:PE 27:0; PE 8:0-19:0; [M-H]- | 0.15 | 0.21 | 0.24 | 0.10 | -0.13 | -0.12 | 0.19 | -0.04 |
| w/o MS2:PE 31:1e; PE 9:0e/22:1; [M-H]- | 0.18 | 0.18 | -0.12 | -0.10 | -0.22 | 0.08 | -0.24 | -0.32 |
| w/o MS2:PE 32:2; PE 8:0-24:2; [M-H]- | -0.21 | -0.20 | -0.45 | -0.58 | -0.61 | -0.27 | -0.39 | -0.46 |
| w/o MS2:PE 35:4; PE 9:0-26:4; [M-H]- | -0.33 | -0.47 | -0.46 | -0.59 | -0.46 | -0.46 | -0.55 | -0.70 |
| w/o MS2:PE 36:3; PE 8:0-28:3; [M-H]- | -0.16 | -0.05 | -0.10 | -0.12 | -0.31 | -0.37 | -0.01 | -0.26 |
| w/o MS2:PE 36:4; PE 8:0-28:4; [M-H]- | -0.12 | -0.37 | -0.19 | -0.06 | -0.24 | -0.17 | -0.38 | -0.19 |
| w/o MS2:PE O-10:0_28:5 | 0.44 | -0.22 | 0.23 | 0.91 | 0.44 | 0.66 | 0.50 | 0.76 |
| w/o MS2:PE O-12:0_28:4 | -0.26 | -0.77 | -0.68 | -0.39 | -0.10 | -0.25 | -0.43 | -0.09 |
| w/o MS2:PE O-16:0_20:5;O | -0.34 | -0.31 | -0.30 | -0.39 | -0.37 | -0.44 | -0.40 | -0.67 |
| w/o MS2:PE O-18:0_20:3;2O | 0.08 | -0.13 | -0.25 | -0.28 | -0.27 | -0.11 | -0.05 | -0.18 |
| w/o MS2:PE O-8:0_28:2 | 0.61 | 0.33 | 0.55 | 0.86 | 0.67 | 0.44 | 0.72 | 0.89 |
| w/o MS2:PE O-8:0_28:3 | 0.43 | 0.21 | 0.43 | 0.63 | 0.48 | 0.49 | 0.58 | 0.66 |
| w/o MS2:PE-Cer d36:1; PE-Cer d12:1/24:0; [M-H]- | -0.25 | -0.86 | -0.59 | -0.47 | -0.18 | -0.04 | -0.47 | -0.11 |
| PG 31:0\|PG 15:0_16:0 | 0.60 | 0.80 | 0.84 | 0.72 | 0.53 | 0.18 | 0.67 | 0.48 |
| PG 32:0; PG 16:0-16:0; [M-H]- | 0.69 | 0.68 | 0.60 | 0.80 | 0.57 | 0.37 | 0.94 | 0.68 |
| PG 32:1e; PG 16:1e/16:0; [M-H]- | 1.07 | 1.10 | 0.98 | 1.15 | 0.96 | -0.37 | 1.09 | 0.88 |
| PG 36:1\|PG 18:0_18:1 | 0.74 | 1.07 | 1.21 | 0.93 | 0.74 | 0.26 | 1.06 | 0.69 |
| PG O-30:1\|PG O-14:1_16:0 | 0.55 | 0.70 | 0.82 | 0.54 | 0.40 | 0.34 | 0.59 | 0.44 |
| w/o MS2:PG 30:0; PG 3:0-27:0; [M-H]- | 0.58 | 0.78 | 0.97 | 0.82 | 0.67 | 0.33 | 0.90 | 0.62 |
| w/o MS2:PG 31:1e; PG 10:0e/21:1; [M-H]- | 0.56 | 1.14 | 1.10 | 0.88 | 0.83 | 0.32 | 0.73 | 0.71 |
| w/o MS2:PG 32:0; PG 5:0-27:0; [M-H]- | 0.22 | -0.10 | 0.03 | 0.01 | 0.04 | -0.04 | 0.26 | 0.18 |
| w/o MS2:PG 32:2e; PG 18:2e/14:0; [M-H]- | -0.17 | -0.36 | -0.38 | -0.65 | -0.41 | -0.36 | -0.29 | -0.55 |
| w/o MS2:PG 32:3e; PG 10:0e/22:3; [M-H]- | -0.12 | -0.36 | -0.57 | -0.73 | -0.42 | -0.07 | -0.45 | -0.55 |
| w/o MS2:PG 34:0; PG 7:0-27:0; [M-H]- | 0.72 | 0.72 | 0.93 | 0.71 | 0.70 | 0.35 | 0.66 | 0.72 |
| w/o MS2:PG 34:2e; PG 10:0e/24:2; [M-H]- | 0.66 | 0.99 | 0.42 | 0.70 | 0.68 | 0.19 | 0.88 | 0.50 |
| w/o MS2:PG 40:8; PG 22:3-18:5; [M-H]- | -0.65 | -1.04 | -0.52 | -0.51 | -0.56 | -0.37 | -0.80 | -0.51 |
| w/o MS2:PG 45:1; PG 27:0-18:1; [M-H]- | 0.47 | 0.57 | 0.35 | 0.50 | 0.05 | -0.11 | 0.08 | -0.39 |
| w/o MS2:PG 47:1; PG 27:0-20:1; [M-H]- | 0.32 | 0.44 | 0.05 | 0.21 | -0.18 | -0.10 | 0.00 | -0.44 |
| w/o MS2:PG 48:2; PG 27:0-21:2; [M-H]- | 0.40 | 0.69 | 0.04 | 0.36 | 0.10 | -0.23 | 0.40 | -0.05 |
| w/o MS2:PG 49:2; PG 27:0-22:2; [M-H]- | 0.52 | 0.72 | 0.61 | 0.20 | 0.26 | 0.13 | 0.52 | -0.13 |
| w/o MS2:PG 49:3; PG 27:0-22:3; [M-H]- | 0.28 | 0.32 | 0.34 | 0.16 | 0.05 | -0.08 | 0.23 | 0.11 |
| w/o MS2:PG 6:0_27:0 | 0.85 | 1.21 | 1.37 | 1.12 | 1.04 | 0.45 | 1.29 | 0.94 |
| w/o MS2:PG 9:0_27:0 | 0.41 | 0.29 | 0.59 | 0.65 | 0.46 | 0.37 | 0.54 | 0.47 |
| w/o MS2:PA 33:0; PA 6:0-27:0; [M-H]- | 0.03 | -0.20 | -0.39 | -0.24 | -0.28 | 0.02 | 0.03 | -0.35 |
| w/o MS2:PA 34:0; PA 7:0-27:0; [M-H]- | -0.11 | -0.28 | -0.36 | -0.33 | -0.37 | -0.05 | -0.16 | -0.37 |
| w/o MS2:PA 37:1; PA 11:0-26:1; [M-H]- | -0.27 | -0.40 | -0.43 | -0.43 | -0.52 | -0.02 | -0.33 | -0.59 |
| w/o MS2:PC 31:2e; PC 9:0e/22:2; [M+HCOO]- | -0.06 | -0.20 | -0.84 | -0.53 | -0.52 | -0.52 | -0.45 | -0.29 |
| w/o MS2:PC 8:0_25:0 | 0.22 | -0.02 | 0.14 | 0.02 | 0.02 | 0.23 | 0.22 | 0.08 |
| w/o MS2:PC O-16:0_20:3;O | -0.04 | -0.17 | -0.21 | -0.28 | -0.69 | -0.13 | -0.14 | -0.21 |
| w/o MS2:PC O-18:0_20:3;3O | 0.04 | 0.08 | -0.45 | -0.31 | -0.26 | -0.19 | -0.17 | -0.33 |
| w/o MS2:PI O-10:0_24:0 | 0.59 | 0.15 | 0.38 | 0.10 | 0.25 | 0.03 | 0.29 | 0.44 |
| w/o MS2:PI O-10:0_24:1 | 0.40 | 0.11 | 0.13 | 0.14 | 0.02 | 0.14 | 0.20 | 0.36 |

**Table S3**. Metatranscriptomics analysis of five representative genera of gut microbiota.

| Bacteria | Total number of genes identified | Low abundance filter |
| --- | --- | --- |
| *Bacteroides* | 4434 | 705 |
| *Clostridium* | 2755 | 96 |
| *Lactobacillus* | 904 | 41 |
| *Bifidobacterium* | 785 | 15 |
| *Fusobacterium* | 188 | 2 |

**Table S4**. Metatranscriptomics analysis of *Bacteroides* with POPs exposure. The differentially expressed gene orthologs for each POP was calculated using student t test. Multiple tests correction was performed by calculating False Discovery Rate-adjusted (FDR) p-value using Benjamini-Hochberg method with a threshold of p.value < 0.05.

| **Vehicle vs TCDF (6 µM)** | | |
| --- | --- | --- |
| **Gene Name** | **p** | **FDR** |
| alpha-1,2-mannosidase | 7.32E-06 | 0.0039739 |
| alpha-mannosidase | 2.40E-05 | 0.0054405 |
| META domain-containing protein | 3.35E-05 | 0.0054405 |
| MULTISPECIES: malate dehydrogenase | 4.92E-05 | 0.0054405 |
| malate dehydrogenase | 5.84E-05 | 0.0054405 |
| MULTISPECIES: glutamate decarboxylase | 7.08E-05 | 0.0054405 |
| MULTISPECIES: 2,3-bisphosphoglycerate-independent phosphoglycerate mutase | 8.12E-05 | 0.0054405 |
| MULTISPECIES: elongation factor Tu | 8.68E-05 | 0.0054405 |
| glycerol-3-phosphate transporter | 9.02E-05 | 0.0054405 |
| elongation factor G | 0.00010783 | 0.0058554 |
| urocanate hydratase | 0.00012825 | 0.0063307 |
| glutamate decarboxylase | 0.00014353 | 0.0064945 |
| F0F1 ATP synthase subunit beta | 0.0004103 | 0.016423 |
| cytidine deaminase | 0.00042343 | 0.016423 |
| DNA-directed RNA polymerase subunit beta | 0.00046026 | 0.016662 |
| phosphogluconate dehydrogenase (NADP(+)-dependent, decarboxylating) | 0.00053608 | 0.018193 |
| MULTISPECIES: OmpA family protein | 0.00065378 | 0.020882 |
| alpha-2-macroglobulin | 0.00072769 | 0.021952 |
| conserved domain protein | 0.0010647 | 0.02931 |
| aspartate ammonia-lyase | 0.0010796 | 0.02931 |
| 50S ribosomal protein L10 | 0.0014182 | 0.035048 |
| acetyl-CoA carboxylase biotin carboxylase subunit | 0.0014462 | 0.035048 |
| thiamine-phosphate kinase | 0.0014845 | 0.035048 |
| ABC transporter permease | 0.001615 | 0.036539 |
| MULTISPECIES: fructose-1,6-bisphosphate aldolase, class II | 0.0017618 | 0.038267 |
| alpha-L-arabinofuranosidase | 0.0020153 | 0.042088 |
| NodT family efflux transporter outer membrane factor (OMF) lipoprotein | 0.0021611 | 0.043462 |
| aldo/keto reductase | 0.0023818 | 0.046189 |
| F0F1 ATP synthase subunit alpha | 0.0026987 | 0.050531 |
| glutamate decarboxylase, partial | 0.0032137 | 0.054871 |
| MULTISPECIES: fructose-bisphosphate aldolase | 0.0033254 | 0.054871 |
| DJ-1 family protein | 0.0033707 | 0.054871 |
| glutaminase | 0.0034082 | 0.054871 |
| MULTISPECIES: peroxiredoxin | 0.0034872 | 0.054871 |
| lipopolysaccharide biosynthesis protein | 0.0035368 | 0.054871 |
| cell division protein FtsH | 0.0036696 | 0.05535 |
| transporter | 0.0040738 | 0.059785 |
| peptidase S8 | 0.0044105 | 0.063024 |
| amino acid transporter | 0.0047322 | 0.065886 |
| protein translocase subunit SecDF | 0.0052984 | 0.070868 |
| ribosome biogenesis protein BMS1 | 0.005351 | 0.070868 |
| MULTISPECIES: glucose-6-phosphate dehydrogenase | 0.0059313 | 0.076684 |
| MULTISPECIES: hypothetical protein | 0.0069383 | 0.087616 |
| MULTISPECIES: xylose isomerase | 0.0071781 | 0.088584 |
| MULTISPECIES: elongation factor G | 0.0085139 | 0.10031 |
| phosphate porin | 0.0086103 | 0.10031 |
| DUF4302 domain-containing protein | 0.0086853 | 0.10031 |
| OmpA family protein | 0.0091318 | 0.10031 |
| polyribonucleotide nucleotidyltransferase | 0.009297 | 0.10031 |
| Fic family protein | 0.0094155 | 0.10031 |
| multidrug efflux RND transporter permease subunit | 0.0094218 | 0.10031 |
| MULTISPECIES: glutamate:gamma-aminobutyrate antiporter | 0.010065 | 0.1051 |
| pyruvate:ferredoxin (flavodoxin) oxidoreductase, partial | 0.010648 | 0.10909 |
| MULTISPECIES: porin family protein | 0.011277 | 0.1134 |
| pyruvate, phosphate dikinase | 0.011739 | 0.11582 |
| amino acid permease | 0.011944 | 0.11582 |
| beta-glucosidase | 0.01246 | 0.1187 |
| glycosyl hydrolase family 43 | 0.012991 | 0.12162 |
| fumarate hydratase | 0.013236 | 0.12182 |
| pirin family protein | 0.01445 | 0.13078 |
| insulinase family protein | 0.014813 | 0.13186 |
| aminoacyl-histidine dipeptidase | 0.015693 | 0.136 |
| AAA family ATPase | 0.016292 | 0.136 |
| DNA polymerase III subunit alpha | 0.016495 | 0.136 |
| elongation factor G, partial | 0.016967 | 0.136 |
| superoxide dismutase | 0.017144 | 0.136 |
| ATP synthase subunit A | 0.017325 | 0.136 |
| MULTISPECIES: TonB-dependent receptor | 0.01738 | 0.136 |
| AcrB/AcrD/AcrF family protein | 0.017699 | 0.136 |
| DUF4458 domain-containing protein | 0.01778 | 0.136 |
| MULTISPECIES: ROK family protein | 0.018028 | 0.136 |
| GlsB/YeaQ/YmgE family stress response membrane protein | 0.018135 | 0.136 |
| rubrerythrin family protein | 0.018283 | 0.136 |
| XRE family transcriptional regulator | 0.018713 | 0.13731 |
| DUF3078 domain-containing protein | 0.019262 | 0.13946 |
| Na+/glucose cotransporter | 0.020224 | 0.14286 |
| bifunctional metallophosphatase/5'-nucleotidase | 0.020259 | 0.14286 |
| metal-independent alpha-mannosidase | 0.020713 | 0.14419 |
| MULTISPECIES: tyrosine recombinase XerC | 0.021356 | 0.14494 |
| tellurium resistance protein TerD | 0.021371 | 0.14494 |
| transposase | 0.02162 | 0.14494 |
| MULTISPECIES: DNA gyrase subunit A | 0.022792 | 0.14992 |
| glycerophosphodiester phosphodiesterase | 0.023169 | 0.14992 |
| TIGR01212 family radical SAM protein | 0.023192 | 0.14992 |
| MULTISPECIES: DUF5006 domain-containing protein | 0.02358 | 0.15064 |
| phosphoribosylformylglycinamidine synthase | 0.02456 | 0.15396 |
| malate dehydrogenase, partial | 0.025027 | 0.15396 |
| capsule biosynthesis protein | 0.025129 | 0.15396 |
| glycerophosphoryl diester phosphodiesterase | 0.025295 | 0.15396 |
| asparagine--tRNA ligase | 0.025849 | 0.15396 |
| cation transporter | 0.025955 | 0.15396 |
| phospho-sugar mutase | 0.026086 | 0.15396 |
| glutamate:gamma-aminobutyrate antiporter | 0.027251 | 0.15911 |
| MULTISPECIES: asparagine--tRNA ligase | 0.02799 | 0.16169 |
| translation elongation factor Tu | 0.029191 | 0.16685 |
| 2',3'-cyclic-nucleotide 2'-phosphodiesterase | 0.031157 | 0.17612 |
| peptidase M13 | 0.031715 | 0.17612 |
| helicase | 0.031786 | 0.17612 |
| MULTISPECIES: nucleotidyltransferase | 0.036576 | 0.19799 |
| glucosamine-6-phosphate deaminase | 0.03666 | 0.19799 |
| alpha-mannosidase, partial | 0.037039 | 0.19799 |
| DUF4136 domain-containing protein | 0.037191 | 0.19799 |
| peptidase C10 family protein | 0.038489 | 0.20023 |
| aspartate--tRNA ligase | 0.038844 | 0.20023 |
| 2,3-bisphosphoglycerate-independent phosphoglycerate mutase | 0.039318 | 0.20023 |
| arginine--tRNA ligase | 0.039578 | 0.20023 |
| cysteine synthase A | 0.03969 | 0.20023 |
| phosphoglycerate kinase | 0.040513 | 0.20023 |
| MULTISPECIES: glycosyltransferase family 1 protein | 0.040536 | 0.20023 |
| phosphopyruvate hydratase | 0.040563 | 0.20023 |
| beta-ketoacyl | 0.041143 | 0.20127 |
| N-acetyltransferase | 0.042401 | 0.20557 |
| MBL fold metallo-hydrolase | 0.043496 | 0.20901 |
| MULTISPECIES: DUF4302 domain-containing protein | 0.044323 | 0.21032 |
| MULTISPECIES: pyridoxal phosphate-dependent aminotransferase | 0.044693 | 0.21032 |
| DUF4332 domain-containing protein | 0.045181 | 0.21032 |
| carbamoyl-phosphate synthase (glutamine-hydrolyzing) large subunit | 0.045318 | 0.21032 |
| RNA-binding protein, partial | 0.046305 | 0.21304 |
| MULTISPECIES: glutaminase | 0.046885 | 0.21304 |
| succinate dehydrogenase flavoprotein subunit | 0.047082 | 0.21304 |
| glycosyl hydrolase family 10 | 0.048429 | 0.21701 |
| glutamine synthetase | 0.049142 | 0.21701 |
| integration host factor subunit beta | 0.049369 | 0.21701 |
| preprotein translocase subunit SecY | 0.049855 | 0.21701 |
| alpha-xylosidase | 0.049956 | 0.21701 |
| **Vehicle vs TCDD (0.6 µM)** | | |
| **Gene Name** | **p** | **FDR** |
| MULTISPECIES: glutamate decarboxylase | 1.16E-05 | 0.0035036 |
| glycerol-3-phosphate transporter | 1.84E-05 | 0.0035036 |
| glutamate decarboxylase | 2.55E-05 | 0.0035036 |
| MULTISPECIES: malate dehydrogenase | 3.07E-05 | 0.0035036 |
| malate dehydrogenase | 3.36E-05 | 0.0035036 |
| META domain-containing protein | 0.00045188 | 0.039238 |
| peroxiredoxin | 0.001066 | 0.065902 |
| alpha-mannosidase | 0.0010927 | 0.065902 |
| aspartate ammonia-lyase | 0.0011568 | 0.065902 |
| pyruvate:ferredoxin (flavodoxin) oxidoreductase, partial | 0.0012649 | 0.065902 |
| metal-independent alpha-mannosidase | 0.0015421 | 0.066711 |
| glutamate decarboxylase, partial | 0.0017135 | 0.066711 |
| phosphogluconate dehydrogenase (NADP(+)-dependent, decarboxylating) | 0.001759 | 0.066711 |
| MULTISPECIES: 2,3-bisphosphoglycerate-independent phosphoglycerate mutase | 0.0018686 | 0.066711 |
| thiamine-phosphate kinase | 0.0019709 | 0.066711 |
| GMP synthase (glutamine-hydrolyzing) | 0.0021585 | 0.066711 |
| MULTISPECIES: protease | 0.0021767 | 0.066711 |
| NodT family efflux transporter outer membrane factor (OMF) lipoprotein | 0.0024391 | 0.070599 |
| MULTISPECIES: peroxiredoxin | 0.0039203 | 0.096811 |
| DJ-1 family protein | 0.0040134 | 0.096811 |
| phosphoglycerate kinase | 0.0041957 | 0.096811 |
| MULTISPECIES: fructose-1,6-bisphosphate aldolase, class II | 0.0045809 | 0.096811 |
| urocanate hydratase | 0.004699 | 0.096811 |
| MULTISPECIES: tyrosine recombinase XerC | 0.0047248 | 0.096811 |
| glycoside hydrolase family 2 | 0.0048256 | 0.096811 |
| DUF3078 domain-containing protein | 0.0048313 | 0.096811 |
| MULTISPECIES: transposase | 0.0054647 | 0.10545 |
| ribonuclease R | 0.0067508 | 0.12561 |
| peptidase S8 | 0.0073477 | 0.13201 |
| amino acid transporter | 0.0087633 | 0.15219 |
| MULTISPECIES: xylose isomerase | 0.0095871 | 0.16112 |
| bifunctional (p)ppGpp synthetase/guanosine-3',5'-bis(diphosphate) 3'-pyrophosphohydrolase | 0.010326 | 0.16813 |
| MULTISPECIES: glucose-6-phosphate dehydrogenase | 0.010906 | 0.17218 |
| alpha-1,2-mannosidase | 0.011806 | 0.18091 |
| conserved domain protein | 0.012309 | 0.18323 |
| DUF2851 domain-containing protein | 0.013381 | 0.19156 |
| glycosyl hydrolase family 10 | 0.013604 | 0.19156 |
| ABC transporter permease | 0.015577 | 0.21357 |
| aminopeptidase P family protein | 0.016208 | 0.21653 |
| peptidase M13 | 0.016919 | 0.22037 |
| pyruvate, phosphate dikinase | 0.017836 | 0.22664 |
| glutaminase | 0.019242 | 0.23869 |
| carbohydrate kinase | 0.021003 | 0.24238 |
| glycoside hydrolase | 0.021655 | 0.24238 |
| polyprenol monophosphomannose synthase | 0.021726 | 0.24238 |
| chloride channel protein | 0.022534 | 0.24238 |
| 50S ribosomal protein L10 | 0.024052 | 0.24238 |
| DUF3943 domain-containing protein | 0.024544 | 0.24238 |
| alpha-N-arabinofuranosidase | 0.024569 | 0.24238 |
| MULTISPECIES: XRE family transcriptional regulator | 0.024837 | 0.24238 |
| amino acid permease | 0.024889 | 0.24238 |
| citrate/2-methylcitrate synthase | 0.025286 | 0.24238 |
| protein phosphatase 2C domain-containing protein | 0.02546 | 0.24238 |
| aspartate--tRNA ligase | 0.026038 | 0.24238 |
| arabinogalactan endo-1,4-beta-galactosidase | 0.026213 | 0.24238 |
| MULTISPECIES: 30S ribosomal protein S15 | 0.027059 | 0.24238 |
| acetyl-CoA carboxylase biotin carboxylase subunit | 0.027323 | 0.24238 |
| cation transporter | 0.027639 | 0.24238 |
| glutamate 5-kinase | 0.02818 | 0.24238 |
| ribosome biogenesis protein BMS1 | 0.028835 | 0.24238 |
| peptidase M16 | 0.029497 | 0.24238 |
| malate dehydrogenase, partial | 0.029862 | 0.24238 |
| ABC transporter ATP-binding protein | 0.030089 | 0.24238 |
| MULTISPECIES: Fe-S cluster assembly protein SufB | 0.032103 | 0.24238 |
| hypothetical protein | 0.032448 | 0.24238 |
| phosphate porin | 0.032937 | 0.24238 |
| MULTISPECIES: molecular chaperone HtpG | 0.032949 | 0.24238 |
| enolase | 0.033095 | 0.24238 |
| aldo/keto reductase | 0.033218 | 0.24238 |
| protease | 0.033332 | 0.24238 |
| glutamate dehydrogenase | 0.033411 | 0.24238 |
| OmpA family protein | 0.034221 | 0.24238 |
| glycerophosphoryl diester phosphodiesterase | 0.034485 | 0.24238 |
| type I glyceraldehyde-3-phosphate dehydrogenase | 0.035179 | 0.24238 |
| tyrosine recombinase XerC | 0.035762 | 0.24238 |
| phosphoribosylformylglycinamidine synthase | 0.037022 | 0.24238 |
| ATP-binding protein | 0.037142 | 0.24238 |
| MULTISPECIES: L-asparaginase 2 | 0.037405 | 0.24238 |
| glutamine synthetase | 0.037476 | 0.24238 |
| MULTISPECIES: cold-shock protein | 0.037852 | 0.24238 |
| phospho-sugar mutase | 0.038259 | 0.24238 |
| AcrB/AcrD/AcrF family protein | 0.038604 | 0.24238 |
| GlsB/YeaQ/YmgE family stress response membrane protein | 0.038613 | 0.24238 |
| glycerophosphodiester phosphodiesterase | 0.039212 | 0.24321 |
| RNA polymerase subunit sigma | 0.040314 | 0.2471 |
| 2',3'-cyclic-nucleotide 2'-phosphodiesterase | 0.04102 | 0.2485 |
| aspartate-alanine antiporter | 0.042576 | 0.25301 |
| NADH-dependent alcohol dehydrogenase | 0.042735 | 0.25301 |
| aminoacyl-histidine dipeptidase | 0.043694 | 0.25578 |
| insulinase family protein | 0.044237 | 0.25608 |
| fructose-bisphosphate aldolase | 0.046103 | 0.26134 |
| glutamate:gamma-aminobutyrate antiporter | 0.046148 | 0.26134 |
| DUF4136 domain-containing protein | 0.047112 | 0.26393 |
| TIGR01212 family radical SAM protein | 0.047936 | 0.26569 |
| phosphoserine phosphatase SerB | 0.048565 | 0.26634 |
| alanine dehydrogenase | 0.049767 | 0.27009 |
| **Vehicle vs PCB 126 (6 µM)** | | |
| **Gene Name** | **p** | **FDR** |
| malate dehydrogenase | 1.88E-05 | 0.0090544 |
| MULTISPECIES: 2,3-bisphosphoglycerate-independent phosphoglycerate mutase | 3.22E-05 | 0.0090544 |
| alpha-L-arabinofuranosidase | 0.00010178 | 0.019067 |
| glutamate decarboxylase | 0.0001559 | 0.019184 |
| MULTISPECIES: glutamate decarboxylase | 0.00017067 | 0.019184 |
| MULTISPECIES: malate dehydrogenase | 0.00048096 | 0.04505 |
| malate dehydrogenase | 1.88E-05 | 0.0090544 |
| MULTISPECIES: 2,3-bisphosphoglycerate-independent phosphoglycerate mutase | 3.22E-05 | 0.0090544 |
| alpha-L-arabinofuranosidase | 0.00010178 | 0.019067 |
| glutamate decarboxylase | 0.0001559 | 0.019184 |
| MULTISPECIES: glutamate decarboxylase | 0.00017067 | 0.019184 |
| MULTISPECIES: malate dehydrogenase | 0.00048096 | 0.04505 |
| **Vehicle vs PCB 153 (6 µM)** | | |
| **Gene Name** | **p** | **FDR** |
| MULTISPECIES: glutamate decarboxylase | 0.00017526 | 0.024638 |
| glycerol-3-phosphate transporter | 0.00019496 | 0.024638 |
| undecaprenyl/decaprenyl-phosphate alpha-N-acetylglucosaminyl 1-phosphate transferase | 0.00020269 | 0.024638 |
| META domain-containing protein | 0.00021987 | 0.024638 |
| alpha-mannosidase | 0.00024166 | 0.024638 |
| glutamate decarboxylase | 0.00030179 | 0.024638 |
| malate dehydrogenase | 0.00032327 | 0.024638 |
| peptidase S8 | 0.00033423 | 0.024638 |
| lipopolysaccharide biosynthesis protein | 0.00033616 | 0.024638 |
| MULTISPECIES: malate dehydrogenase | 0.00034898 | 0.024638 |
| glutamate decarboxylase, partial | 0.00052749 | 0.031034 |
| phosphoglycerate kinase | 0.00052749 | 0.031034 |
| alpha-L-arabinofuranosidase | 0.00062514 | 0.03395 |
| MULTISPECIES: 2,3-bisphosphoglycerate-independent phosphoglycerate mutase | 0.00076857 | 0.036941 |
| transcriptional repressor | 0.00082752 | 0.036941 |
| glutaminase | 0.00086368 | 0.036941 |
| elongation factor G | 0.00093711 | 0.036941 |
| alpha-2-macroglobulin | 0.0010226 | 0.036941 |
| amino acid transporter | 0.0010776 | 0.036941 |
| MULTISPECIES: xylose isomerase | 0.001103 | 0.036941 |
| DUF4458 domain-containing protein | 0.0011356 | 0.036941 |
| MULTISPECIES: 50S ribosomal protein L35 | 0.0011666 | 0.036941 |
| anticodon nuclease | 0.0012423 | 0.036941 |
| conserved domain protein | 0.0012558 | 0.036941 |
| urocanate hydratase | 0.0015235 | 0.042466 |
| thiamine-phosphate kinase | 0.0015804 | 0.042466 |
| DUF2971 domain-containing protein | 0.0016241 | 0.042466 |
| aminotransferase class I and II | 0.0018009 | 0.044275 |
| MULTISPECIES: porin family protein | 0.0020301 | 0.044275 |
| pyruvate:ferredoxin (flavodoxin) oxidoreductase, partial | 0.002034 | 0.044275 |
| site-specific DNA-methyltransferase | 0.0021204 | 0.044275 |
| malate dehydrogenase, partial | 0.0021714 | 0.044275 |
| DEAD/DEAH box helicase | 0.0022258 | 0.044275 |
| phospho-sugar mutase | 0.002309 | 0.044275 |
| glutamate dehydrogenase | 0.0023268 | 0.044275 |
| MFS transporter | 0.0023673 | 0.044275 |
| bifunctional metallophosphatase/5'-nucleotidase | 0.0023947 | 0.044275 |
| phosphoenolpyruvate carboxykinase (ATP) | 0.0024411 | 0.044275 |
| glycoside hydrolase family 2 | 0.0024458 | 0.044275 |
| pyruvate:ferredoxin (flavodoxin) oxidoreductase | 0.0027072 | 0.046427 |
| DNA-directed RNA polymerase subunit beta | 0.0028129 | 0.046427 |
| type I glyceraldehyde-3-phosphate dehydrogenase | 0.0030951 | 0.046427 |
| GTP-binding protein | 0.0031379 | 0.046427 |
| glycerophosphoryl diester phosphodiesterase | 0.0031488 | 0.046427 |
| glycosyltransferase family 2 protein | 0.0031669 | 0.046427 |
| TonB-dependent receptor | 0.003173 | 0.046427 |
| enoyl-ACP reductase | 0.0031834 | 0.046427 |
| DUF1573 domain-containing protein | 0.0032593 | 0.046427 |
| nucleoside-diphosphate-sugar epimerase | 0.0033516 | 0.046427 |
| DJ-1 family protein | 0.0034444 | 0.046427 |
| subtilase | 0.0034534 | 0.046427 |
| MULTISPECIES: TonB-dependent receptor | 0.0034708 | 0.046427 |
| inositol-3-phosphate synthase | 0.0035308 | 0.046427 |
| alpha-1,2-mannosidase | 0.0035511 | 0.046427 |
| Other | 0.0037102 | 0.046623 |
| 1,4-alpha-glucan-branching enzyme | 0.0037672 | 0.046623 |
| glutamine synthetase | 0.0038543 | 0.046623 |
| alpha-glucuronidase | 0.0038935 | 0.046623 |
| DUF4136 domain-containing protein | 0.0039584 | 0.046623 |
| DUF3078 domain-containing protein | 0.0039826 | 0.046623 |
| NodT family efflux transporter outer membrane factor (OMF) lipoprotein | 0.0040283 | 0.046623 |
| NADH peroxidase | 0.0041276 | 0.047001 |

**Table S5**. Metatranscriptomics analysis of *Clostridium* with POPs exposure. The differentially expressed gene orthologs for each POP was calculated using student t test. Multiple tests correction was performed by calculating False Discovery Rate-adjusted (FDR) p-value using Benjamini-Hochberg method with a threshold of p.value < 0.05.

| **Vehicle vs TCDF (6 µM)** | | |
| --- | --- | --- |
| **Gene Name** | **p** | **FDR** |
| pyruvate:ferredoxin (flavodoxin) oxidoreductase | 0.00010592 | 0.0082621 |
| phosphate acetyltransferase | 0.00056666 | 0.0221 |
| electron transfer flavoprotein subunit alpha/FixB family protein | 0.00088729 | 0.02307 |
| DNA-binding response regulator | 0.0013767 | 0.026845 |
| 2-hydroxyacyl-CoA dehydratase | 0.002086 | 0.032311 |
| glutamate dehydrogenase | 0.0024855 | 0.032311 |
| ABC transporter ATP-binding protein | 0.0055081 | 0.061376 |
| elongation factor Tu | 0.012836 | 0.12515 |
| peroxiredoxin | 0.019812 | 0.17171 |
| heat-inducible transcription repressor HrcA | 0.02426 | 0.18067 |
| rubrerythrin family protein | 0.028487 | 0.18067 |
| formate--tetrahydrofolate ligase | 0.033545 | 0.18067 |
| IS21 family transposase | 0.033771 | 0.18067 |
| efflux RND transporter periplasmic adaptor subunit | 0.034531 | 0.18067 |
| Other | 0.036414 | 0.18067 |
| PLP-dependent aminotransferase family protein | 0.037061 | 0.18067 |
| molecular chaperone DnaK | 0.042165 | 0.19347 |
| sugar ABC transporter ATP-binding protein | 0.045002 | 0.19501 |
| MBL fold metallo-hydrolase | 0.049476 | 0.20071 |
| **Vehicle vs TCDD (0.6 µM)** | | |
| **Gene Name** | **p** | **FDR** |
| pyruvate:ferredoxin (flavodoxin) oxidoreductase | 2.56E-05 | 0.001831 |
| histidinol dehydrogenase | 5.77E-05 | 0.001831 |
| PLP-dependent aminotransferase family protein | 6.10E-05 | 0.001831 |
| signal recognition particle protein | 0.00010941 | 0.0021222 |
| phosphate acetyltransferase | 0.00014424 | 0.0021222 |
| acetylglutamate kinase | 0.00016014 | 0.0021222 |
| electron transfer flavoprotein subunit alpha/FixB family protein | 0.00016506 | 0.0021222 |
| phosphoglycerate dehydrogenase | 0.00027259 | 0.0030666 |
| ATP-dependent chaperone ClpB | 0.00060778 | 0.0060723 |
| glutamate dehydrogenase | 0.0006747 | 0.0060723 |
| benzoyl-CoA reductase subunit D | 0.0017569 | 0.013624 |
| 2-hydroxyacyl-CoA dehydratase | 0.0020119 | 0.013624 |
| ABC transporter ATP-binding protein | 0.0021597 | 0.013624 |
| 3-methyl-2-oxobutanoate dehydrogenase subunit VorB | 0.0022566 | 0.013624 |
| IS21 family transposase | 0.0022706 | 0.013624 |
| AcrB/AcrD/AcrF family protein | 0.0030875 | 0.017367 |
| molecular chaperone HtpG | 0.0044029 | 0.023309 |
| efflux RND transporter periplasmic adaptor subunit | 0.0057455 | 0.028728 |
| **Vehicle vs PCB 126 (6 µM)** | | |
| **Gene Name** | **p** | **FDR** |
| PLP-dependent aminotransferase family protein | 0.004046 | 0.15883 |
| butyryl-CoA:acetate CoA-transferase | 0.004153 | 0.15883 |
| oxalyl-CoA decarboxylase | 0.0052952 | 0.15883 |
| formate--tetrahydrofolate ligase | 0.013675 | 0.15883 |
| benzoyl-CoA reductase subunit D | 0.014066 | 0.15883 |
| phosphoglycerate dehydrogenase | 0.014919 | 0.15883 |
| pyruvate:ferredoxin (flavodoxin) oxidoreductase | 0.016077 | 0.15883 |
| acetate kinase | 0.016955 | 0.15883 |
| efflux RND transporter periplasmic adaptor subunit | 0.018758 | 0.15883 |
| ABC transporter ATP-binding protein | 0.020381 | 0.15883 |
| 2-hydroxyacyl-CoA dehydratase | 0.020771 | 0.15883 |
| electron transfer flavoprotein subunit alpha/FixB family protein | 0.021921 | 0.15883 |
| glutamate dehydrogenase | 0.021966 | 0.15883 |
| nucleotide exchange factor GrpE | 0.040236 | 0.26399 |
| AbrB family transcriptional regulator | 0.042126 | 0.26399 |
| 3-methyl-2-oxobutanoate dehydrogenase subunit VorB | 0.04668 | 0.27424 |
| **Vehicle vs PCB 153 (6 µM)** | | |
| **Gene Name** | **p** | **FDR** |
| rubrerythrin family protein | 0.00012314 | 0.0068173 |
| transketolase | 0.00014983 | 0.0068173 |
| peroxiredoxin | 0.00041882 | 0.010111 |
| phosphate acetyltransferase | 0.00044446 | 0.010111 |
| formyl-CoA transferase | 0.0025237 | 0.045931 |
| oxalyl-CoA decarboxylase | 0.0042002 | 0.056448 |
| PLP-dependent aminotransferase family protein | 0.0043421 | 0.056448 |
| pyruvate:ferredoxin (flavodoxin) oxidoreductase | 0.0052787 | 0.060045 |
| 3-methyl-2-oxobutanoate dehydrogenase subunit VorB | 0.011077 | 0.097299 |
| M18 family aminopeptidase | 0.012139 | 0.097299 |
| 2-hydroxyacyl-CoA dehydratase | 0.012314 | 0.097299 |
| tRNA dimethylallyltransferase | 0.012831 | 0.097299 |
| nicotinate phosphoribosyltransferase | 0.014736 | 0.099545 |
| glutamate dehydrogenase | 0.015315 | 0.099545 |
| molecular chaperone HtpG | 0.01791 | 0.10865 |
| butyryl-CoA:acetate CoA-transferase | 0.021229 | 0.11823 |
| cation-translocating P-type ATPase | 0.023231 | 0.11823 |
| heat-inducible transcription repressor HrcA | 0.024171 | 0.11823 |
| DUF2088 domain-containing protein | 0.024686 | 0.11823 |
| flavodoxin family protein | 0.03474 | 0.15806 |
| benzoyl-CoA reductase subunit D | 0.039217 | 0.16994 |
| efflux RND transporter periplasmic adaptor subunit | 0.042475 | 0.17569 |

**Table S6**. Metatranscriptomics analysis of *Lactobacillus* with POPs exposure. The differentially expressed gene orthologys for each POP was calculated using student t test. Multiple tests correction was performed by calculating False Discovery Rate-adjusted (FDR) p-value using Benjamini-Hochberg method with a threshold of p.value < 0.05.

| **Vehicle vs TCDF (6 µM)** | | |
| --- | --- | --- |
| **Gene Name** | **p** | **FDR** |
| aldo/keto_reductase | 0.0082173 | 0.16029 |
| lipid_hydroperoxide_peroxidase | 0.011054 | 0.16029 |
| MULTISPECIES:_hypothetical_protein | 0.028245 | 0.21032 |
| oxalyl-CoA_decarboxylase,_partial | 0.029009 | 0.21032 |
| hypothetical_protein | 0.037438 | 0.21714 |
| **Vehicle vs TCDD (0.6 µM)** | | |
| **Gene Name** | **p** | **FDR** |
| MULTISPECIES:_PTS_mannose_transporter_subunit_IID | 0.039224 | 0.40136 |
| aldo/keto_reductase | 0.039684 | 0.40136 |
| **Vehicle vs PCB 126 (6 µM)** | | |
| **Gene Name** | **p** | **FDR** |
| Other | 0.0039773 | 0.099432 |
| **Vehicle vs PCB 153 (6 µM)** | | |
| **Gene Name** | **p** | **FDR** |
| YSIRK_signal_domain/LPXTG_anchor_domain_surface_protein | 0.00022341 | 0.0067279 |
| MULTISPECIES:_hypothetical_protein | 0.00032819 | 0.0067279 |
| ATP-dependent_Clp_protease_ATP-binding_subunit | 0.0006381 | 0.0087207 |
| pyruvate_oxidase | 0.0013688 | 0.01403 |
| oligopeptide_ABC_transporter_substrate-binding_protein | 0.0033411 | 0.022926 |
| elongation_factor_Tu | 0.003355 | 0.022926 |
| hypothetical_protein | 0.0051606 | 0.030227 |
| 50S_ribosomal_protein_L6 | 0.0087149 | 0.044664 |
| sigma-54_modulation_protein | 0.01248 | 0.054329 |
| 50S_ribosomal_protein_L3 | 0.013251 | 0.054329 |
| 50S_ribosomal_protein_L21 | 0.015901 | 0.059267 |
| aldo/keto_reductase | 0.030218 | 0.10324 |
| Other | 0.036455 | 0.11497 |

**Table S7**. Metatranscriptomics analysis of *Bifidobacterium* with POPs exposure. The differentially expressed gene orthologys for each POP was calculated using student t test. Multiple tests correction was performed by calculating False Discovery Rate-adjusted (FDR) p-value using Benjamini-Hochberg method with a threshold of p.value < 0.05.

| **Vehicle vs TCDD (0.6 µM)** | | |
| --- | --- | --- |
| **Gene Name** | **p** | **FDR** |
| 5-formyltetrahydrofolate cyclo-ligase | 0.0031253 | 0.035626 |
| 3-isopropylmalate dehydrogenase | 0.0054809 | 0.035626 |
| 4-hydroxy-tetrahydrodipicolinate synthase | 0.0094744 | 0.041056 |
| beta galactosidase small chain | 0.017661 | 0.057397 |
| D-alanine--D-alanine ligase | 0.033588 | 0.08733 |
| **Vehicle vs PCB 126 (6 µM)** | | |
| **Gene Name** | **p** | **FDR** |
| 16S rRNA (guanine(527)-N(7))-methyltransferase RsmG | 0.016534 | 0.19006 |
| beta galactosidase small chain | 0.020006 | 0.19006 |
| **Vehicle vs PCB 153 (6 µM)** | | |
| **Gene Name** | **p** | **FDR** |
| 3-isopropylmalate dehydrogenase | 0.025974 | 0.39452 |
| camphor resistance protein CrcB | 0.030348 | 0.39452 |

**Table S8**. Metatranscriptomics analysis of *Bifidobacterium* with POPs exposure. The differentially expressed gene orthologys for each POP was calculated using student t test. Multiple tests correction was performed by calculating False Discovery Rate-adjusted (FDR) p-value using Benjamini-Hochberg method with a threshold of p.value < 0.05.

| **Vehicle vs PCB 153 (6 µM)** | | |
| --- | --- | --- |
| **Gene Name** | **p** | **FDR** |
| 1-deoxy-D-xylulose-5-phosphate synthase | 0.026949 | 0.099904 |
| hypothetical protein | 0.049952 | 0.099904 |

**Table S9.** KEGG functional pathway analysis of *Lactobacillus paracasei* with POPs exposure. Normalized LC-MS data (to internal standard) were mapped to the KEGG orthology database using MetaboAnalyst. The denominator and numerate are the number of compounds shown in each pathway and the number of compounds shown in the LC-MS data, respectively. The *P* value is estimated from global test based on hypergeometric test for the probability of having *n*-number of metabolites of a pathway in the input list, and the Holm method and FDR are applied for multiple comparisons. Here, the top-ranked pathways were selected for each comparison.

| **Vehicle vs TCDF (0.6 µM)** | | | | | | |
| --- | --- | --- | --- | --- | --- | --- |
| **Pathway Name** | **Match Status** | **p** | **-log(p)** | **Holm p** | **FDR** | **Impact** |
| Aminoacyl-tRNA biosynthesis | 4/48 | 3.9686E-06 | 12.437 | 3.3336E-04 | 3.3336E-04 | 0.16667 |
| Taurine and hypotaurine metabolism | 2/8 | 2.3144E-04 | 8.3712 | 0.019209 | 0.0097204 | 0.42857 |
| Cysteine and methionine metabolism | 2/33 | 0.0042247 | 5.4668 | 0.34642 | 0.088718 | 0.11776 |
| Glycine, serine and threonine metabolism | 2/33 | 0.0042247 | 5.4668 | 0.34642 | 0.088718 | 0.21707 |
| Phenylalanine, tyrosine and tryptophan biosynthesis | 1/4 | 0.012853 | 4.3542 | 1.0 | 0.21594 | 0.5 |
| Thiamine metabolism | 1/7 | 0.022406 | 3.7984 | 1.0 | 0.31369 | 0.0 |
| Ubiquinone and other terpenoid-quinone biosynthesis | 1/9 | 0.028734 | 3.5497 | 1.0 | 0.33479 | 0.0 |
| Phenylalanine metabolism | 1/10 | 0.031885 | 3.4456 | 1.0 | 0.33479 | 0.0 |
| **Vehicle vs TCDF (6 µM)** | | | | | | |
| **Pathway Name** | **Match Status** | **p** | **-log(p)** | **Holm p** | **FDR** | **Impact** |
| Taurine and hypotaurine metabolism | 2/8 | 3.4626E-04 | 7.9683 | 0.029086 | 0.029086 | 0.42857 |
| Thiamine metabolism | 1/7 | 0.026836 | 3.618 | 1.0 | 0.72222 | 0.0 |
| Valine, leucine and isoleucine biosynthesis | 1/8 | 0.03062 | 3.4861 | 1.0 | 0.72222 | 0.0 |
| Vitamin B6 metabolism | 1/9 | 0.034392 | 3.3699 | 1.0 | 0.72222 | 0.0 |
| **Vehicle vs TCDD (0.06 µM)** | | | | | | |
| **Pathway Name** | **Match Status** | **p** | **-log(p)** | **Holm p** | **FDR** | **Impact** |
| Taurine and hypotaurine metabolism | 2/8 | 4.8351E-04 | 7.6344 | 0.040615 | 0.040615 | 0.42857 |
| Riboflavin metabolism | 1/4 | 0.01796 | 4.0196 | 1.0 | 0.67234 | 0.5 |
| Thiamine metabolism | 1/7 | 0.031248 | 3.4658 | 1.0 | 0.67234 | 0.0 |
| Valine, leucine and isoleucine biosynthesis | 1/8 | 0.035642 | 3.3342 | 1.0 | 0.67234 | 0.0 |
| Vitamin B6 metabolism | 1/9 | 0.04002 | 3.2184 | 1.0 | 0.67234 | 0.0 |
| **Vehicle vs TCDD (0.6 µM)** | | | | | | |
| **Pathway Name** | **Match Status** | **p** | **-log(p)** | **Holm p** | **FDR** | **Impact** |
| Taurine and hypotaurine metabolism | 2/8 | 3.4626E-04 | 7.9683 | 0.029086 | 0.029086 | 0.42857 |
| Aminoacyl-tRNA biosynthesis | 2/48 | 0.013014 | 4.3418 | 1.0 | 0.54657 | 0.0 |
| Thiamine metabolism | 1/7 | 0.026836 | 3.618 | 1.0 | 0.57778 | 0.0 |
| Valine, leucine and isoleucine biosynthesis | 1/8 | 0.03062 | 3.4861 | 1.0 | 0.57778 | 0.0 |
| Vitamin B6 metabolism | 1/9 | 0.034392 | 3.3699 | 1.0 | 0.57778 | 0.0 |
| **Vehicle vs PCB 126 (0.6 µM)** | | | | | | |
| **Pathway Name** | **Match Status** | **p** | **-log(p)** | **Holm p** | **FDR** | **Impact** |
| Taurine and hypotaurine metabolism | 2/8 | 8.2459E-04 | 7.1006 | 0.069266 | 0.069266 | 0.42857 |
| Riboflavin metabolism | 1/4 | 0.023046 | 3.7702 | 1.0 | 0.61851 | 0.5 |
| Aminoacyl-tRNA biosynthesis | 2/48 | 0.029431 | 3.5257 | 1.0 | 0.61851 | 0.0 |
| Thiamine metabolism | 1/7 | 0.04002 | 3.2184 | 1.0 | 0.61851 | 0.0 |
| Valine, leucine and isoleucine biosynthesis | 1/8 | 0.04562 | 3.0874 | 1.0 | 0.61851 | 0.0 |
| **Vehicle vs PCB 126 (6 µM)** | | | | | | |
| **Pathway Name** | **Match Status** | **p** | **-log(p)** | **Holm p** | **FDR** | **Impact** |
| Taurine and hypotaurine metabolism | 2/8 | 6.9791E-05 | 9.57 | 0.0058625 | 0.0058625 | 0.42857 |
| Thiamine metabolism | 1/7 | 0.013496 | 4.3054 | 1.0 | 0.56683 | 0.0 |
| Pantothenate and CoA biosynthesis | 1/19 | 0.036348 | 3.3146 | 1.0 | 0.87584 | 0.0 |
| **Vehicle vs PCB 153 (0.6 µM)** | | | | | | |
| **Pathway Name** | **Match Status** | **p** | **-log(p)** | **Holm p** | **FDR** | **Impact** |
| Vitamin B6 metabolism | 1/9 | 0.0058065 | 5.1488 | 0.48774 | 0.48774 | 0.0 |
| **Vehicle vs PCB 153 (6 µM)** | | | | | | |
| **Pathway Name** | **Match Status** | **p** | **-log(p)** | **Holm p** | **FDR** | **Impact** |
| Thiamine metabolism | 1/7 | 0.013496 | 4.3054 | 1.0 | 0.48523 | 0.0 |
| Taurine and hypotaurine metabolism | 1/8 | 0.015414 | 4.1725 | 1.0 | 0.48523 | 0.0 |
| Vitamin B6 metabolism | 1/9 | 0.01733 | 4.0553 | 1.0 | 0.48523 | 0.0 |
| Pantothenate and CoA biosynthesis | 1/19 | 0.036348 | 3.3146 | 1.0 | 0.75072 | 0.0 |

**Table S10.** KEGG functional pathway analysis of *Bifidobacterium longum* with POPs exposure. Normalized LC-MS data (to internal standard) were mapped to the KEGG orthology database using MetaboAnalyst. The denominator and numerate are the number of compounds shown in each pathway and the number of compounds shown in the LC-MS data, respectively. The *P* value is estimated from global test based on hypergeometric test for the probability of having *n*-number of metabolites of a pathway in the input list, and the Holm method and FDR are applied for multiple comparisons. Here, the top-ranked pathways were selected for each comparison.

| **Vehicle vs TCDF (6 µM)** | | | | | | |
| --- | --- | --- | --- | --- | --- | --- |
| **Pathway Name** | **Match Status** | **p** | **-log(p)** | **Holm p** | **FDR** | **Impact** |
| [Pyrimidine metabolism](https://www.metaboanalyst.ca/MetaboAnalyst/Secure/pathway/ResultView.xhtml) | [1/39](https://www.metaboanalyst.ca/MetaboAnalyst/Secure/pathway/ResultView.xhtml) | 0.025161 | 3.6824 | 1.0 | 1.0 | 0.01584 |
| **Vehicle vs TCDD (0.06 µM)** | | | | | | |
| **Pathway Name** | **Match Status** | **p** | **-log(p)** | **Holm p** | **FDR** | **Impact** |
| [Riboflavin metabolism](https://www.metaboanalyst.ca/MetaboAnalyst/Secure/pathway/ResultView.xhtml) | [1/4](https://www.metaboanalyst.ca/MetaboAnalyst/Secure/pathway/ResultView.xhtml) | 0.010293 | 4.5763 | 0.86458 | 0.86458 | 0.5 |
| **Vehicle vs TCDD (0.6 µM)** | | | | | | |
| **Pathway Name** | **Match Status** | **p** | **-log(p)** | **Holm p** | **FDR** | **Impact** |
| [Aminoacyl-tRNA biosynthesis](https://www.metaboanalyst.ca/MetaboAnalyst/Secure/pathway/ResultView.xhtml) | [8/48](https://www.metaboanalyst.ca/MetaboAnalyst/Secure/pathway/ResultView.xhtml) | 1.2139E-09 | 20.529 | 1.0197E-07 | 1.0197E-07 | 0.0 |
| [Valine, leucine and isoleucine biosynthesis](https://www.metaboanalyst.ca/MetaboAnalyst/Secure/pathway/ResultView.xhtml) | [3/8](https://www.metaboanalyst.ca/MetaboAnalyst/Secure/pathway/ResultView.xhtml) | 3.2038E-05 | 10.349 | 0.0026592 | 0.0013456 | 0.0 |
| [Glutathione metabolism](https://www.metaboanalyst.ca/MetaboAnalyst/Secure/pathway/ResultView.xhtml) | [2/28](https://www.metaboanalyst.ca/MetaboAnalyst/Secure/pathway/ResultView.xhtml) | 0.02505 | 3.6869 | 1.0 | 0.7014 | 0.09582 |
| [Glycine, serine and threonine metabolism](https://www.metaboanalyst.ca/MetaboAnalyst/Secure/pathway/ResultView.xhtml) | [2/33](https://www.metaboanalyst.ca/MetaboAnalyst/Secure/pathway/ResultView.xhtml) | 0.0341 | 3.3785 | 1.0 | 0.7161 | 0.24577 |
| [Valine, leucine and isoleucine degradation](https://www.metaboanalyst.ca/MetaboAnalyst/Secure/pathway/ResultView.xhtml) | [2/40](https://www.metaboanalyst.ca/MetaboAnalyst/Secure/pathway/ResultView.xhtml) | 0.048593 | 3.0243 | 1.0 | 0.81635 | 0.0 |
| **Vehicle vs PCB 126 (0.6 µM)** | | | | | | |
| **Pathway Name** | **Match Status** | **p** | **-log(p)** | **Holm p** | **FDR** | **Impact** |
| [Pentose phosphate pathway](https://www.metaboanalyst.ca/MetaboAnalyst/Secure/pathway/ResultView.xhtml) | [1/22](https://www.metaboanalyst.ca/MetaboAnalyst/Secure/pathway/ResultView.xhtml) | 0.028195 | 3.5686 | 1.0 | 1.0 | 0.0 |
| [Pyrimidine metabolism](https://www.metaboanalyst.ca/MetaboAnalyst/Secure/pathway/ResultView.xhtml) | [1/39](https://www.metaboanalyst.ca/MetaboAnalyst/Secure/pathway/ResultView.xhtml) | 0.049705 | 3.0016 | 1.0 | 1.0 | 0.01584 |
| **Vehicle vs PCB 126 (6 µM)** | | | | | | |
| **Pathway Name** | **Match Status** | **p** | **-log(p)** | **Holm p** | **FDR** | **Impact** |
| [Pentose phosphate pathway](https://www.metaboanalyst.ca/MetaboAnalyst/Secure/pathway/ResultView.xhtml) | [1/22](https://www.metaboanalyst.ca/MetaboAnalyst/Secure/pathway/ResultView.xhtml) | 0.042006 | 3.1699 | 1.0 | 1.0 | 0.0 |
| **Vehicle vs PCB 153 (0.6 µM)** | | | | | | |
| **Pathway Name** | **Match Status** | **p** | **-log(p)** | **Holm p** | **FDR** | **Impact** |
| [Aminoacyl-tRNA biosynthesis](https://www.metaboanalyst.ca/MetaboAnalyst/Secure/pathway/ResultView.xhtml) | [8/48](https://www.metaboanalyst.ca/MetaboAnalyst/Secure/pathway/ResultView.xhtml) | 1.2139E-09 | 20.529 | 1.0197E-07 | 1.0197E-07 | 0.0 |
| [Valine, leucine and isoleucine biosynthesis](https://www.metaboanalyst.ca/MetaboAnalyst/Secure/pathway/ResultView.xhtml) | [3/8](https://www.metaboanalyst.ca/MetaboAnalyst/Secure/pathway/ResultView.xhtml) | 3.2038E-05 | 10.349 | 0.0026592 | 0.0013456 | 0.0 |
| [Glycine, serine and threonine metabolism](https://www.metaboanalyst.ca/MetaboAnalyst/Secure/pathway/ResultView.xhtml) | [2/33](https://www.metaboanalyst.ca/MetaboAnalyst/Secure/pathway/ResultView.xhtml) | 0.0341 | 3.3785 | 1.0 | 0.94332 | 0.24577 |
| [Valine, leucine and isoleucine degradation](https://www.metaboanalyst.ca/MetaboAnalyst/Secure/pathway/ResultView.xhtml) | [2/40](https://www.metaboanalyst.ca/MetaboAnalyst/Secure/pathway/ResultView.xhtml) | 0.048593 | 3.0243 | 1.0 | 0.94332 | 0.0 |
| **Vehicle vs PCB 153 (6 µM)** | | | | | | |
| **Pathway Name** | **Match Status** | **p** | **-log(p)** | **Holm p** | **FDR** | **Impact** |
| [Aminoacyl-tRNA biosynthesis](https://www.metaboanalyst.ca/MetaboAnalyst/Secure/pathway/ResultView.xhtml) | [2/48](https://www.metaboanalyst.ca/MetaboAnalyst/Secure/pathway/ResultView.xhtml) | 0.023348 | 3.7573 | 1.0 | 1.0 | 0.0 |
| [Purine metabolism](https://www.metaboanalyst.ca/MetaboAnalyst/Secure/pathway/ResultView.xhtml) | [2/65](https://www.metaboanalyst.ca/MetaboAnalyst/Secure/pathway/ResultView.xhtml) | 0.041186 | 3.1897 | 1.0 | 1.0 | 0.01745 |
| [Vitamin B6 metabolism](https://www.metaboanalyst.ca/MetaboAnalyst/Secure/pathway/ResultView.xhtml) | [1/9](https://www.metaboanalyst.ca/MetaboAnalyst/Secure/pathway/ResultView.xhtml) | 0.04562 | 3.0874 | 1.0 | 1.0 | 0.0 |

**Table S11.** Sanger sequence results for each bacterial culture used in this study.

| Theoretical Strain | Sanger Sequence | Blast Results |
| --- | --- | --- |
| *Lactobacillus paracasei* (ATCC®25303^TM^) | TACGTAGGTGGCAAGCGTTATCCGGANTTATTGGGCGTAAAGCGAGCGCAGGCGGTTTTTTAAGTCTGATGTGAAAGCCCTCGGCTTAACCGAGGAAGCGCATCGGAAACTGGGAAACTTGAGTGCAGAAGAGGACAGTGGAACTCCATGTGTAGCGGTGAAATGCGTAGATATATGGAAGAACACCAGTGGCGAAGGCGGCTGTCTGGTCTGTAACTGACGCTGAGGCTCGAAAGCATGGGTAGCGAACAGGATTAGAAACCCCGGTAGTCCCTGTCTCTTATACACATCTCCGAGCCCACGAGACANGGGACTACGNGGGTATCTAATCCNGTTCNAN | *Lactobacillus paracasei* |
| *Bacteriodes fragilis* (638R) | TACGGAGGNNCCGAGCGTTATCCGGATTTATTGGGTTTAAAGGGAGCGTAGGTGGACTGGTAAGTCAGTTGTGAAAGTTTGCGGCTCAACCGTAAAATTGCAGTTGATACTGTCAGTCTTGAGTACAGTAGAGGTGGGCGGAATTCGTGGTGTAGCGGTGAAATGCTTAGATATCACGAAGAACTCCGATTGCGAAGGCAGCTCACTGGACTGCAACTGACACTGATGCTCGAAAGTGTGGGTATCAAACAGGATTAGAAACCCCCGTAGTCCCTGTCTCTTATACACATCTCCGAGCCCACGANACANAGGNACTACNGGGGTTTCTAATCCT | *Bacteriodes fragilis* |
| *Bifidobacterium longum* (ATCC®15707^TM^) | AGGNNGCAAGCGTTATCCGGAATTATTGGGCGTAAAGGGCTCGTAGGCGGTTCGTCGCGTCCGGTGTGAAAGTCCATCGCTTAACGGTGGATCCGCGCCGGGTACGGGCGGGCTTGAGTGCGGTAGGGGAGACTGGAATTCCCGGTGTAACGGTGGAATGTGTAGATATCGGGAAGAACACCAATGGCGAAGGCAGGTCTCTGGGCCGTTACTGACGCTGAGGAGCGAAAGCGTGGGGAGCGAACAGGATTAGATACCCCGGTAGTCCCTGTCTCTTATACACATCTCCGAGCCCACGNNNNCAGGNCNTNGNGGTTTTTGTTNTNCTGTTNNNCNNCNTTTTCCTCTTCAGC | *Bifidobacterium longum* |
| *Fusobacteria nucleatum subsp. nucleatum* (ATCC®25586^TM^) | CTACCCACGCTTTCGCGCTTCAGCGTCAGTATCTGTCCAGTAAGCTGGCTTCCCCATCGGCATTCCTACAAATATCTACGAATTTCACCTCTACACTTGTAGTTCCGCTTACCTCTCCAGTACTCTAGTTACACAGTTTCCAACGCAATACAGAGTTGAGCCCTGCATTTTCACATCAGACTTACATAACCACCTAGACGCGCTTTACGCCCAATAAATCCGGATAACGCTCGTGACATACGTATTACCGCGGCGGCTGACACCTGTCTCTTATACACATCTGACGCTGCC | *Fusobacteria nucleatum subsp. nucleatum* |
| *Clostridium ramosum* (ATCC®25582^TM^) | TTTCGGGACTGAGCGTCAGTTGCAGGCCAGATCGTCGCCTTCGCCACTGGTGTTCCTCCATATATCTACGCATTTCACCGCTACACATGGAATTCCACGATCCTCTCCTGCACTCTAGCTGCCTGGTTTCTATGGCTTACTGAAGTTAAGCTTCAGGCTTTCACCACAGACCCTTGCTGCCGCCTGCTCCCTCTTTACGCCCAATAATTCCGGATAACGCTTGCCACCTACGTATTACCGCGGCGGCTGACACCTGTCTCTTATACACATCTGACGCTGCCG | *Clostridium ramosum* |
| *Bacteriodes fragilis* (ATCC®25282^TM^) | GTTATCCGGATTTATTGGGTTTAAAGGGAGCGTAGGTGGACTGGTAAGTCAGTTGTGAAAGTTTGCGGCTCAACCGTAAAATTGCAGTTGATACTGTCAGTCTTGAGTACAGTAGAGGTGGGCGGAATTCGTGGTGTAGCGGTGAAATGCTTAGATATCACGAAGAACTCCGATTGCGAAGGCAGCTCACTGGACTGCAACTGACACTGATGCTCGAAAGTGTGGGTATCAAACAGGATTAGAAACCCCGGTAGTCCCTGTCTCTTATACACATCTCCGAGCCCACGAGAC | *Bacteriodes fragilis* |


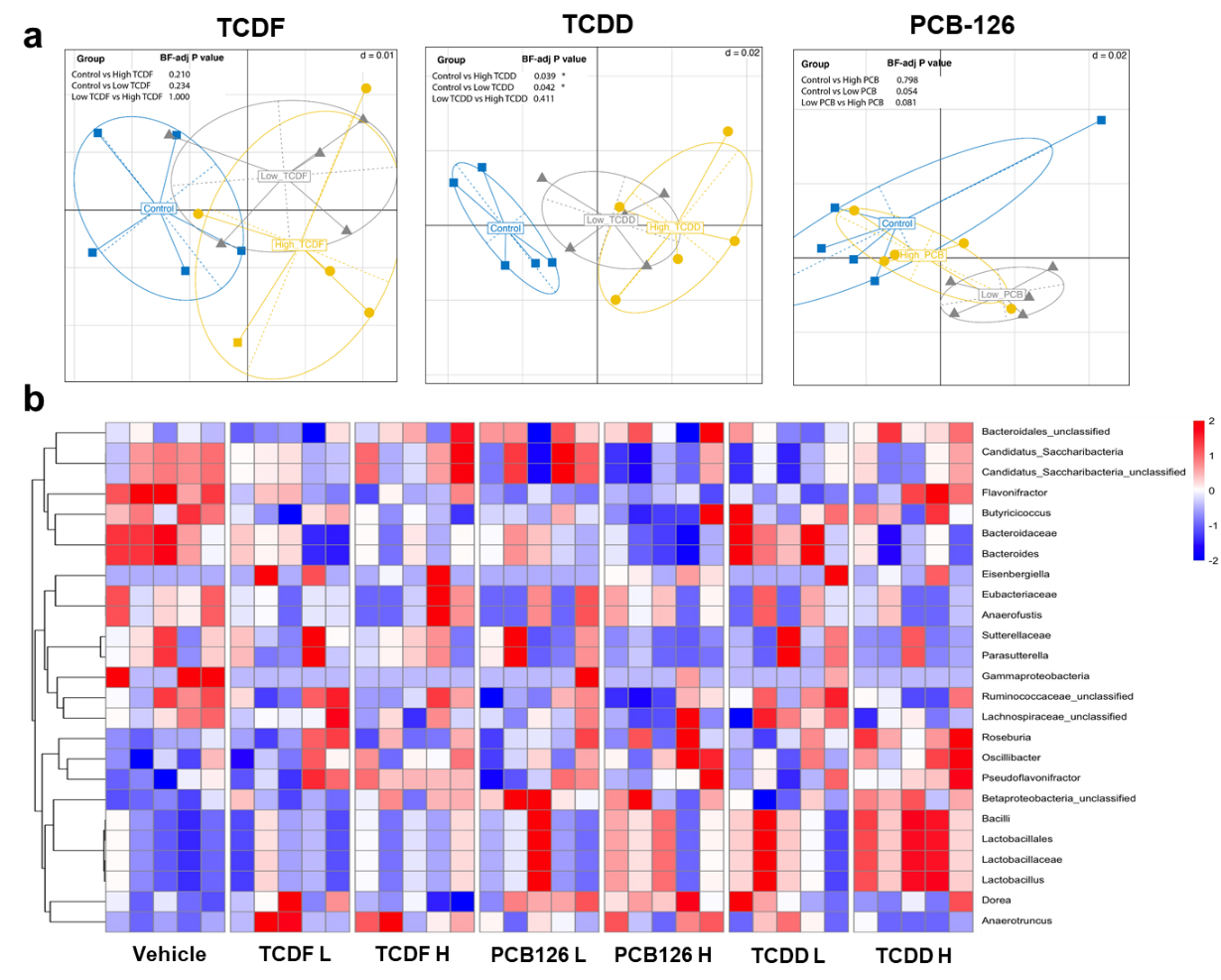


**Fig. S1. Microbial community structure analysis in isolated cecal bacteria in response to POPs exposure in vitro using 16S rRNA sequencing.** (a) Generalized Unifrac analysis of the total population of cecal bacteria with vehicle or two doses of POPs exposure for 4 h. (b) Heat map representation of the abundance of microbiota that were significantly increased (red) and decreased (blue) following two doses of POP exposure for 4 h. (n = 5 per group)


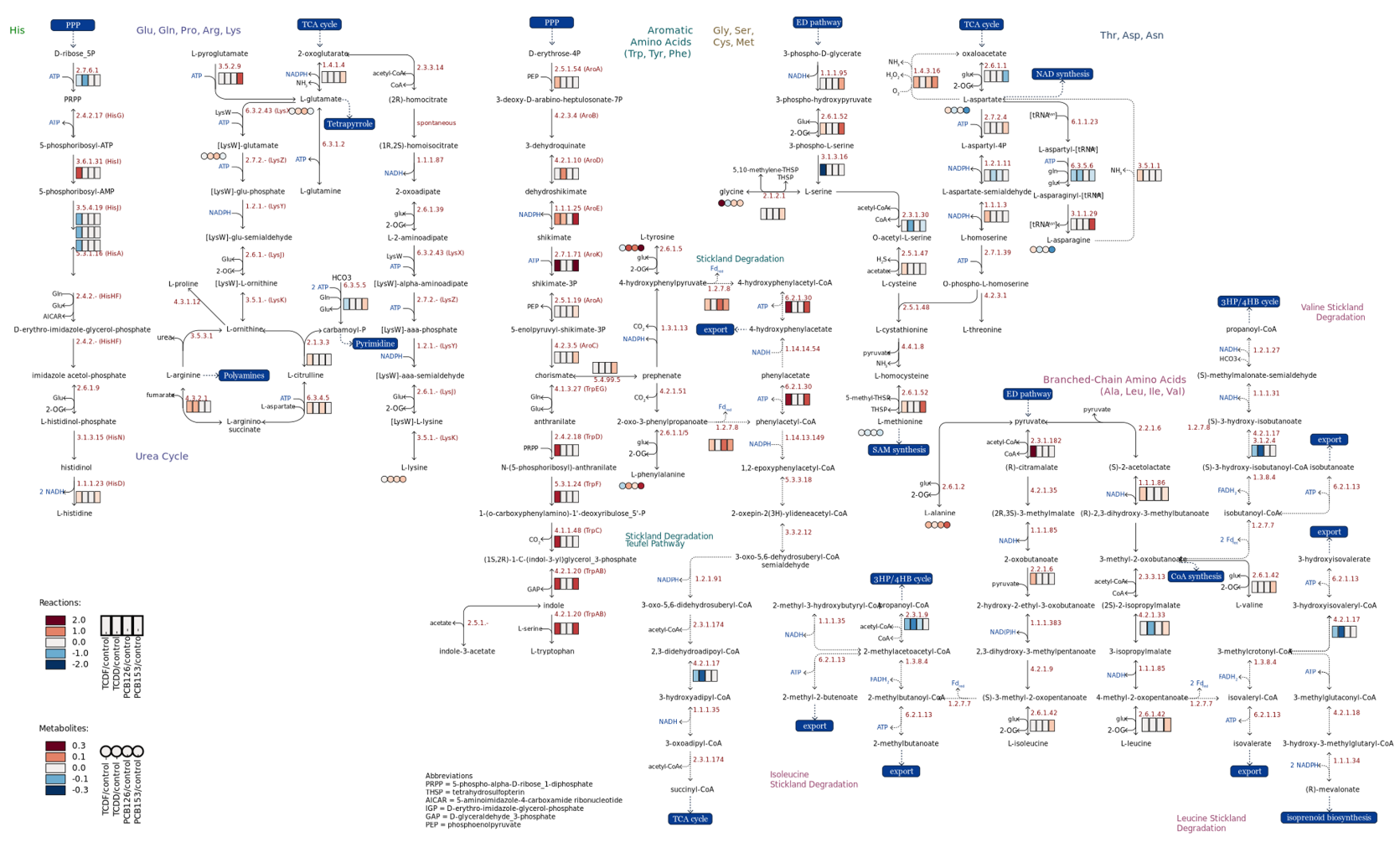


**Fig. S2.** Integrated and visualized amino acid metabolism pathway using metatranscriptomics data combined with metabolomics data. (n = 6 per group)

**
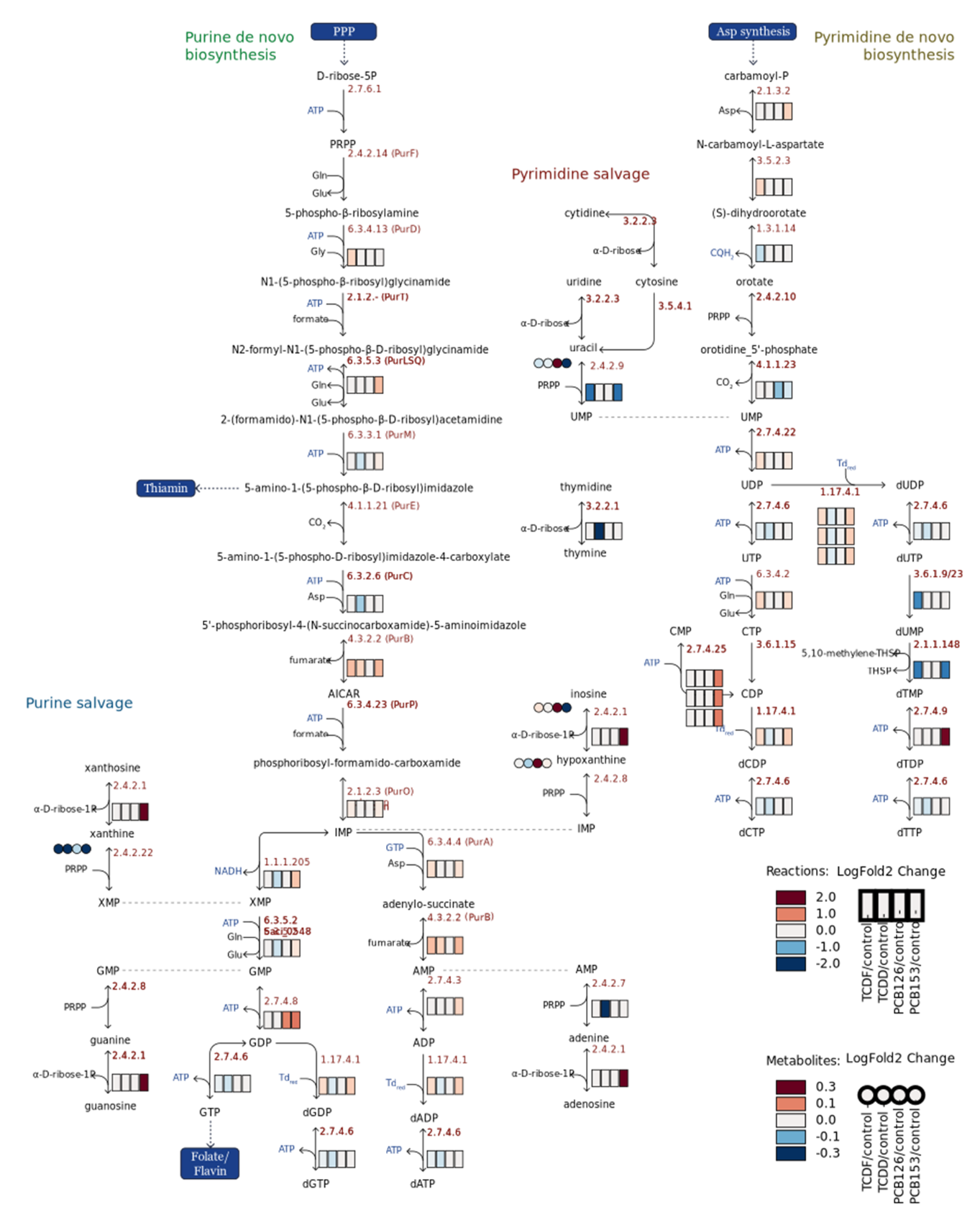
**

**Fig. S3.** Integrated and visualized nucleotide metabolism pathway using metatranscriptomics data combined with metabolomics data. (n = 6 per group)


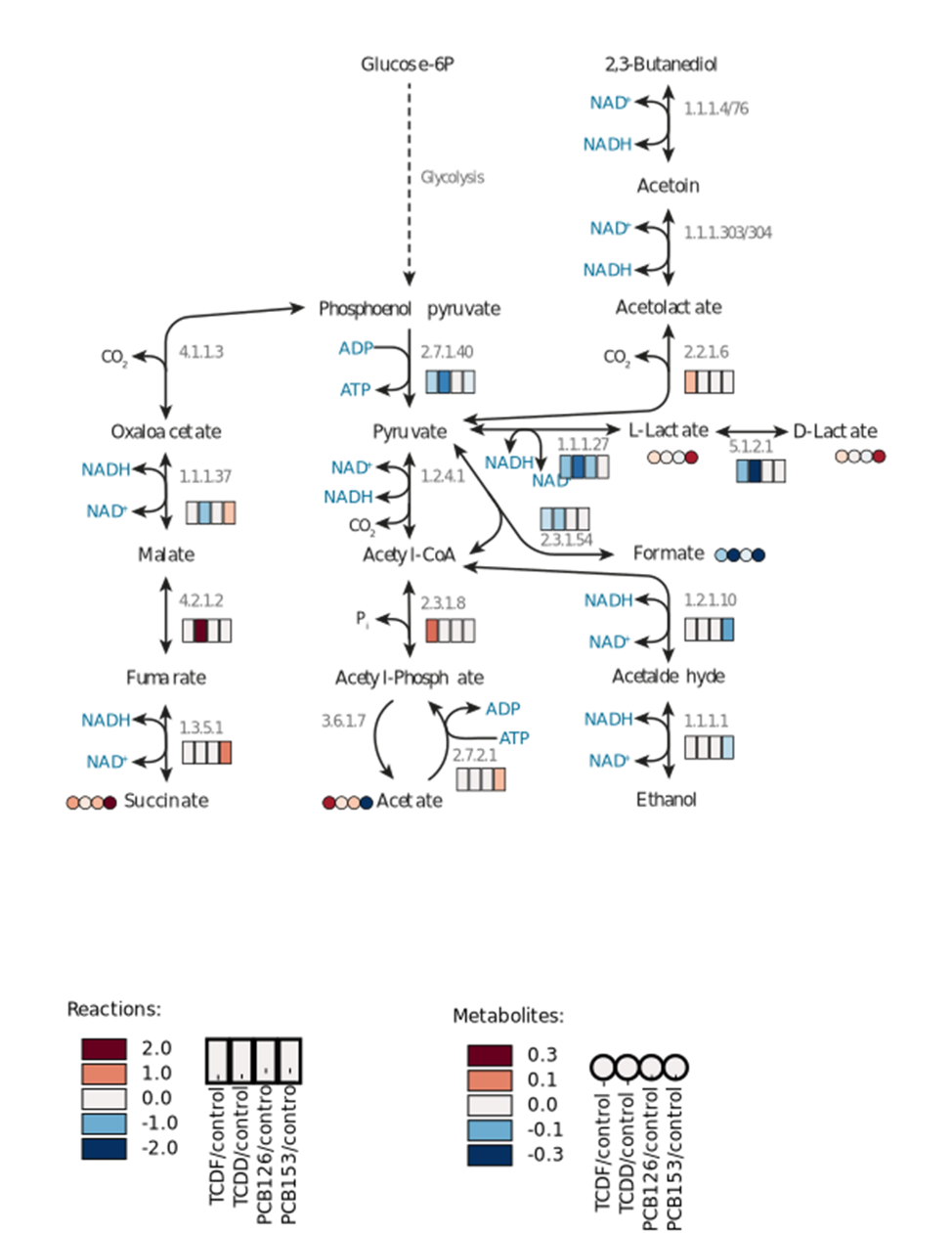


**Fig. S4.** Integrated and visualized fermentation pathway using metatranscriptomics data combined with metabolomics data. (n = 6 per group)


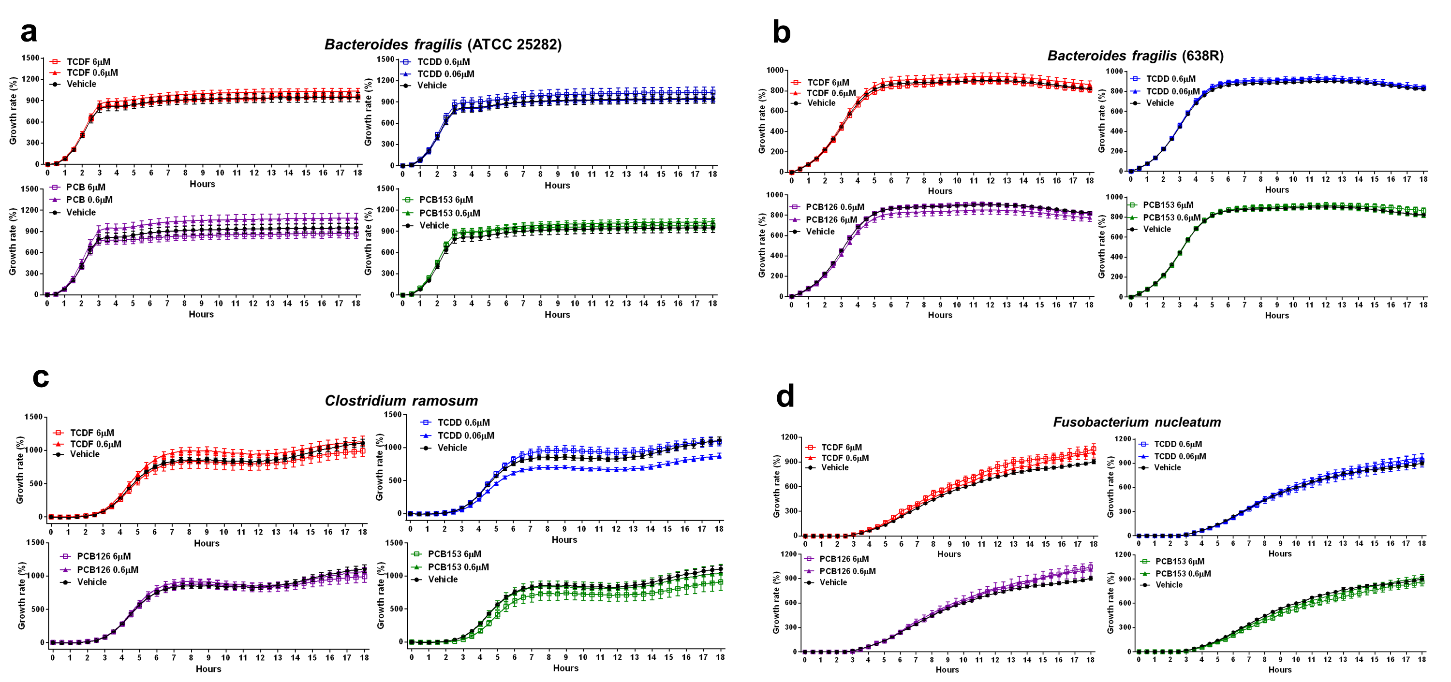


**Fig. S5. The growth rate response of individual species to POPs exposure in vitro.** Growth rate of (a) *Bacteriodes fragilis* (ATCC®25282^TM^), (b) *Bacteriodes fragilis* (638R), (Cc*Clostridium ramosum,* and (d) *Fusobacteria nucleatum* with vehicle or two doses of POPs exposure as measured by absorbance (OD_600_) over 18 hours.


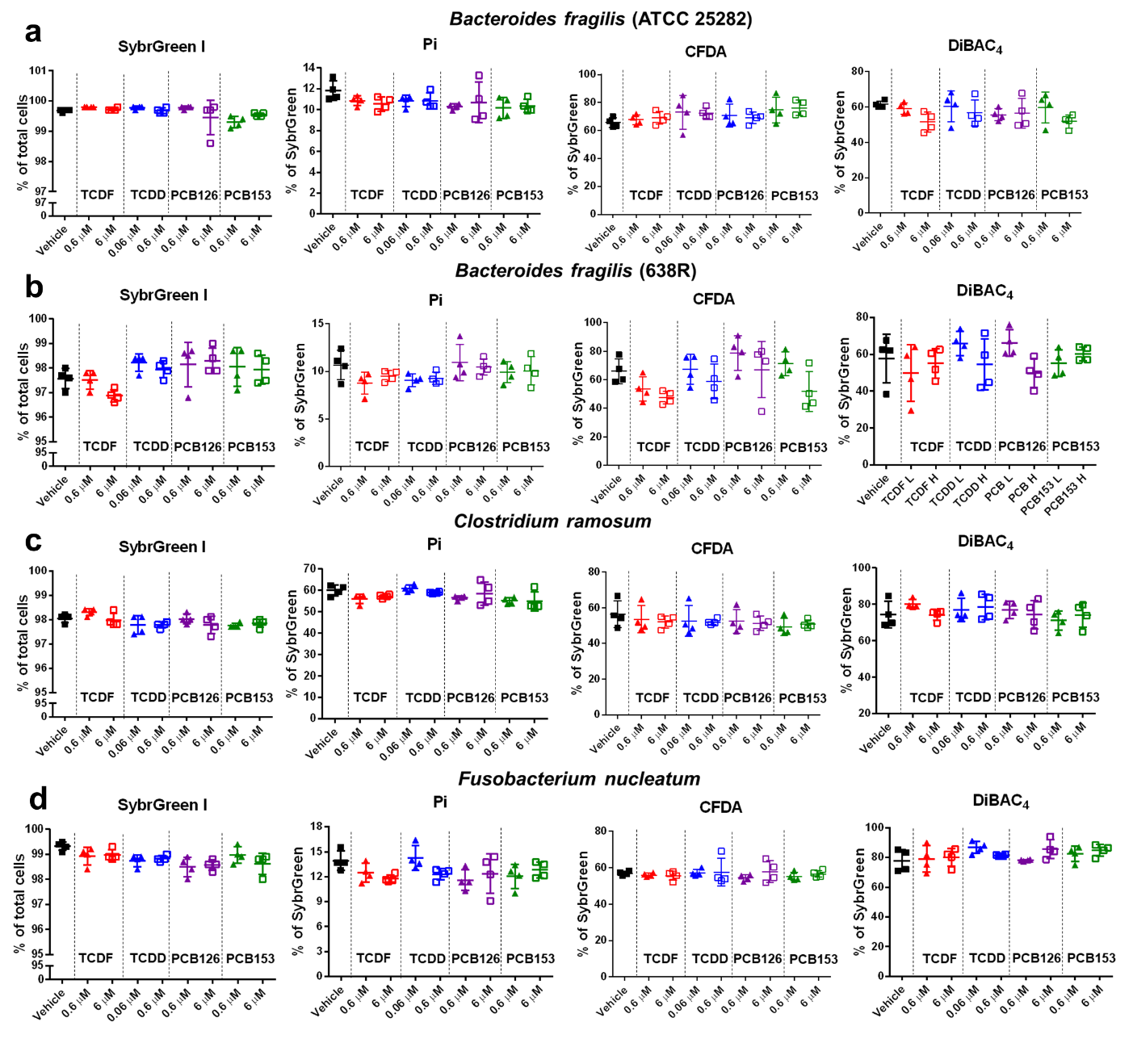


**Fig. S6. The physiological response of individual species to POPs exposure in vitro.** Flow cytometric analyses of proportions of SybrGreen, Pi, CFDA, and DiBAC_4_-stained cells from (a) *Bacteriodes fragilis* (ATCC®25282^TM^), (b) *Bacteriodes fragilis* (638R), (c) *Clostridium ramosum,* and (d) *Fusobacteria nucleatum* with vehicle or two doses of POPs exposure for 18 hours.


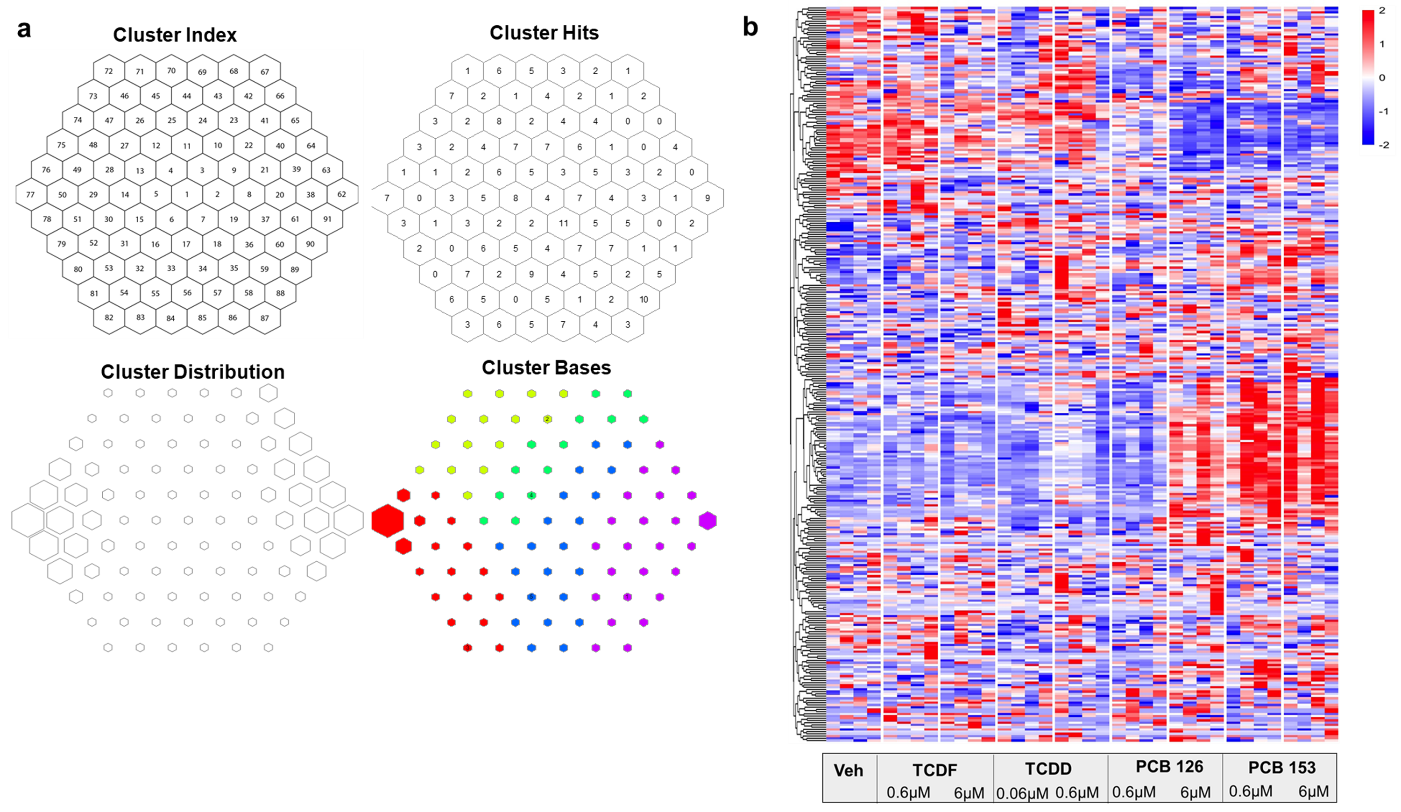


**Fig. S7. UPLC-MS/MS-based metabolomics analysis (positive mode) of lipid profiling from *Bifidobacterium longum* in response to POPs exposure in vitro.** (a) Cluster index demonstrating the supra-hexagonal self-organizing map layout. 239 assigned lipids were automatically arranged within an optimal supra-hexagon and the cluster hits correspond to the number of metabolites mapping to each node. Cluster bases display the four major metabolic clusters within the map and those with the most weight is denoted by larger hexagon. Cluster distribution demonstrating the distance between the given nodes, where the hexagon size is proportional to the distance from its neighbor. (b) Heat map representation of the abundance of lipids from Bifidobacterium longum with vehicle or two doses of POPs exposure for 18 h.


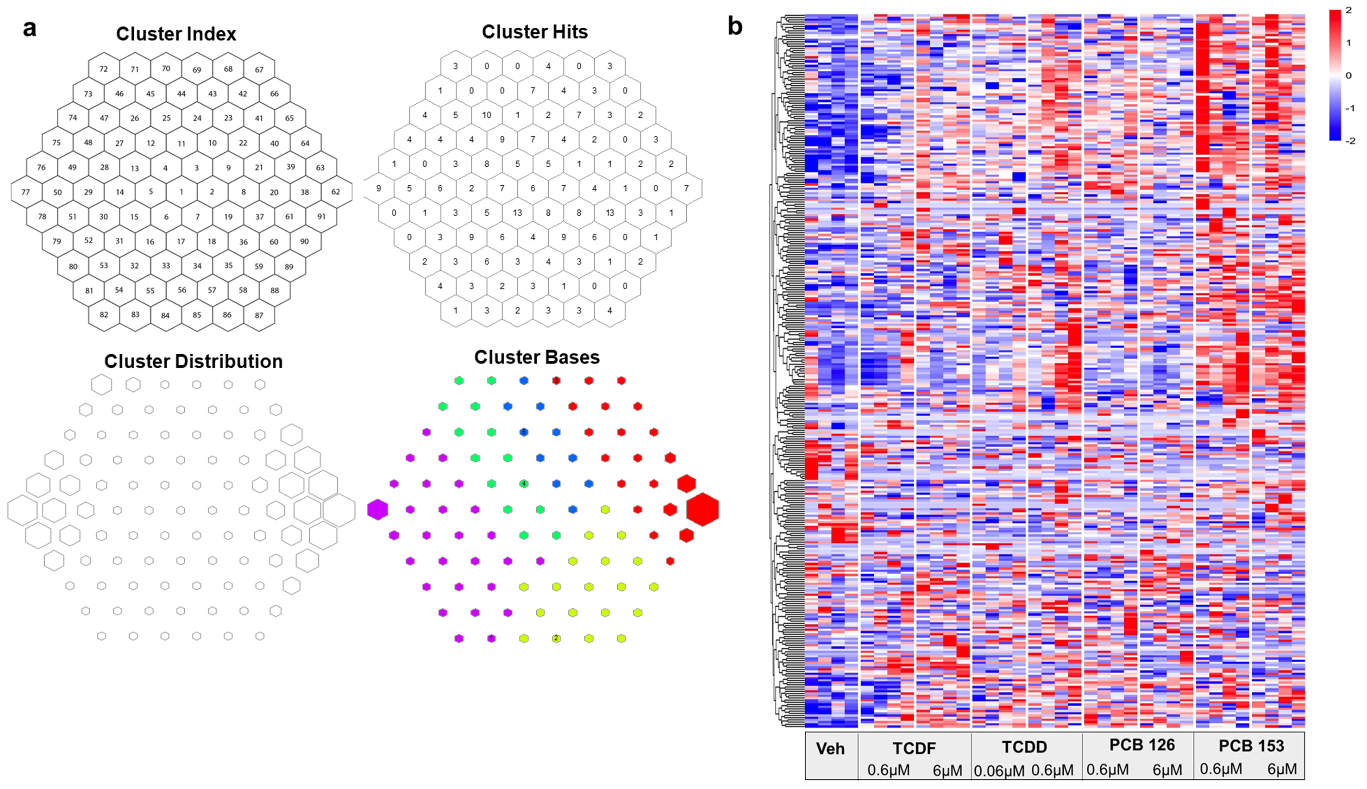


**Fig. S8. UPLC-MS/MS-based metabolomics analysis (positive mode) of lipid profiling from *Lactobacillus paracasei* in response to POPs exposure in vitro.** (a) Cluster index demonstrating the supra-hexagonal self-organizing map layout. 240 assigned lipids were automatically arranged within an optimal supra-hexagon and the cluster hits correspond to the number of metabolites mapping to each node. Cluster bases display the four major metabolic clusters within the map and those with the most weight is denoted by larger hexagon. Cluster distribution demonstrating the distance between the given nodes, where the hexagon size is proportional to the distance from its neighbor. (b) Heat map representation of the abundance of lipids from *Lactobacillus paracasei* with vehicle or two doses of POPs exposure for 18 h.


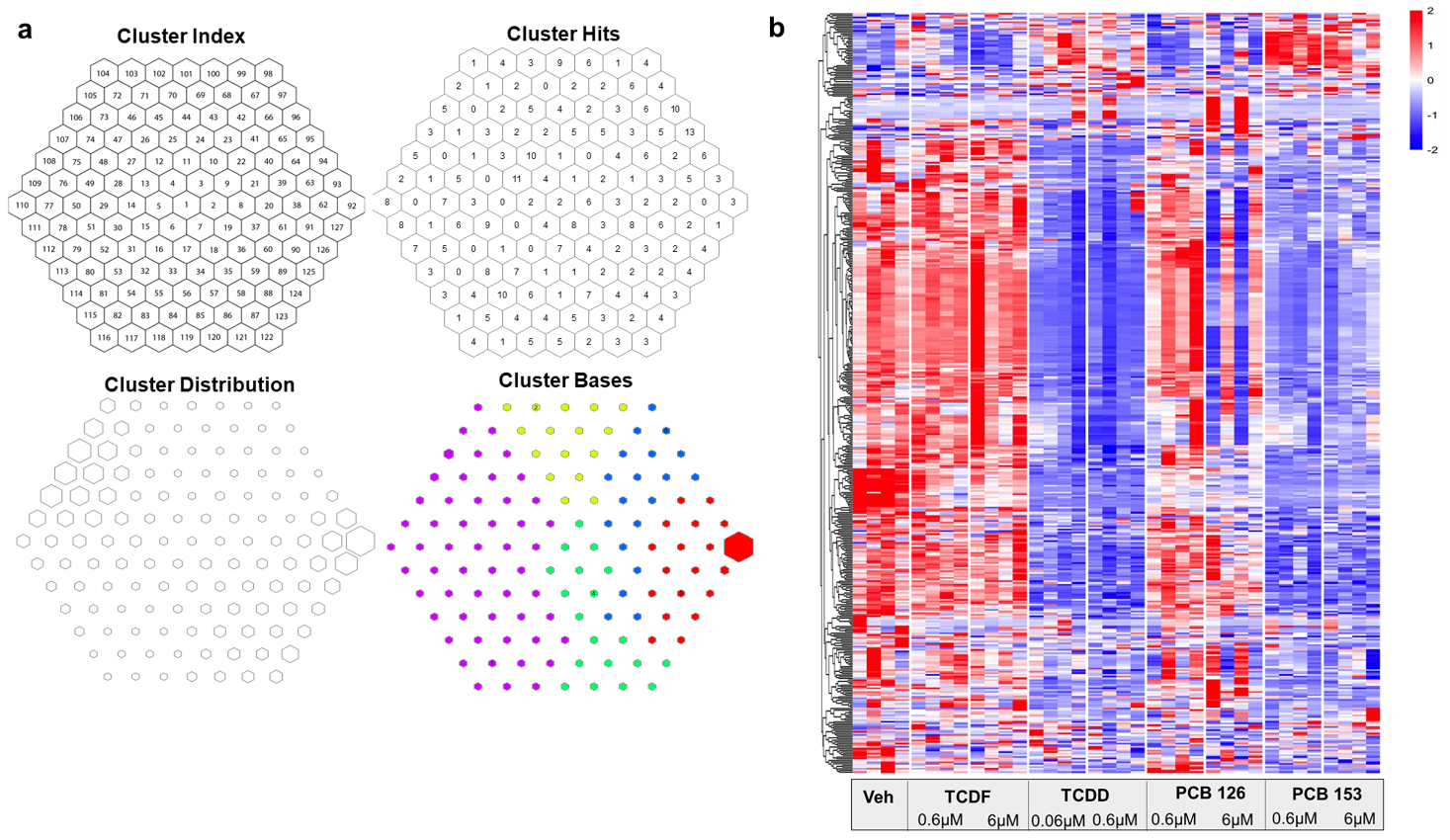


**Fig. S9. UPLC-MS/MS-based metabolomics analysis (positive mode) of lipid profiling from *Clostridium ramosum* in response to POPs exposure in vitro.** (a) Cluster index demonstrating the supra-hexagonal self-organizing map layout. 891 assigned lipids were automatically arranged within an optimal supra-hexagon and the cluster hits correspond to the number of metabolites mapping to each node. Cluster bases display the four major metabolic clusters within the map and those with the most weight is denoted by larger hexagon. Cluster distribution demonstrating the distance between the given nodes, where the hexagon size is proportional to the distance from its neighbor. (b) Heat map representation of the abundance of lipids from *Clostridium ramosum* with vehicle or two doses of POPs exposure for 18 h.


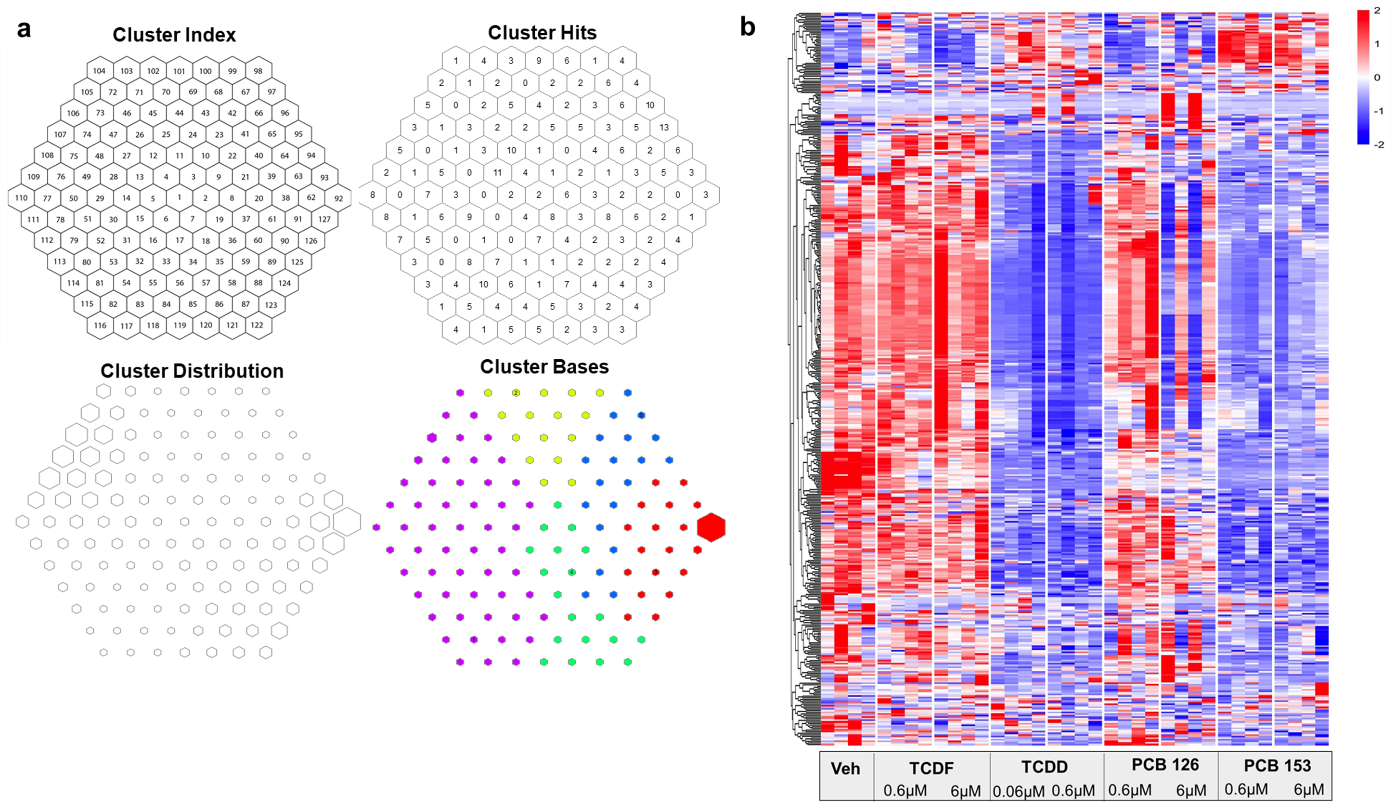


**Fig. S10. UPLC-MS/MS-based metabolomics analysis (positive mode) of lipid profiling from *Fusobacteria nucleatum* in response to POPs exposure in vitro.** (a) Cluster index demonstrating the supra-hexagonal self-organizing map layout. 1122 assigned lipids were automatically arranged within an optimal supra-hexagon and the cluster hits correspond to the number of metabolites mapping to each node. Cluster bases display the six major metabolic clusters within the map and those with the most weight is denoted by larger hexagon. Cluster distribution demonstrating the distance between the given nodes, where the hexagon size is proportional to the distance from its neighbor. (b) Heat map representation of the abundance of lipids from *Fusobacteria nucleatum* with vehicle or two doses of POPs exposure for 18 h.


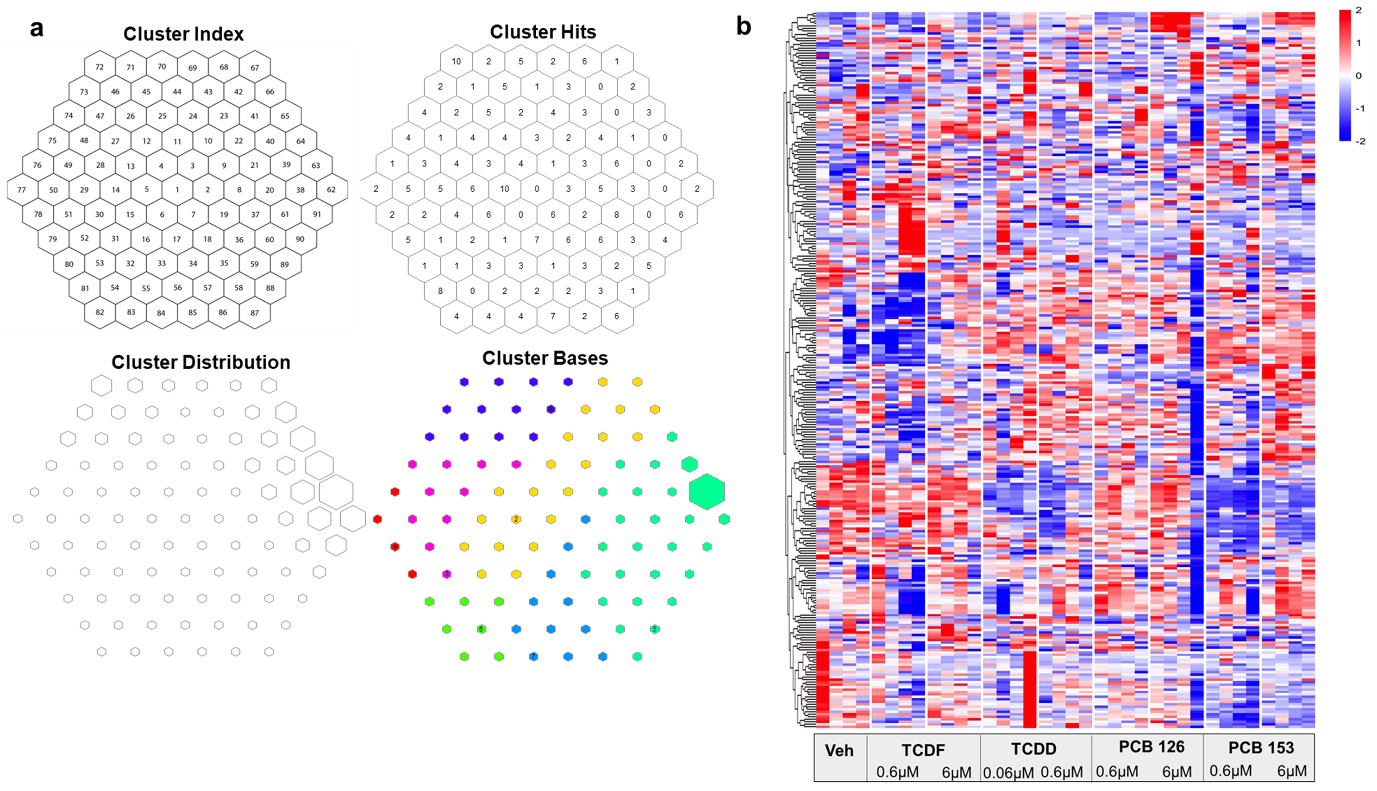


**Fig. S11. UPLC-MS/MS-based metabolomics analysis (positive mode) of lipid profiling from *Bacteriodes fragilis* (638R) in response to POPs exposure in vitro.** (a) Cluster index demonstrating the supra-hexagonal self-organizing map layout. 1122 assigned lipids were automatically arranged within an optimal supra-hexagon and the cluster hits correspond to the number of metabolites mapping to each node. Cluster bases display the six major metabolic clusters within the map and those with the most weight is denoted by larger hexagon. Cluster distribution demonstrating the distance between the given nodes, where the hexagon size is proportional to the distance from its neighbor. (b) Heat map representation of the abundance of lipids from *Fusobacteria nucleatum* with vehicle or two doses of POPs exposure for 18 h.


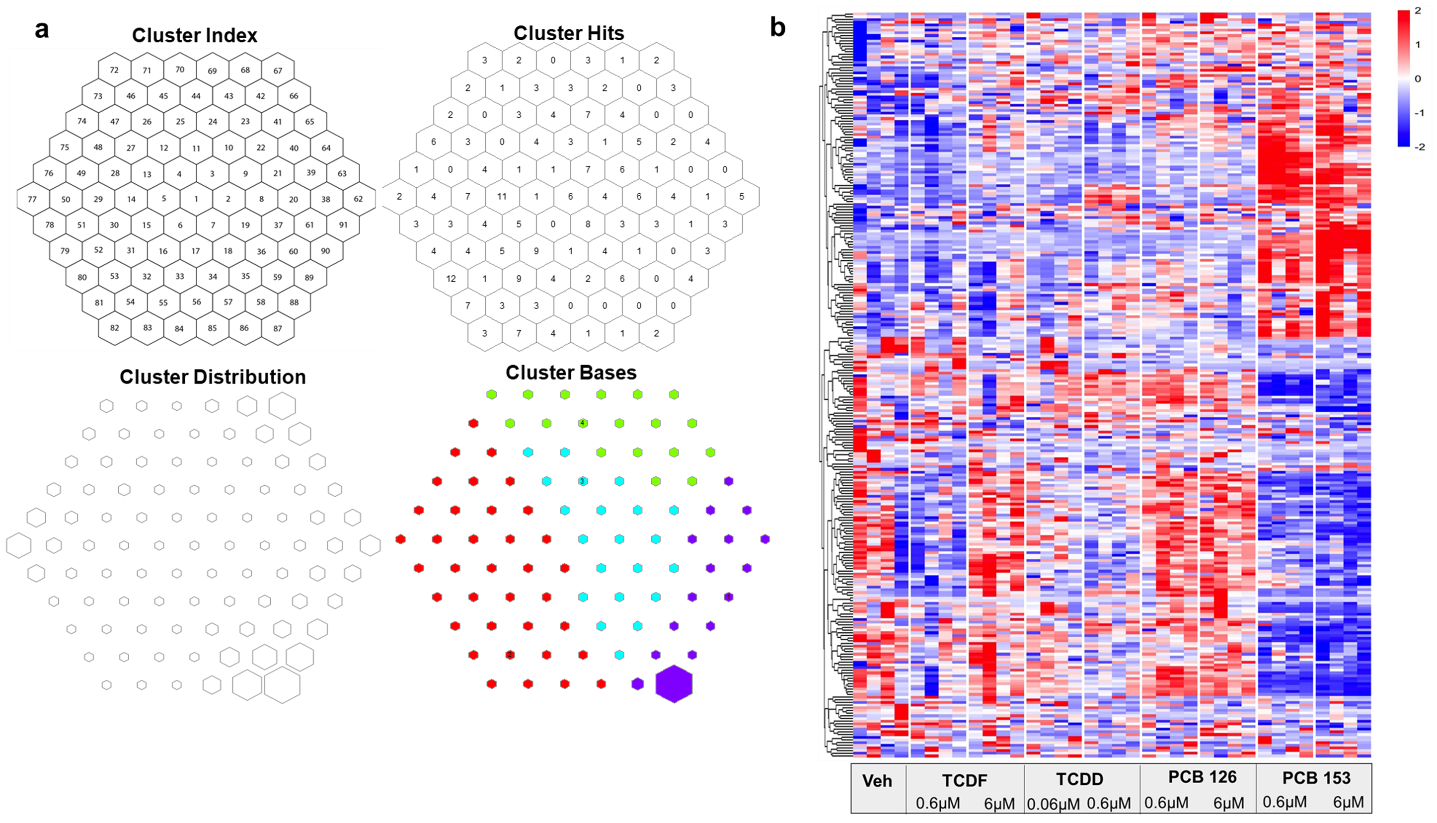


**Fig. S12. UPLC-MS/MS-based metabolomics analysis (positive mode) of lipid profiling from *Bacteriodes fragilis* (ATCC®25282^TM^) in response to POPs exposure in vitro.** (a) Cluster index demonstrating the supra-hexagonal self-organizing map layout. 1122 assigned lipids were automatically arranged within an optimal supra-hexagon and the cluster hits correspond to the number of metabolites mapping to each node. Cluster bases display the two major metabolic clusters within the map and those with the most weight is denoted by larger hexagon. Cluster distribution demonstrating the distance between the given nodes, where the hexagon size is proportional to the distance from its neighbor. (b) Heat map representation of the abundance of lipids from *Fusobacteria nucleatum* with vehicle or two doses of POPs exposure for 18 h.


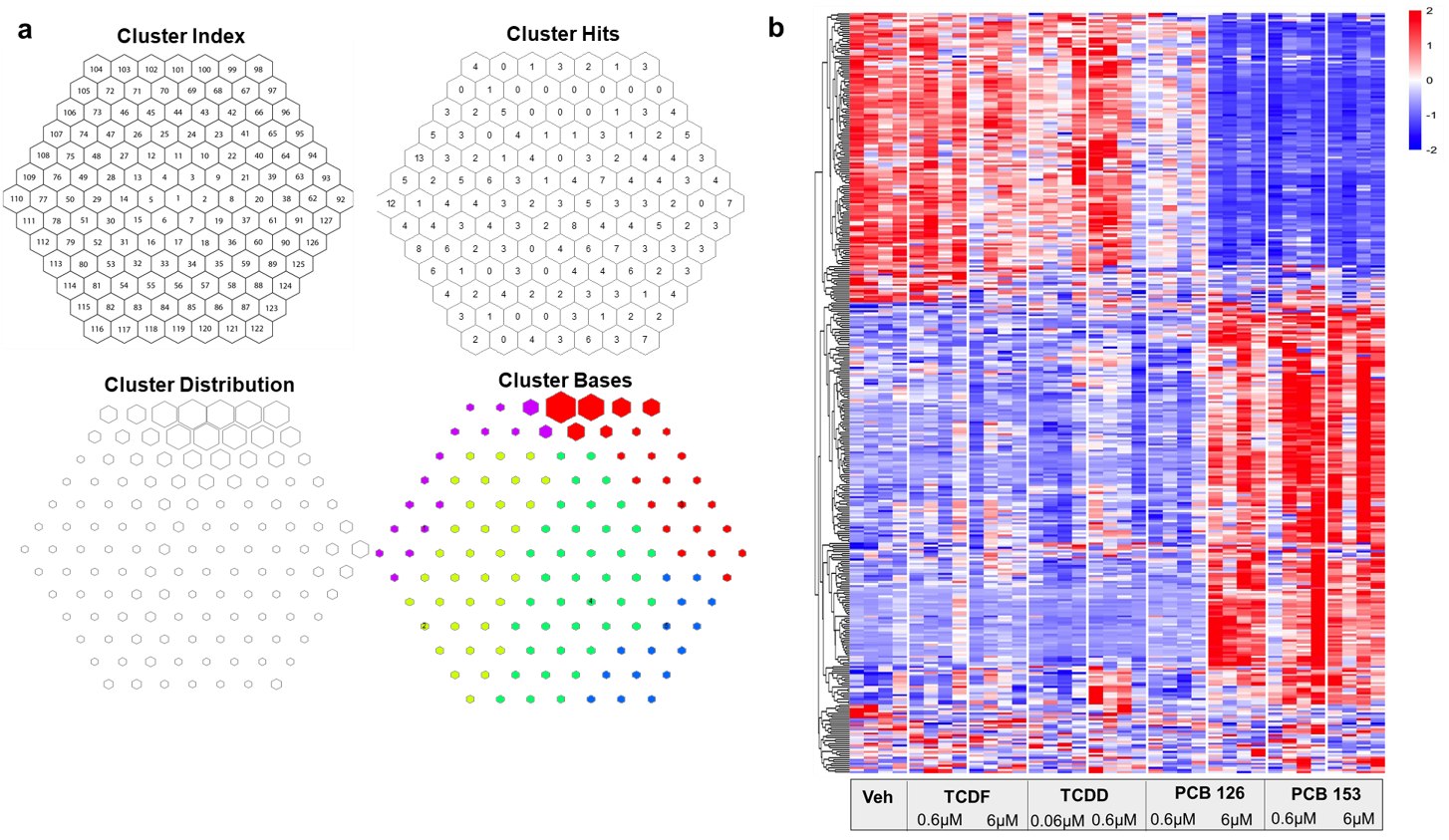


**Fig. S13. UPLC-MS/MS-based metabolomics analysis (negative mode) of lipid profiling from *Bifidobacterium longum* in response to POPs exposure in vitro.** (a) Cluster index demonstrating the supra-hexagonal self-organizing map layout. 1029 assigned lipids were automatically arranged within an optimal supra-hexagon and the cluster hits correspond to the number of metabolites mapping to each node. Cluster bases display the four major metabolic clusters within the map and those with the most weight is denoted by larger hexagon. Cluster distribution demonstrating the distance between the given nodes, where the hexagon size is proportional to the distance from its neighbor. (b) Heat map representation of the abundance of lipids from *Bifidobacterium longum* with vehicle or two doses of POPs exposure for 18 h.


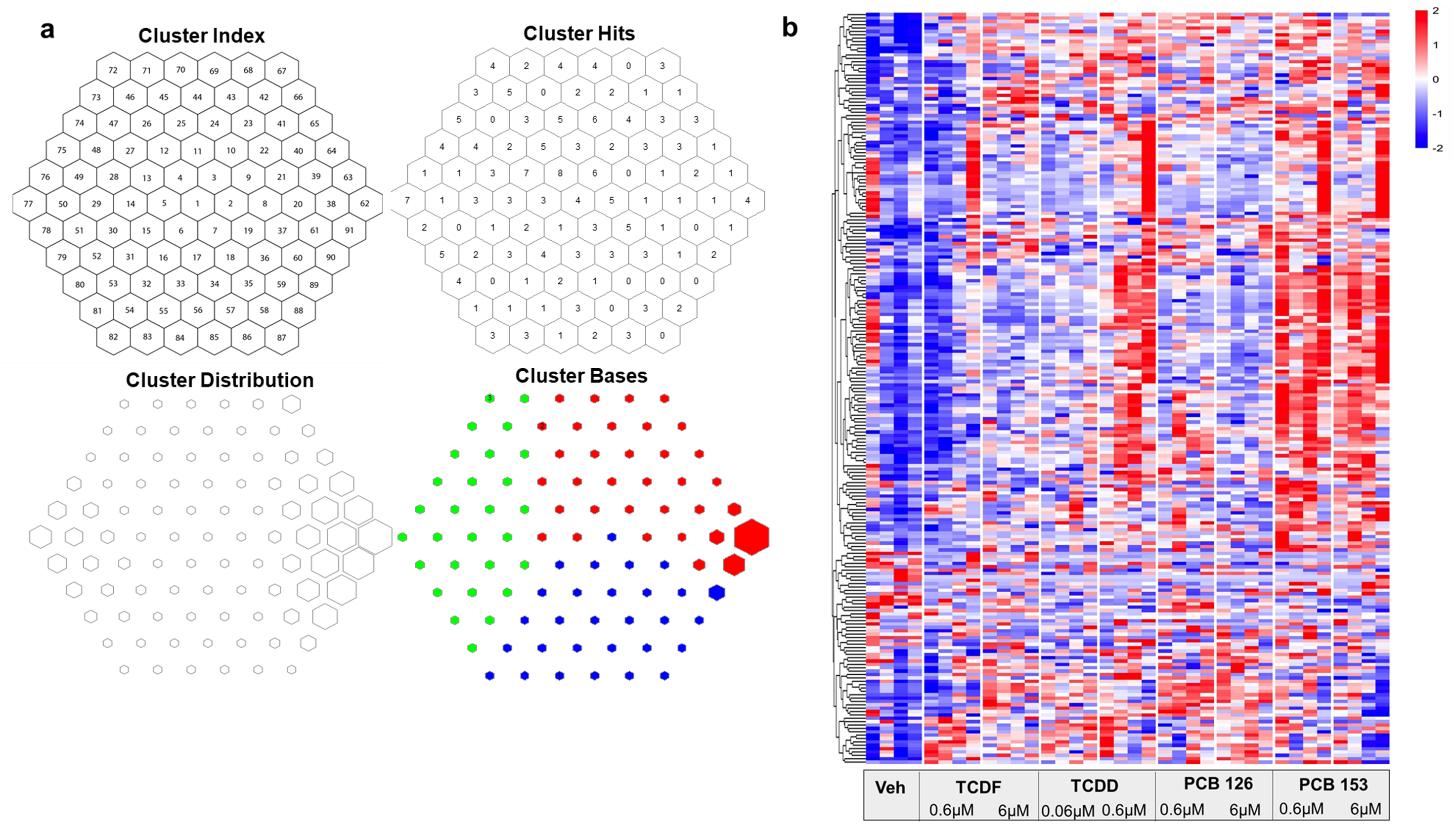


**Fig. S14. UPLC-MS/MS-based metabolomics analysis (negative mode) of lipid profiling from *Lactobacillus paracasei* in response to POPs exposure in vitro.** (a) Cluster index demonstrating the supra-hexagonal self-organizing map layout. 860 assigned lipids were automatically arranged within an optimal supra-hexagon and the cluster hits correspond to the number of metabolites mapping to each node. Cluster bases display the three major metabolic clusters within the map and those with the most weight is denoted by larger hexagon. Cluster distribution demonstrating the distance between the given nodes, where the hexagon size is proportional to the distance from its neighbor. (b) Heat map representation of the abundance of lipids from *Lactobacillus paracasei* with vehicle or two doses of POPs exposure for 18 h.


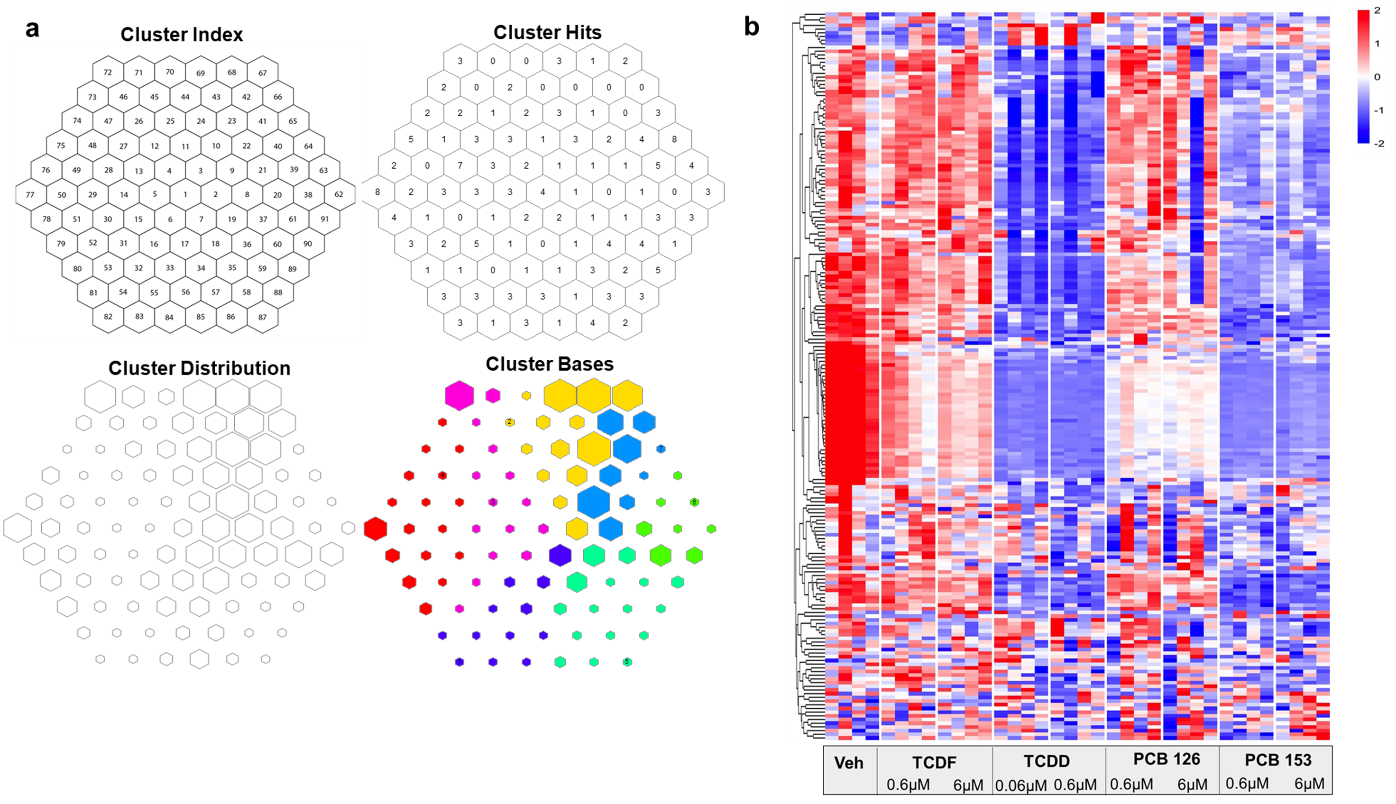


**Fig. S15. UPLC-MS/MS-based metabolomics analysis (negative mode) of lipid profiling from *Clostridium ramosum* in response to POPs exposure in vitro.** (a) Cluster index demonstrating the supra-hexagonal self-organizing map layout. 756 assigned lipids were automatically arranged within an optimal supra-hexagon and the cluster hits correspond to the number of metabolites mapping to each node. Cluster bases display the four major metabolic clusters within the map and those with the most weight is denoted by larger hexagon. Cluster distribution demonstrating the distance between the given nodes, where the hexagon size is proportional to the distance from its neighbor. (b) Heat map representation of the abundance of lipids from *Clostridium ramosum* with vehicle or two doses of POPs exposure for 18 h.


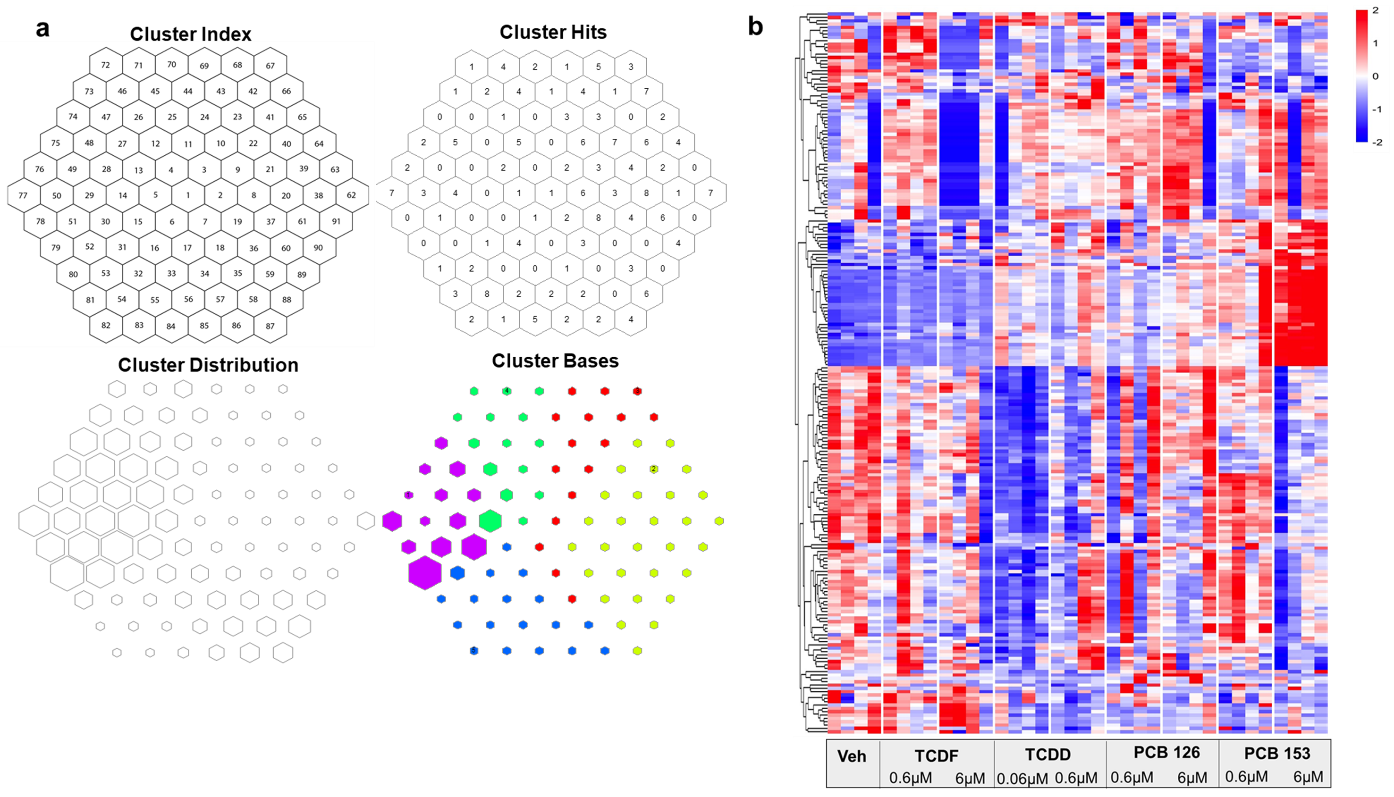


**Fig. S16. UPLC-MS/MS-based metabolomics analysis (negative mode) of lipid profiling from *Fusobacteria nucleatum* in response to POPs exposure in vitro.** (a) Cluster index demonstrating the supra-hexagonal self-organizing map layout. 1122 assigned lipids were automatically arranged within an optimal supra-hexagon and the cluster hits correspond to the number of metabolites mapping to each node. Cluster bases display the four major metabolic clusters within the map and those with the most weight is denoted by larger hexagon. Cluster distribution demonstrating the distance between the given nodes, where the hexagon size is proportional to the distance from its neighbor. (b) Heat map representation of the abundance of lipids from *Fusobacteria nucleatum* with vehicle or two doses of POPs exposure for 18 h.


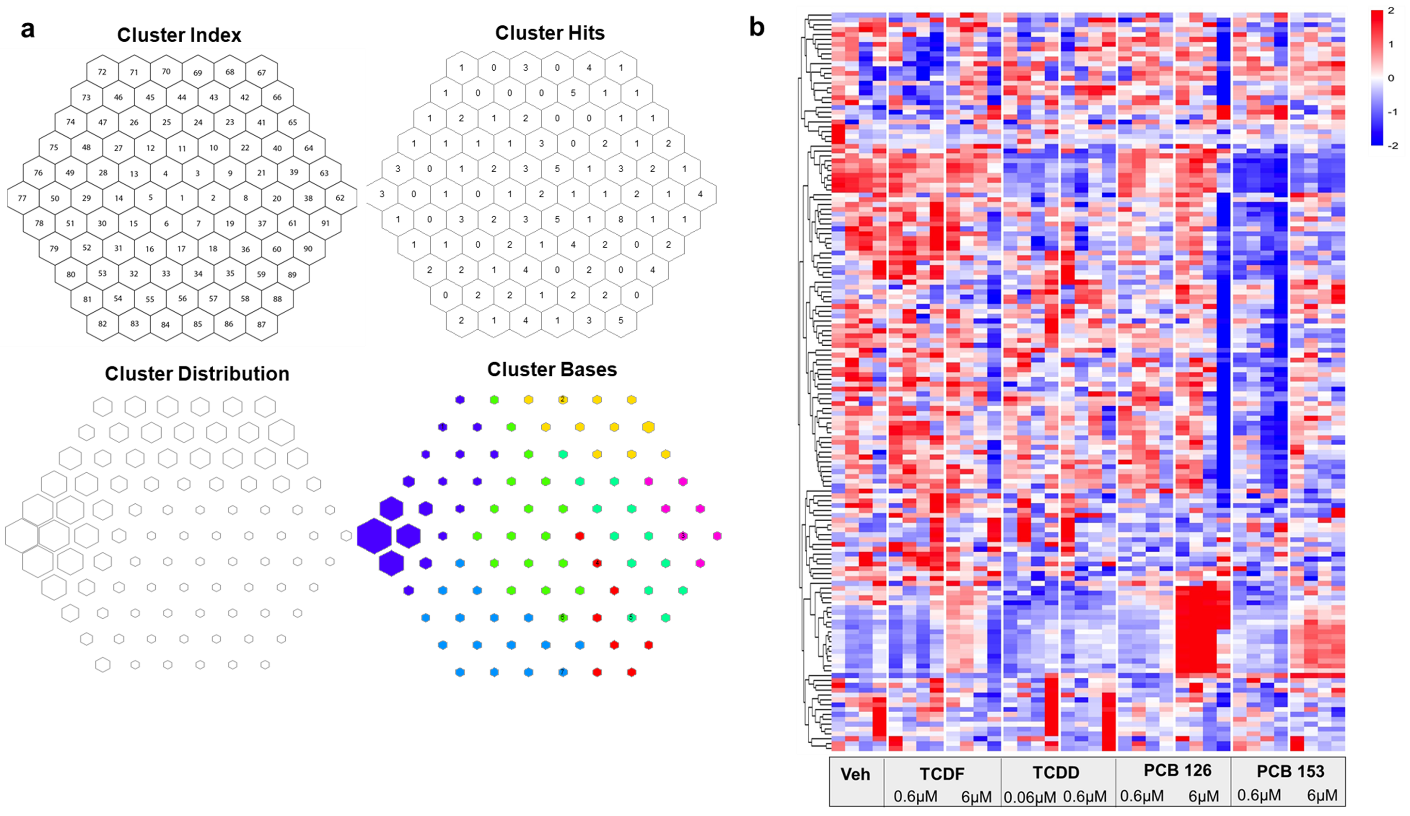


**Fig. S17. UPLC-MS/MS-based metabolomics analysis (negative mode) of lipid profiling from *Bacteriodes fragilis* (638R) in response to POPs exposure in vitro.** (a) Cluster index demonstrating the supra-hexagonal self-organizing map layout. 1122 assigned lipids were automatically arranged within an optimal supra-hexagon and the cluster hits correspond to the number of metabolites mapping to each node. Cluster bases display the four major metabolic clusters within the map and those with the most weight is denoted by larger hexagon. Cluster distribution demonstrating the distance between the given nodes, where the hexagon size is proportional to the distance from its neighbor. (b) Heat map representation of the abundance of lipids from *Fusobacteria nucleatum* with vehicle or two doses of POPs exposure for 18 h.


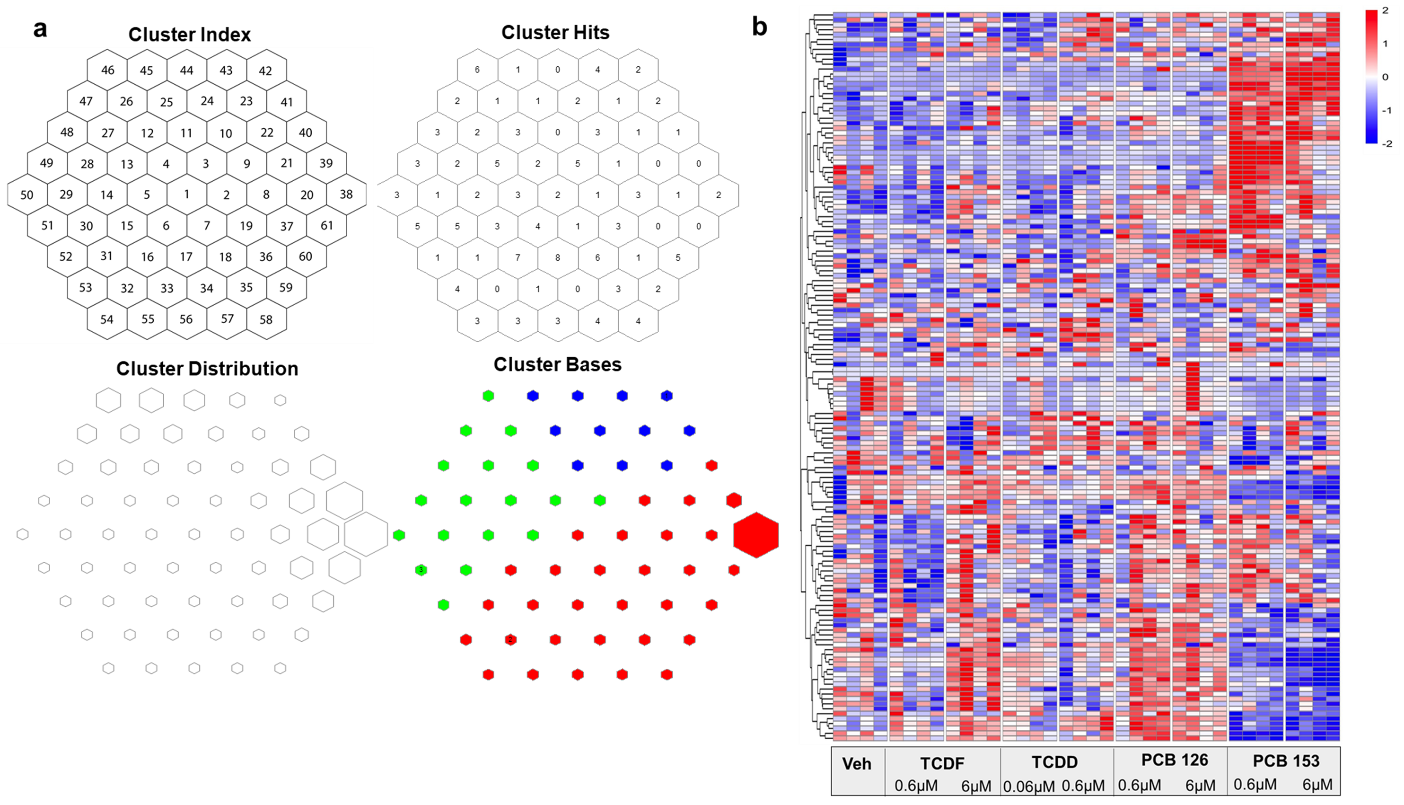


**Fig. S18. UPLC-MS/MS-based metabolomics analysis (negative mode) of lipid profiling from *Bacteriodes fragilis* (ATCC®25282^TM^) in response to POPs exposure in vitro.** (a) Cluster index demonstrating the supra-hexagonal self-organizing map layout. 1122 assigned lipids were automatically arranged within an optimal supra-hexagon and the cluster hits correspond to the number of metabolites mapping to each node. Cluster bases display the four major metabolic clusters within the map and those with the most weight is denoted by larger hexagon. Cluster distribution demonstrating the distance between the given nodes, where the hexagon size is proportional to the distance from its neighbor. (b) Heat map representation of the abundance of lipids from *Fusobacteria nucleatum* with vehicle or two doses of POPs exposure for 18 h.


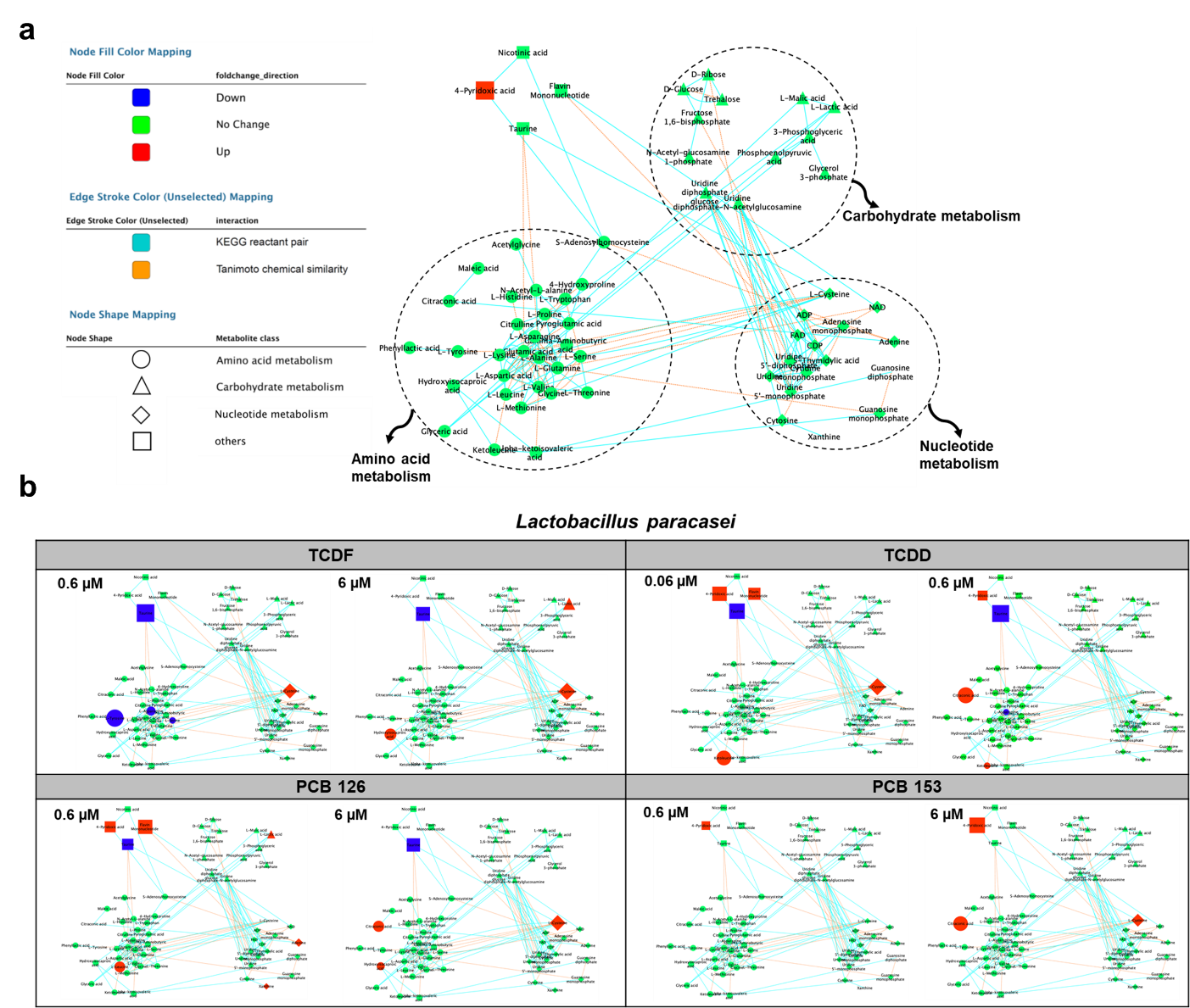


**Fig. S19. UPLC-MS/MS-based metabolomics analysis of hydrophilic metabolites from *Lactobacillus paracasei* in response to POPs exposure in vitro.** (a) 62 microbial metabolites involved in amino acid, nucleotide, and carbohydrate metabolism identified by Orbitrap LC-MS. (b) Metabolic network changes of *Lactobacillus paracasei* in response to vehicle or two doses of POPs exposure for 18 h. (n = 5 per group)


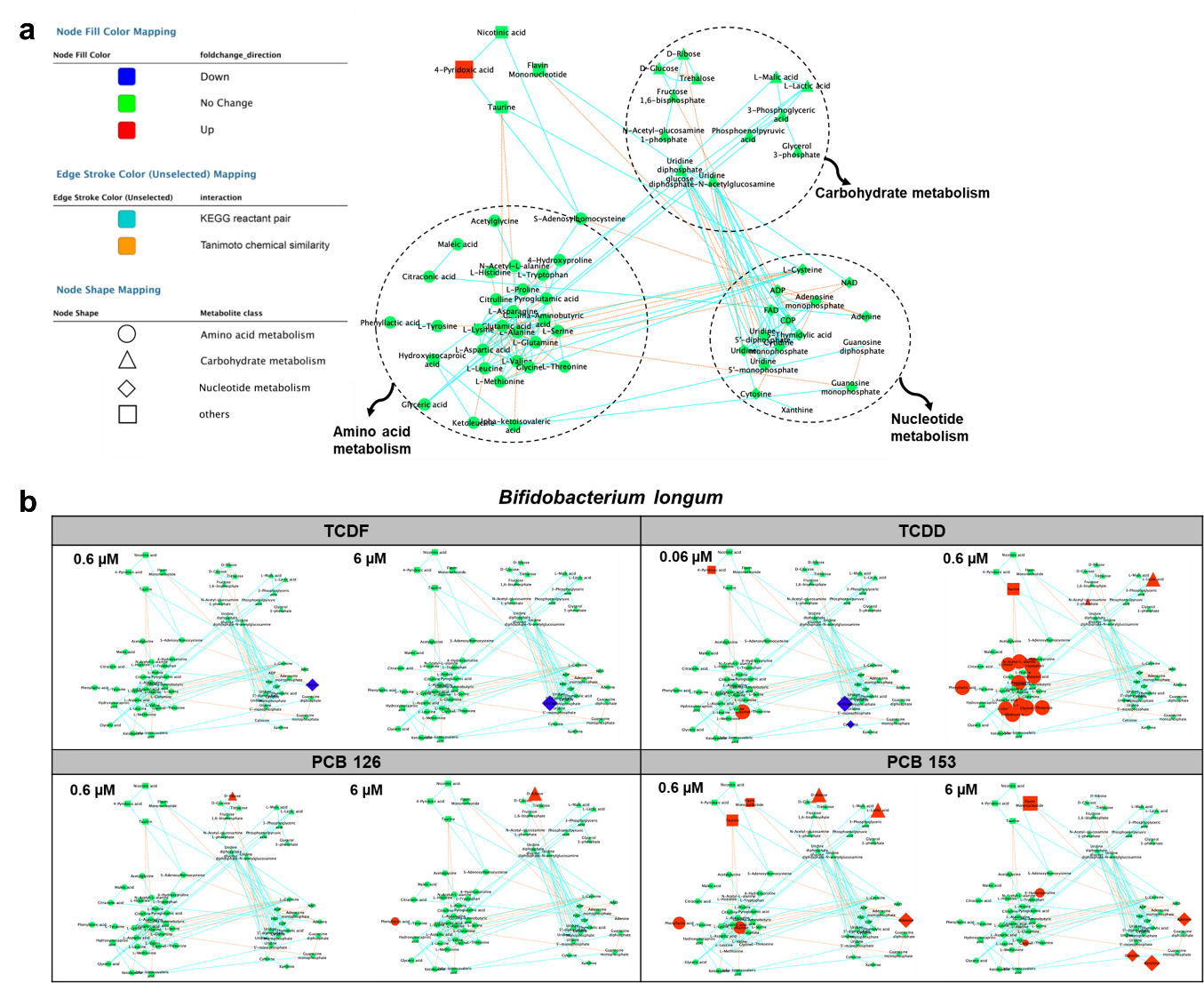


**Fig. S20. UPLC-MS/MS-based metabolomics analysis of hydrophilic metabolites from *Bifidobacterium longum* in response to POPs exposure in vitro.** (a) 62 microbial metabolites involved in amino acid, nucleotide, and carbohydrate metabolism identified by Orbitrap LC-MS. (b) Metabolic network changes of *Bifidobacterium longum* in response to vehicle or two doses of POPs exposure for 18 h. (n = 5 per group)


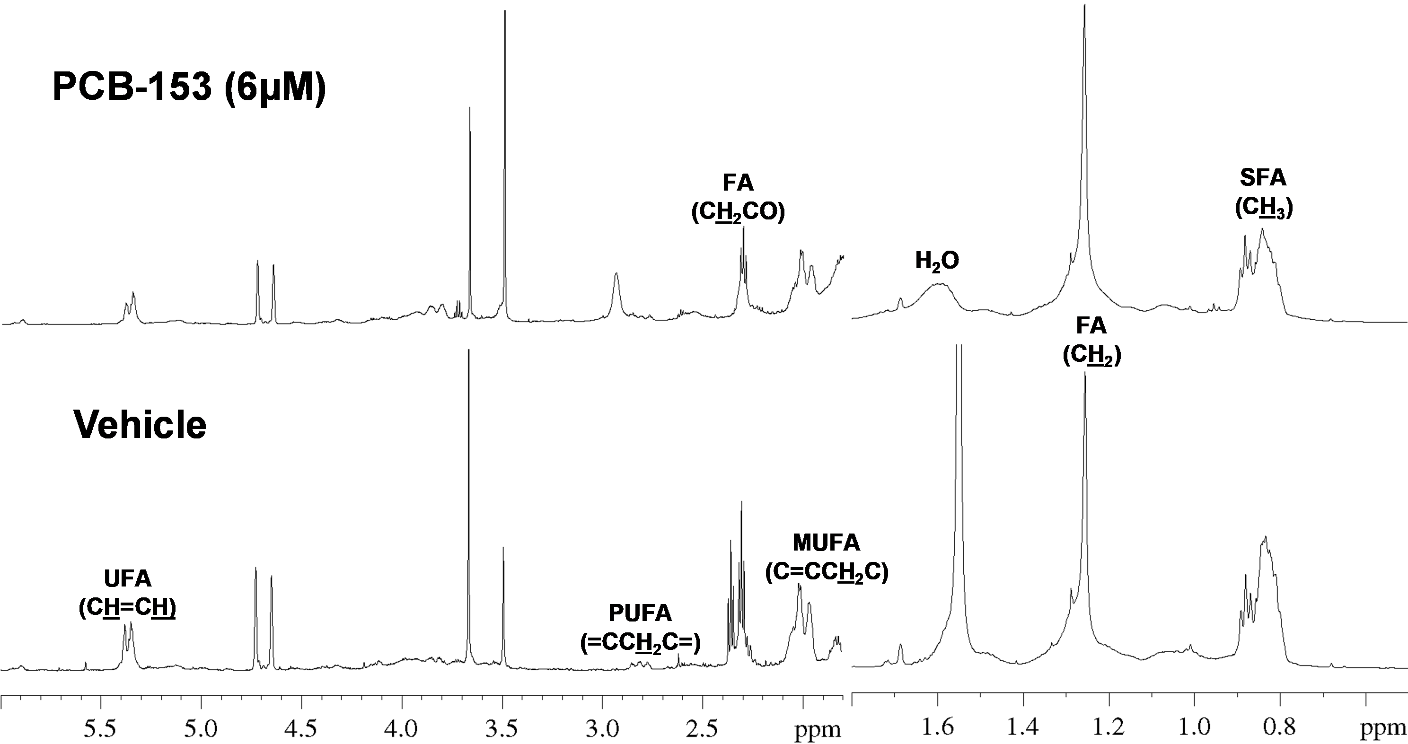


**Fig. S21.** Representative 600 MHz ^1^H NMR spectra of lipid extracts from isolated cecal bacteria with vehicle or PCB-153 exposure for 4 h.
